# The modern expansion of Dscam1 isoform diversity in *Drosophila* is linked to fitness and immunity

**DOI:** 10.1371/journal.pbio.3003383

**Published:** 2025-09-12

**Authors:** Haiyang Dong, Lili Wu, Zhechao Wang, Huiru Qu, Jinpeng Xie, Yaoqi Liu, Jingsong Lv, Nengcheng Bao, Jian Zhang, Pengjuan Guo, Ru Yan, Jiayan Fu, Jilong Shi, Ying Fu, Lei Li, Yiwen Du, Hongru Ma, Feng Shi, Jianhua Huang, Yongfeng Jin

**Affiliations:** 1 MOE Laboratory of Biosystems Homeostasis & Protection and Innovation Center for Cell Signaling Network, College of Life Sciences, Zhejiang University, Hangzhou, Zhejiang, China; 2 School of Life Sciences, Zhejiang Chinese Medical University, Hangzhou, Zhejiang, China; 3 Institute of Insect Sciences, Zhejiang University, Hangzhou, Zhejiang, China; University of Michigan, UNITED STATES OF AMERICA

## Abstract

**Drosophila melanogaster* Down Syndrome cell adhesion molecule 1* (*Dscam1*) gene encodes 38,016 diverse cell surface receptor proteins via alternative splicing, which have both nervous and immune functions. However, it remains elusive why organisms have evolved such an astonishing diversity of isoforms. Here, we show that fitness and immunity properties have driven the modern evolution of *Dscam1* isoform diversity. We assess multiple aspects of fly fitness in deletion mutants harboring exon 4, 6, or 9 clusters, respectively, reducing ectodomain isoform diversity stepwise from 18,612 to 396. All fitness-related traits generally improved as the potential number of isoforms increased; however, the magnitude of the changes varied remarkably in a variable cluster-specific manner. Correlation analysis revealed that fitness-related traits were much more sensitive to reductions in Dscam1 diversity compared to canonical neuronal self/non-self discrimination. We conclude that the role of Dscam1 isoforms in canonical neuronal self-avoidance and self/non-self discrimination is mediated by a small fraction of all isoforms (<1/10), whereas a separate role essential for other developmental contexts and resistances, likely in fitness and immunity, requires almost full isoform diversity. Thus, fitness and immunity properties, rather than canonical neuronal functions, are the dominant drivers during the modern diversification of the Dscam1 isoform. Our findings suggest that Dscam1 diversity is closely linked to adaptation and species diversification in arthropods.

## Abstract 2

The Drosophila Dscam1 gene generates tens of thousands of isoforms, yet only a small fraction supports neuronal functions. This study shows that fitness and immunity, rather than canonical neuronal roles, are the likely primary evolutionary drivers of Dscam1 isoform diversity in arthropods.

## Introduction

The *Drosophila melanogaster Dscam1* gene can generate 38,016 closely related single-transmembrane proteins of the immunoglobulin superfamily via mutually exclusive alternative splicing of 12 exon 4s, 48 exon 6s, 33 exon 9s, and 2 exon 17s, containing 19,008 ectodomains linked to one of the two alternative transmembrane segments [1]. The genomic organization of the first three variable exon clusters is conserved across all pancrustacean species investigated, but the exon number varies strikingly [2]. Fly *Dscam1* has fascinated biologists because of its potential to encode tens of thousands of distinct isoforms. Biochemical analyses show that each ectodomain preferentially exhibits isoform-specific homophilic binding: each isoform binds only to itself, but weakly or not to other isoforms both in vivo and in vitro [3–5]. Each neuron produces only 10–50 Dscam1 isoforms via probabilistic splicing, thereby providing each neuron with a unique molecular code for self-recognition in the nervous system [6–10]. Genetic studies have shown that *Dscam1* is required for the wiring of diverse types of neurons [11–25]. A canonical model suggests that Dscam1 isoforms provide the cell identity that distinguishes self from non-self neurites [8], and that 5,000 Dscam1 isoforms are essential and sufficient for this self/non-self discrimination process [15]. In our recent study, this threshold value for Dscam1 isoforms has been further narrowed down to 2,000 by finer phenotype-diversity correlations using more mutants [23]. However, this canonical mechanism does not seem to provide a reasonable explanation for what forces drove the further expansion of Dscam1 ectodomains beyond 2,000 isoforms during Pancrustacean evolution.

One speculation is that it may reflect differential splicing outcomes in various cells. For example, some cells may exhibit a more striking bias in alternative splicing, thereby limiting access to the full isoform diversity [6,10,26]. Alternatively, Dscam1 isoforms may play a role in mediating cell recognition in other nervous developmental contexts. For example, Dscam1 isoforms have been reported to be required for the collateral formation of mechanosensory axons [14,21], as well as an essential requirement for the formation of new dendritic branches in motoneurons [27]. In addition, considerable studies have shown that Dscam1 contributes to immune defenses in a variety of insects and crustaceans under certain circumstances [28]. However, no obvious changes in overall Dscam1 expression or variant expression patterns were observed after exposure to bacteria in *Drosophila* [29]. Hence, the driving force behind the evolutionary expansion of a much larger Dscam1 isoform repertoire remains elusive.

In this study, we demonstrate that the modern evolution of *Dscam1* isoform diversity is mainly driven by fitness properties rather than canonical neuronal self/non-self discrimination. We assessed how Dscam1 isoform diversity affects various aspects of fly fitness in deletion mutants harboring exon 4, 6, or 9 clusters, respectively, reducing ectodomain isoform diversity stepwise from 18,612 to 396. Comparative correlation analyses indicate that fitness-related traits are much more sensitive to reductions in Dscam1 diversity than canonical neuronal self/non-self discrimination. Together with our previous study [23], we conclude that the role of Dscam1 isoforms in canonical neuronal self-avoidance and self/non-self discrimination is mediated by a small fraction of all isoforms (<1/10), whereas a separate role essential for other developmental contexts and resistances, likely in fitness and immunity, requires almost full isoform diversity. Thus, fitness properties rather than canonical neuronal function are the dominant drivers during the modern diversification of the Dscam1 isoform.

## Results

### Divergence and constraints of *Dscam1* diversity during pancrustacean evolution

**Fig 1:**
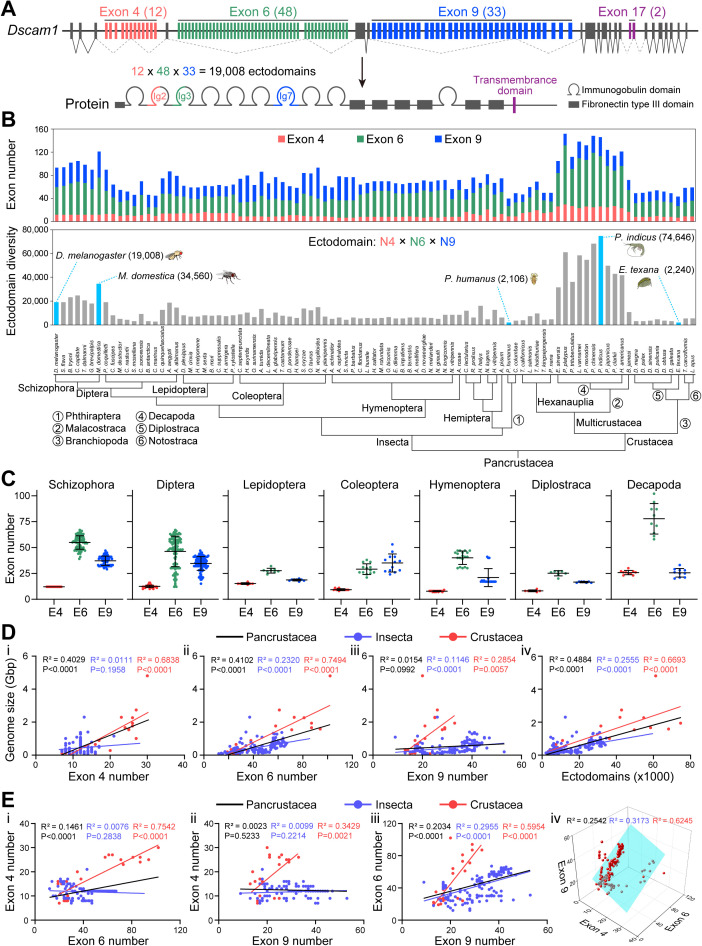
Dscam1 gene structure and isoform diversity in pancrustacean species, also see S1 Fig. **(A)** Schematic diagrams of *Drosophila melanogaster* Dscam1 gene and protein structure. The variable exons or domains are shown in color, while the constant exons or domains are shown in gray. **(B)** Phylogenetic distribution of Dscam1 isoform diversity. A phylogenetic tree of Pancrustacean species is shown in the lower panel. Upper panel: distribution of the number of exons in variable exon 4, exon 6, or exon 9 clusters in Pancrustacean species, with variable exon clusters shown in different colors. Middle panel: distribution of the number of potential ectodomains diversity, where species with a special number of diversities are highlighted. **(C)** The distribution of the number of variable exons 4, 6, and 9 in different evolutionary representative clades is shown, respectively. **(D)** Correlation analysis between the number of variable exons or the ectodomain diversity and the genome size in 178 species. **(E)** Pairwise correlation analysis of exon numbers between variable exon clusters, and three-dimensional correlation analysis of the number of exons 4, 6, and 9. The data underlying this figure can be found in S1 Data.

To generate a global framework of *Dscam1* diversity across pancrustacean species, a genome-wide analysis of *Dscam1* homologs in representative species from each of the major clades was performed. Species representing six clades of the order Insecta and three clades of the class Crustacea were examined (S1 Table)*.* These organisms constitute some of the major taxonomic groups of the pancrustacean class that last shared a common ancestor ~550 million years ago [30]. The genomic organization of the first three exon clusters was found to be well conserved across all 178 pancrustacean Dscam1s investigated, but the exon 4, 6, or 9 clusters varied strikingly in size (Figs 1A, 1B, and S1). The number varied from 7 to 30 in the variable exon 4 cluster, 12 to 102 in the variable exon 6 cluster, and 9 to 53 in the variable exon 9 cluster (Fig 1B). Accordingly, the number of distinct ectodomains encoded by Dscam1 varies considerably, ranging from 2,106 in *Pediculus humanus* to 74,646 in *Penaeus indicus*, and the midge species (*Clunio marinus*) has the smallest ectodomain repertoires known in dipteran Dscam1 [31] (Fig 1B). Nonetheless, each species can express an extremely diverse repertoire of Dscam1 isoforms, suggesting that Dscam1 diversity is ancient and functionally important. The number of variable exon 4 is relatively constant over evolutionary time, whereas the number of variable exon 6 and 9 diverges strikingly among Insecta species. For example, all 73 members of the section Schizophora contain 12 orthologous exon 4 variants in each species. In contrast, the number of variable exons in the exon 6 and 9 clusters differed, ranging from 39 to 67 in the variable exon 6 cluster and from 29 to 47 in the variable exon 9 cluster (Figs 1B and S1). Furthermore, the number of variable exon 4 in midges was comparable to that in other dipteran species. In contrast, the Antarctic midge (*Belgica antarctica*) contains only 12 variable exon 6s, only a quarter of the number found in *D. melanogaster* (Figs 1B and S1). A similar evolutionary trend of exon 4, 6, and 9 clusters is observed in Diptera, Lepidoptera, Coleoptera, Hymenoptera, and the crustacean Decapoda (Fig 1C). These data suggest that exon 4, 6, and 9 clusters evolved under different speed and selection pressures, with the exon 4 cluster being the least evolutionarily active and the exon 6 cluster being the most evolutionarily active.

To investigate the evolutionary force of Dscam1 expansion, the correlation between Dscam1 diversity and genome size was examined. Interestingly, the number of exons 4 and 6 was significantly correlated with genome size in pancrustacean species. Furthermore, a more significant correlation was observed between the number of Dscam1 ectodomains and genome size. In contrast, the number of exon 9 showed a poor correlation with genome size (Fig 1D). To further define these correlations, we separated insect and crustacean species into two groups for the analysis of variable exons. The number of variable exons 6 and 9 strongly correlated with genome size in insects and crustaceans, respectively (Fig 1D). However, the number of exon 4 did not show a correlation with genome size across insect species, but it strongly correlated across crustacean species (Fig 1D). These data suggest that the variable exon 4, 6, and 9 clusters underwent different evolutionary pressures in insects and crustaceans.

To further explore the evolutionary constraints of Dscam1 diversity, we analyzed the correlations between the numbers of variable exon 4, exon 6, and exon 9. The number of the exon 4 did not significantly correlate with the number of the exon 6 and 9 in insects, but strongly correlated in crustaceans (Fig 1E). This is consistent with the different correlation of the number of exon 4 with genome sizes in insects and crustaceans. However, the expansion of the exon 6 was significantly correlated with the expansion of the exon 9 in both insects and crustaceans (Fig 1E). These data reveal a strong positive correlation among the numbers of variable exon 4, exon 6, and exon 9 in crustacean species, but less strongly in insects (Fig 1E). These data strongly suggest that exon 4, 6, and 9 clusters did not evolve independently, but expanded in a coordinated manner under different selection pressures in insects and crustaceans.

### Reducing Dscam1 diversity affects fly fecundity in a cluster-specific manner

**Fig 2:**
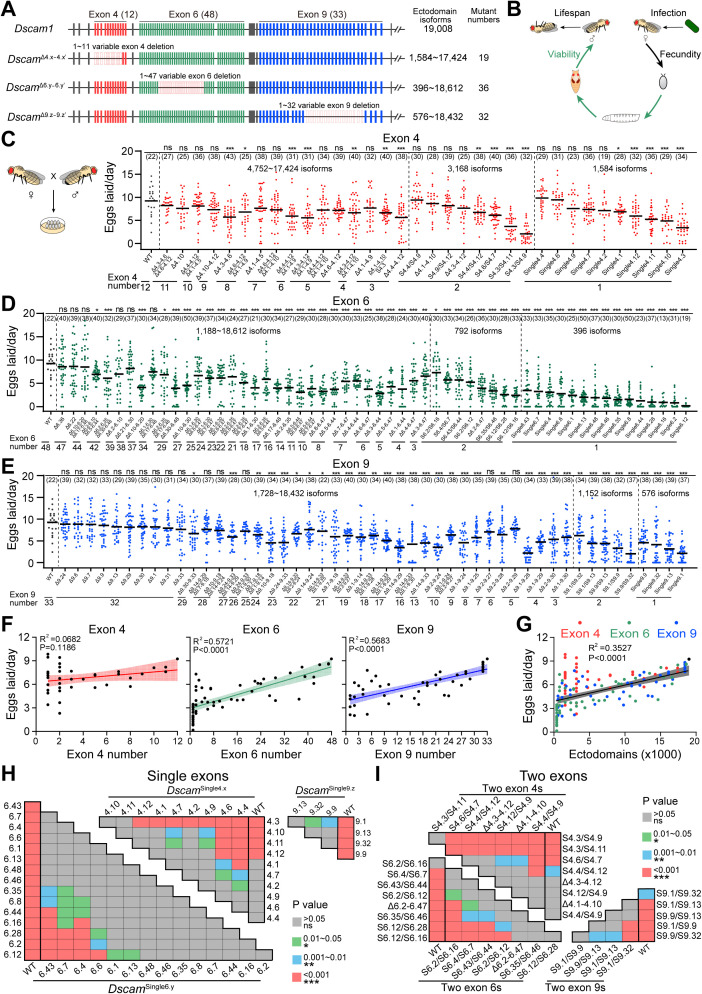
Reducing Dscam1 diversity affects fly fecundity in a cluster-specific manner, also see S2–S5 Figs. **(A)** Schematic diagram of mutants with reduced Dscam1 diversity in exon 4, 6, or 9 clusters, respectively. The potential ectodomains diversity and the number of mutants are shown on the right. **(B)** Schematic diagrams of the phenotypes assessed in each mutant. Phenotypes were assessed in different development contexts and stressed conditions, including reproduction, viability, lifespan, and immune responses. **(C–E)** The fecundity of wild-type and Dscam1 mutants with deletion of variable exon 4 (C), exon 6 (D), and exon 9 (E) were assessed, respectively. The numbers in parentheses refer to the number of mating pairs of male and female flies that were analyzed. The number of remaining variable exons of each mutant is shown at the bottom. ns, not significant; *P < 0.05; **P < 0.01; ***P < 0.001 (one-way ANOVA with Dunnett’s test). **(F)** The mean egg laid per day in each genotype was linearly fitted to the number of variable exons 4, exon 6, or exon 9, respectively. The 95% confidence intervals for the linear fit are shown with shading. **(G)** The mean egg laid per day in each genotype was linearly fitted to the ectodomain diversity. Different variable clusters of mutants are shown as dots of different colors. (F, G) Pairwise comparisons (one-way ANOVA with Tukey’s test) were performed on the fecundity of a single variable exon mutants (F) or the two variable exon mutants (G), respectively. The data underlying this figure can be found in S1 Data.

To explore the evolutionary force that drives Dscam1 isoform expansion, the correlation of Dscam1 diversity with different fly traits was analyzed. A total of 73 mutants were previously constructed using CRISPR/Cas9 technology in which various numbers of variable exons 4, 6, or 9 were deleted, respectively [23,32]. To fill out the gap, a single exon 4 (*Dscam*^∆4.10^), exon 6 (*Dscam*^∆6.22^ and *Dscam*^∆6.2-6.10^), and nine single-exon 9 deleted mutants (*Dscam*^∆9.z^) were constructed. In addition, multiple variable exon 4 deletion mutants (*Dscam*^∆4.3-4.6^ and *Dscam*^∆4.4-4.12^) were obtained from a previous study [33] (Fig 2A) (The *Dscam1* mutants used in this study are summarized in S2 Table). Furthermore, to generate a broader diversity of Dscam1 isoforms, we combined two different variable exon deletion mutants, such as *Dscam*^∆4.1-4.9^ and *Dscam*^∆4.4-4.12^, both of which contain three exon 4s, while the combination of *Dscam*^∆4.1-4.9^/*Dscam*^∆4.4-4.12^ contains six exon 4s. In this manner, 14 combination mutants were generated in the exon 4 cluster, and 16 combination mutants were generated in the exon 6 and 9 clusters, respectively. Reverse transcription PCR (RT-PCR) showed that individual partial deletions of exons 4, 6, or 9 did not affect the alternative splicing of the remaining variable exons (S2A and S2B Fig). Western blotting revealed that the overall expression levels of the partial exon deletion mutants and the combination mutants were comparable to those of the wild-type (S2C Fig). These fly mutants allowed us to assess in parallel the phenotypic consequences of reducing the diversity of exons 4, 6, and 9 in different development contexts and stress conditions, including fly reproduction, viability, lifespan, and survival reduction upon pathogen infection (Fig 2B).

The study first explored the effects of reducing Dscam1 diversity on fly fecundity throughout its life-history stages. The number of eggs laid per day by each pair of fruit flies was used as a measure of their fecundity. It was found that *Dscam*^Δ4.x-4.x′^, *Dscam*^Δ6.y-6.y′^, and *Dscam*^Δ9.z-9.z′^ adults exhibited reduced fecundity at different levels (Fig 2C–2E). Almost all *Dscam*^Δ6.y-6.y′^ and *Dscam*^Δ9.z-9.z′^ mutants exhibited reduced fecundity compared to WT. Correlation fitting showed a strong linear positive relation between fecundity and the exon 6 or exon 9 number, suggesting that almost full exon 6 or exon 9 is required to maintain normal fly fecundity (Fig 2F). However, there was a huge difference in the *Dscam*^Δ4.x-4.x′^ mutants. Overall, reducing the exon 4 number did not obviously affect the fecundity rate. Correlation fits showed a weak linear positive correlation between fecundity and exon 4 number (Fig 2F). This suggests that Dscam1 isoform diversity strongly influences fecundity in a variable cluster-specific manner. We combined these data from the mutants deleting exon 4, 6, and 9, and showed a positive correlation, albeit with high variation among various mutants (Fig 2G). When isoform diversity is reduced, the ability of mutant flies to fecundate is progressively weakened.

However, there were differences in fecundity among mutants with the same degree of isoform diversity. In the *Dscam*^Single4.x^ mutants, *Dscam*^Single4.4^, and *Dscam*^Single4.6^ did not show an obvious difference from WT in terms of fecundity, whereas the majority of *Dscam*^Single4.3^ mutant flies laid three eggs per day, compared to nine eggs per day in WT (Fig 2C). A similar trend was also shown in single exon 6 or exon 9 mutants, which showed a significant difference in terms of fecundity (Fig 2H). Not only single-exon mutants but also two-exon mutants showed a significant difference in the fecundity rate (Fig 2I). These data suggest that reducing Dscam1 diversity decreases fecundity rate in a variable exon-specific manner. In addition, it was found that in most of the mutants tested, female flies contributed more to the decline in egg laying than male flies by pairwise crossing female and male Dscam1 mutant flies with wild-type controls (S3 Fig).

To investigate the causes of decreased egg production in Dscam1 flies, we first examined the morphology of the ovaries and testes. We found that both the ovaries and testes of mutant flies were significantly smaller than those of wild-type controls (S4A and S5A Figs). Quantification of the ovary’s length and vertical projection area revealed significant reductions in both compared to wild-type flies. Additionally, ovary size positively correlated with fertility (S4B and S4C Fig). In addition, the diameter of the testes in some mutants was smaller than in the wild-type control (S5B Fig). These findings suggest that reduced Dscam1 diversity affects the development of ovaries and testes, leading to decreased egg production. Furthermore, we performed RNA-seq on ovary and testis tissue from **Dsc*am*^Single4.3^ mutants and wild-type controls. Gene Ontology (GO) enrichment analysis of differentially expressed genes revealed two reproduction-related terms: sexual reproduction and multicellular organism reproduction (S5C Fig). The differentially expressed genes found in testis tissue, such as Sfp23F and Sfp33A4, are seminal fluid proteins that are involved in reproductive processes [34] and have been validated by RT-qPCR. Other differentially expressed genes found in these two terms were further validated by RT-qPCR (S5D Fig). Taken together, our results suggest that reduced Dscam1 diversity affects ovarian and testicular development and the expression of reproduction-related genes, which may contribute to reduced egg production.

### Correlation of Dscam1 diversity with fly viability and lifespan

**Fig 3:**
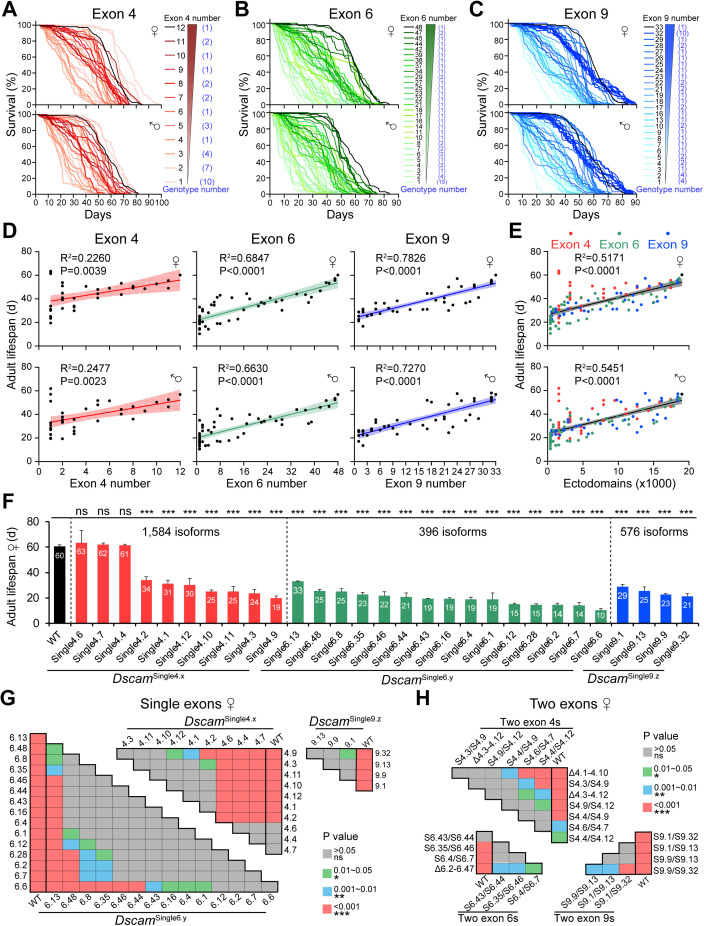
Reducing Dscam1 diversity decreases adult lifespan in a cluster-specific manner, also see S6 and S7 Figs. **(A–C)** The survival curves of male and female mutants of exon 4 (A), exon 6 (B), and exon 9 (C) are shown. The remaining number of variable exons are shown on the right. **(D)** The mean lifespan of adult flies was linearly fitted to the number of variable exons 4, exon 6, or exon 9, respectively. The 95% confidence intervals for the linear fit are shown with shading. **(E)** The mean lifespan of adult flies was linearly fitted to the ectodomain diversity. Different variable clusters of mutants are shown as dots of different colors. **(F)** The comparison of the mean female lifespan between the wild-type and *Dscam*^Single4.x^, *Dscam*^Single6.y^, and *Dscam*^Single9.z^ mutants. ns, not significant; ***P < 0.001 (one-way ANOVA with Dunnett’s test). **(G, H)** Pairwise comparisons (one-way ANOVA with Tukey’s test) were performed on the lifespan of a single variable exon mutants (G) or the two variable exon mutants (H), respectively. The data underlying this figure can be found in S1 Data.

Next, an assessment was made of the effects of reducing the number of exons 4, 6, or 9 on fly viability and growth across life-history stages. Previous studies have shown that reducing Dscam1 diversity affects fly viability in a cluster-specific manner [23,32]. We assessed the viability of variable exon deletion mutants and the Dscam1 combination mutants. The results indicated that a reduction in Dscam1 diversity led to decreased viability (S2D Fig). We then examined the effect of reduced Dscam1 diversity on fly lifespan from adulthood to death. Considering the large number of mutants, we chose to use normal lifespan instead of health span for evaluation, for simplicity. There were five groups of males or females, with 30 flies in each group. The average survival days from adulthood to death of each fly were calculated to represent the average lifespan. Through the survival curve and average lifespan calculation, almost all multiple variable exon deletion mutants had shortened lifespans compared to WT (Figs 3A–3C and S6), suggesting that maintaining normal adult lifespan requires nearly complete isoform diversity. The male and female adult lifespan was greatly extended as the number of remaining variable exons 4, 6, and 9 increased (Fig 3D). Combining these data from mutants with the deletion of exons 4, 6, and 9, a strong positive correlation was found, albeit with high variation among various mutants (Fig 3E). Further analysis showed a strong positive correlation in lifespan between male and female mutants (S7A and S7B Fig), suggesting that the effect of Dscam1 isoforms on lifespan is largely independent of sex. These data indicate that Dscam1 diversity plays an important role in regulating adult lifespan. Previous studies have shown that reduced fertility can usually increase lifespan [35,36]. However, reducing the number of Dscam1 isoforms not only reduced the fertility of flies but also shortened their lifespan, suggesting that Dscam1 isoforms play an important role in regulating both fertility and lifespan.

However, there are differences among mutants with the same degree of isoform diversity, and even several mutants with less variable exon numbers exhibit longer adult lifespan. For example, the adult lifespan of the *Dscam*^Single4.4^, *Dscam*^Single4.6^, and *Dscam*^Single4.7^ mutants was not significantly different from that of the wild-type control, whereas the average lifespan of other *Dscam*^Single4.x^ mutants was significantly lower (Figs 3F and S7C). This suggests that exon 4.4, 4.6, and 4.7 play a more important role in lifespan than other exon 4s. To investigate the underlying mechanism, we analyzed which features of exon 4 isoforms were correlated with lifespan variation. However, no obvious correlation was found with isoform position, homophilic binding strength, or expression level. Furthermore, phylogenetic analysis revealed that exons 4.4, 4.6, and 4.7 do not cluster within the same evolutionary branch. Interestingly, multiple sequence alignment of the 12 variable exon 4 isoforms revealed that exons 4.4, 4.6, and 4.7 shared a conserved amino acid region (S7F Fig), which may be associated with lifespan difference. Mutants containing a single exon 4 or two exon 4s differed significantly in adult lifespan, exhibiting a variable exon-specific manner (Figs 3G, 3H, S7D, and S7E). Furthermore, similar trends were observed in mutants containing a single exon 6, two exon 6s, a single exon 9, or two exon 9s (Figs 3G, 3H, S7D, and S7E). Taken together, it is clear from the study’s data that reducing Dscam1 diversity accelerates fly aging in an exon variant-specific manner.

### Fly fitness is highly sensitive to the reduction in Dscam1 diversity

**Fig 4:**
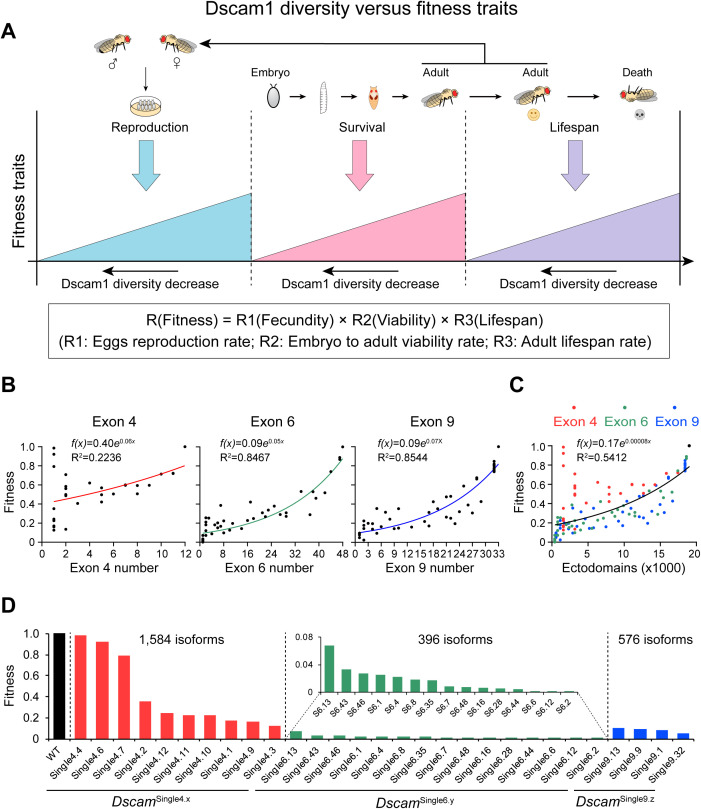
Reducing Dscam1 diversity affects fly fitness traits. **(A)** Schematic diagram summarizing the correlation of Dscam1 diversity with fitness traits of fly viability, fecundity, and lifespan. **(B)** Correlation analysis between the fitness of genotypes and the number of variable exons in variable exon 4, 6, or 9 clusters, respectively. The fitness traits of Dscam1 mutants are normalized to wild-type which was set to 1. **(C)** Correlation analysis between the fitness of mutants and the potential ectodomain diversity. **(D)** The comparison of the fitness between the wild-type and *Dscam*^Single4.x^, *Dscam*^Single6.y^, and *Dscam*^Single9.z^ mutants. The data underlying this figure can be found in S1 Data.

Although there are many different components of fitness, decisions surrounding an individual’s reproduction play a central role in determining its fitness [37,38]. Therefore, quantifying offspring and assessing parental contributions to them are considered essential for studying changes in individual fitness as well as for understanding species-specific population dynamics and life-history evolution [39]. As mentioned above, Dscam1 isoform diversity is necessary not only for fly fecundity but also for the survival and lifespan of animals (Fig 4A). High fecundity and survival rate are important for the propagation of fly offspring. In addition, high adult lifespan implies a longer reproductive period for egg production. As in previous studies [40], we used the number of offspring that successfully reached adulthood as a measure of fitness. To estimate the total number of eggs laid by the fly over its lifetime, we multiplied the daily egg production (Fecundity) by the number of days the fly survived (Lifespan). We then multiplied this value by the survival rate (Viability) from embryo to adult to determine the overall fitness of the fly. The overall reproductive capacity is based on an equation: R (fitness)= R1 × R2 × R3 (R1: egg production rate; R2: embryo-to-adult viability rate; R3: adult lifespan rate) (Fig 4A). The relative reproductive capacity of each mutant fly was then calculated in the study.

We found that *Dscam*^Δ4x^, *Dscam*^Δ6y^, and *Dscam*^Δ9z^ mutants exhibited reduced fly fitness. The relative fitness value increased substantially as the number of remaining variable exons 4, 6, and 9 increased (Fig 4B). In contrast to the sigmoidal or linear function in the other phenotypes, plotting relative fitness as a function of the number of remaining exon 4s, 6s, and 9s revealed exponential functions. Based on this correlation, relative fitness is very sensitive to small-scale reductions in exon 4s, 6s, and 9s. The relative fitness value of exon 4 mutants was less sensitive to diversity reduction compared with exon 6 or exon 9 mutants (Fig 4B), indicating a variable cluster-specific manner. A combination of these data from mutants with deletion of exons 4, 6, and 9 then revealed a strong positive correlation, corresponding to an exponential function (Fig 4C). In this scenario, overall fitness is highly sensitive to small-scale reductions in Dscam1 isoform diversity. Thus, Dscam1 isoform diversity may contribute significantly to the long-term survival of the species. These data suggest that fitness-related traits are the main drivers of the modern evolution of isoform diversity.

However, although the overall fitness rate was positively correlated with the number of available exons, mutants with the same degree of diversity exhibited considerable variation. For example, *Dscam*^Single4.x^ mutants encoding 1,584 isoforms varied strikingly in overall fitness, ranging from a maximum level comparable to the WT in *Dscam*^Single4.4^ to a minimum of less than one-eighth of the WT in *Dscam*^Single4.3^ (Fig 4D). This variation among individual isoforms partially reflects the specificity of the various isoforms in terms of fitness. These data indicate that the diversities of exons 4, 6, and 9 mediate fly fitness in a variable exon-specific manner.

### Dscam1 isoforms affect adult survival upon pathogen infection in diversity-correlated yet variable exon-specific manner

**Fig 5:**
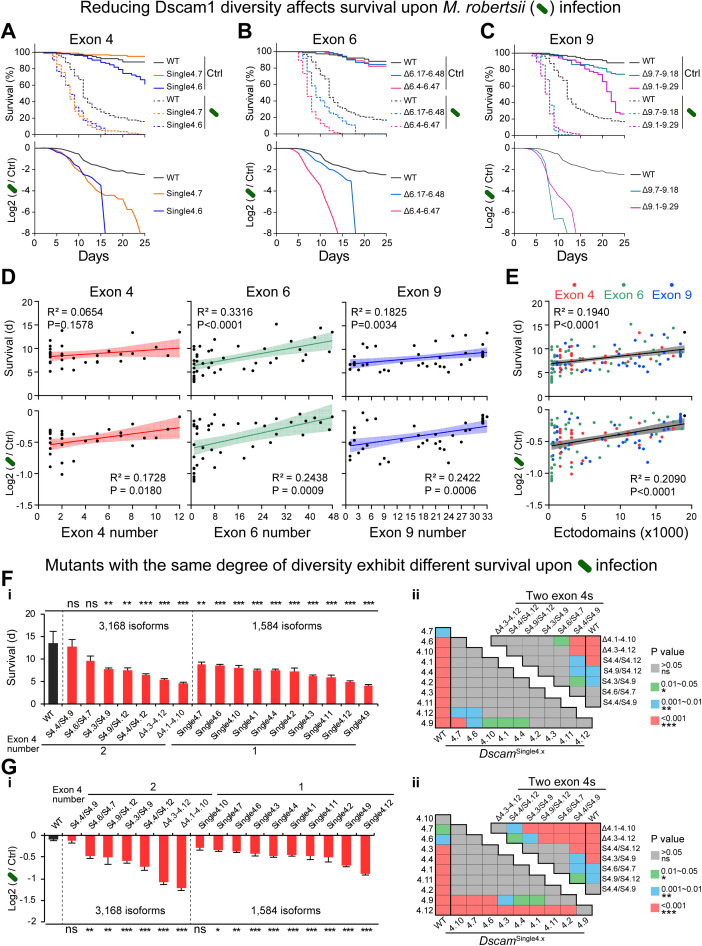
Reducing the Dscam1 diversity affects adult survival upon *Metarhizium robertsii* infection, also see S8 and S9 Figs. **(A–C)** The survival curves after infection upon Triton or *M. robertsii* for wild-type and exon 4 (A), exon 6 (B), and exon 9 (C) mutants are shown, respectively. The Triton groups (control) are represented by solid lines, while the *M. robertsii* groups are represented by dashed lines. **(D, E)** The mean survival days in the first 25 days of the infection group and the log2-transformed survival rates of *M. robertsii* infection group/blank group were linearly fitted to the number of variable exons 4, exon 6, exon 9 (D), or ectodomains (E), respectively. The 95% confidence intervals for the linear fit are shown with shading. **(F)** The comparison of the mean survival days in the first 25 days of the infection group between the wild type and the exon 4 mutants. Pairwise comparisons (one-way ANOVA with Tukey’s test) of were performed on the survival days of a single variable exon 4 or two variable exon 4 mutants, respectively. **(G)** The comparison of the mean log2 value between the wild type and the exon 4 mutants. Pairwise comparisons (one-way ANOVA with Tukey’s test) were performed on the mean log2 value of a single variable exon 4 or the two variable exon 4 mutants, respectively. ns, not significant; *P < 0.05; **P < 0.01; ***P < 0.001. The data underlying this figure can be found in S1 Data.

A large body of evidence suggests that Dscam1 plays an important role in immune defense against pathogens [41−45]. To dissect the contribution of Dscam1 isoform diversity in immune defense, two fungi, *Metarhizium robertsii* and *Beauveria bassiana*, which are widely used models in studying host-pathogen interactions in insects [46], were chosen. The survival rate of different deletion mutants upon infection with *M. robertsii* was first investigated. Compared with the WT control, almost all mutants with reduced exon 4, exon 6, or exon 9 diversity showed a lower survival rate upon *M. robertsii* infection (Figs 5A–5C, S8, and S9A). For example, only 12% of **Dsca*m*^Δ6.4-6.47^ mutants survived after 10 days of *M. robertsii* infection compared to 71% of WT, whereas there was no significant difference between the survival rate of *Dscam*^Δ6.4-6.47^ mutants and WT as control groups (Solid line, Fig 5B). This is reflected in the much lower log2 fold change (Infection/Control) of the survival rate in *Dscam*^Δ6.4-6.47^ mutants than in WT (Fig 5B). Consistently, most deletion mutants exhibited a much lower log2 fold change of the survival rate than WT (Figs 5A–5C, S8, and S9B). The adult survival rate substantially increased after infection as the number of remaining variable exons 4, 6, and 9 increased (Fig 5D, upper panel). A similar trend was observed when plotting the log2 fold change in the survival rate as a function of exon number (Fig 5D, lower panel). When the data for mutants deleting exons 4, 6, and 9 were combined, a strong linear correlation was revealed between survival rate and isoform diversity, as well as between the log2 fold change in survival rate and isoform diversity (Fig 5E). These data suggest that Dscam1 isoform diversity is largely correlated with fly resistance to pathogens.

However, the log2 fold change of the survival rate was found to be comparable to that of WT for several mutants, such as the *Dscam*^Single4.4/single4.9^, *Dscam*^∆6.10-6.20^, and *Dscam*^∆9.14-9.24^ mutants (S8C–S8E Fig). Moreover, mutants with the same degree of diversity exhibited obvious differences in the survival rate upon pathogen infection and the log2 fold change (Fig 5F and 5G). For example, exon 4 deletion mutants encoding 3,168 isoforms varied strikingly in terms of the survival rate upon pathogen infection (Fig 5F, panel i). A considerable amount of variation, albeit at a lower rate, was also observed in the *Dscam*^Single4.x^ mutants encoding 1,584 isoforms (Fig 5F, panel i). A similar trend was observed when we plotted using the log2 fold change in the survival rate (Fig 5G). These data indicate that Dscam1 isoforms mediate fly resistance in a variable exon-specific manner.

### Dscam1 isoforms mediate pathogen resistance in a pathogen-specific manner

**Fig 6:**
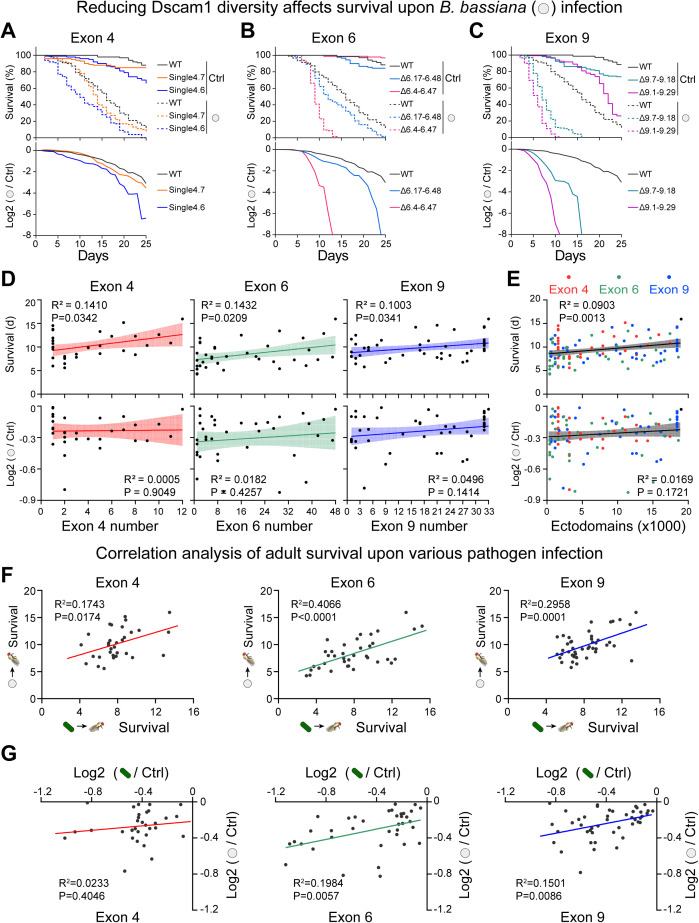
Reducing the Dscam1 diversity affects adult survival upon *Beauveria bassiana* infection, also see S10 and S11 Figs. **(A–C)** Survival curves after Triton or *B. bassiana* infection for wild-type and exon 4 (A), exon 6 (B), and exon 9 (C) mutants are shown, respectively. The Triton groups (control) are represented by solid lines, while the *B. bassiana* groups are represented by dashed lines. **(D, E)** The mean survival days in the first 25 days of the infection group and the log2-transformed survival rates of *B. bassiana* infection group/blank group were linearly fitted to the number of variable exons 4, exon 6, exon 9 (D) or ectodomains (E), respectively. The 95% confidence intervals for the linear fit are shown with shading. **(F)** Correlation analysis between the mean survival days in the first 25 days of exon 4, 6, or 9 mutants upon *Metarhizium robertsii* and *B. bassiana* infection, respectively. **(G)** Correlation analysis between the log2-transformed survival rates of infection group/blank group of exons 4, 6, or 9 mutants upon *M. robertsii* and *B. bassiana* infection, respectively. The data underlying this figure can be found in S1 Data.

To further investigate the specific role of Dscam1 isoforms in resistance to different pathogens, the effect of Dscam1 diversity on the survival rate after infection by *Beauveria bassiana*, another class of pathogen, was evaluated. Similar to *M. robertsii*, almost all mutants deleting exon 4s, exon 6s, or exon 9s showed lower survival rates upon *B. bassiana* infection (Figs 6A–6C, S10A, and S11A). The study also revealed that mutants with the same degree of diversity exhibited considerable variation in both the adult survival rate upon *B. bassiana* infection and in log2 fold change (S10C and S10D Fig). Notably, the mean survival and the log2 fold changes of *Dscam*^single4.4^ and *Dscam*^single4.7^ after *B. bassiana* infection were comparable to wild-type control, suggesting that the exons 4.4 and 4.7 play a more important role in the immune defenses (S10C and S10D Fig). These data suggest that isoform diversity contributes to fly resistance against *B. bassiana* in a variable exon-specific manner.

Similar to *M. robertsii*, the adult survival rate following *B. bassiana* infection decreased with the remaining number of exons 4, 6, or 9 decreased (Fig 6D, upper panel). However, the log2 fold change in the survival rate was not significantly correlated with the number of exon 4, 6, or 9 (Fig 6D, lower panel). There was a strong linear correlation between the survival rate and isoform diversity following *B. bassiana* infection (Fig 6E, upper panel)*,* but not between the log2 fold change in the survival rate and isoform diversity (Fig 6E, lower panel). These data indicate that Dscam1 isoforms have differential effects on fly resistance to *M. robertsii* and *B. bassiana.*

To further assess how Dscam1 isoforms affect different pathogens, the correlation between two pathogens, *M. robertsii* and *B. bassiana,* was analyzed. The survival rates in exon 4, exon 6, or exon 9 deletion mutants exhibited a significant correlation between *M. robertsii* and *B. bassiana*, respectively (Fig 6F). However, when plotting the log2 fold change in the survival rate, the exon 6 and exon 9 deletion mutants showed a significant correlation, while the exon 4 deletion mutants showed no significant correlation between *M. robertsii* and *B. bassiana* (Fig 6G)*.* These observations indicate that Dscam1 isoforms differentially act on *M. robertsii* and *B. bassiana*, suggesting that diverse isoforms may engage distinct pathways or targets of the pathogen. These data imply that Dscam1 isoforms share a common and specific defense role against various pathogens*.*

### Reducing the Dscam1 level reverts the fitness traits caused by the reduction in diversity

**Fig 7:**
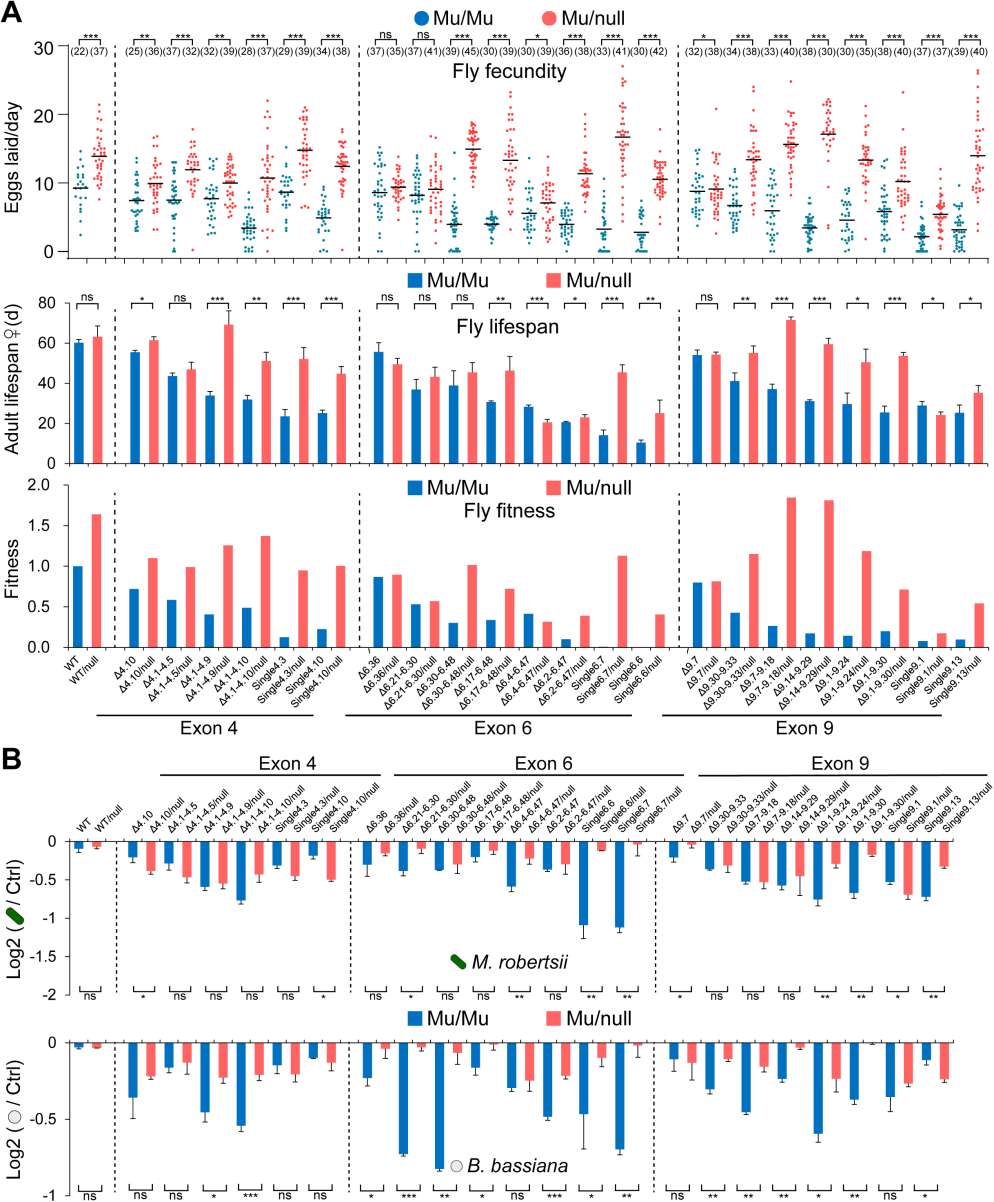
Reducing the Dscam1 level rescues the phenotype defect caused by reducing Dscam1 diversity, also see S12 Fig. **(A)** Comparison of the fly fecundity, the mean lifespan of adult flies, and fitness between the Dscam1 mutants carrying two copies (Mu/Mu) or one copy (Mu/null) of the mutant allele. The mutants of *Dscam*^Δ4.10^, *Dscam*^Δ4.1-4.5^, *Dscam*^Δ4.1-4.9^, *Dscam*^Δ4.1-4.10^, *Dscam*^Single4.3^, and *Dscam*^Single4.10^ in variable exon 4 cluster, *Dscam*^Δ6.36^, *Dscam*^Δ6.21-6.30^, *Dscam*^Δ6.30-6.48^, *Dscam*^Δ6.17-6.48^, *Dscam*^Δ6.4-6.47^, *Dscam*^Δ6.2-6.47^, *Dscam*^Single6.6^, and *Dscam*^Single6.7^ in variable exon 6 cluster, *Dscam*^Δ9.7^, *Dscam*^Δ9.30-9.33^, *Dscam*^Δ9.7-9.18^, *Dscam*^Δ9.14-9.24^, *Dscam*^Δ9.1-9.30^, *Dscam*^Single9.1^, and *Dscam*^Single9.13^ in variable exon 9 cluster were examined. Reducing the Dscam1 expression level partially rescued the fly fecundity, lifespan, and fitness traits in homozygous mutants. **(B)** The comparisons of the log2-transformed survival rates of the infection group/blank group between the Dscam1 mutants with one copy or two copies of the mutant allele upon *Metarhizium robertsii* and *Beauveria bassiana* infection, respectively. Reducing the Dscam1 expression level partially rescued the survival rates upon *M. robertsii* and *B. bassiana* infection in homozygous mutants. ns, not significant; *P < 0.05; **P < 0.01; ***P < 0.001 (Student *t* test, two-tailed). The data underlying this figure can be found in S1 Data.

Our previous studies have shown that reducing the Dscam1 level reverses the neural phenotypes due to reduced diversity [23,32]. To further assess the effect of reduced Dscam1 levels on multiple aspects of fly fitness, a comparison was made between **Dsca*m*^Δ4/6/9^/*Dscam*^null^ flies bearing one copy of a mutant allele. The study revealed that *Dscam*^Δ4/6/9^/*Dscam*^null^ flies had significantly increased fecundity rates compared to homozygous *Dscam*^Δ4/6/9^ mutants (Fig 7A). These data suggest that reducing Dscam1 levels by half remarkably restores fly fecundity due to reduced diversity, independent of variable exon cluster. Consistent with previous studies [23,32], reducing Dscam1 levels partially rescued the fly viability defects caused by reduced diversity (S12A Fig). Furthermore, a less steep downward trend in fly lifespan was observed with the reduction of the remaining exon 4s, exon 6s, or exon 9s in *Dscam*^Δ4/6/9^/*D*scam**^null^ flies (Figs 7A and S12B). As a result, *Dscam*^Δ4/6/9^/*Dsca*m**^null^ flies exhibited more fitness than homozygous **Dsca*m*^Δ4/6/9^ mutants (Fig 7A). Collectively, these data indicate that reducing Dscam1 levels by half can partially rescue the overall fitness defects caused by reduced diversity.

We next investigated the effect of reducing Dscam1 levels on adult survival upon pathogen infection. It was discovered that most exon 4, exon 6, or exon 9 mutants with one copy of Dscam1 exhibited higher survival rates upon *M. robertsii* infection than homozygous **Dsca*m*^Δ4/6/9^ mutants (Figs 7B and S12C). A similar trend was observed for *B. bassiana* infection (Figs 7B and S12D). These data demonstrate that reducing Dscam1 levels by half can partially rescue the immunity-related defects caused by reduced diversity. Taken together, our results indicate that fitness traits and immunity are not only mediated by the number of Dscam1 isoforms but also related to Dscam1 protein levels and isoform composition.

## Discussion

Alternative splicing of *Drosophila melanogaster Dscam1* potentially generates 38,016 different receptor isoforms [1]. Genetic studies have shown that Dscam1 is functionally essential in both nervous and immune systems [1,11–17,41–45]. Our recent work demonstrated that 2,000 isoforms are sufficient to sustain normal neuronal self/non-self discrimination in dendritic arborization (da) neurons [23]. In this study, we assess multiple aspects of fly fitness and immunity in deletion mutants reducing ectodomain isoform diversity from 18,612 to 396. These phenotype–diversity correlation analyses from this study, together with our previous study [23,32], revealed that fitness-related traits were much more sensitive to reductions in Dscam1 diversity than canonical neuronal self/non-self discrimination. Thus, fitness and immunity properties rather than canonical neuronal self/non-self discrimination functions might be the dominant drivers during the modern diversification of the Dscam1 isoform.

### Modern Dscam1 diversification is driven by fitness and immunity

**Fig 8:**
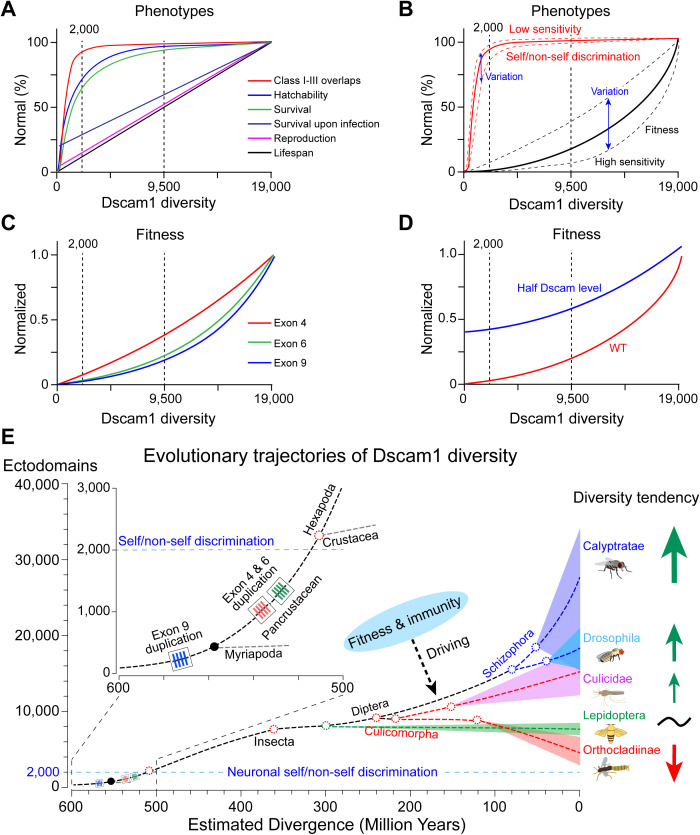
Fitness and immunity drive the modern expansion of Dscam1 diversity. **(A)** Summary of correlation analysis for different phenotypes resulting from reduced Dscam1 diversity. Class I-III overlap phenotype–diversity correlation was from our previous studies [23,32]. Unexpectedly, correlation analysis revealed that fitness-related traits and immunity properties are much more sensitive to the reduction of Dscam1 diversity than nervous traits. **(B)** Comparison of the fitness and the canonical neuronal self/non-self discrimination in the sensitivity of the Dscam1 diversity. Phenotype–diversity correlation analysis from our previous study revealed that 2,000 isoforms are sufficient to sustain normal neuronal self/non-self discrimination in dendritic arborization (da) neurons [23]. Thus, we conclude that the role of Dscam1 isoforms in canonical neuronal self-avoidance is mediated by a small fraction of all isoforms (<1/10), whereas a separate role essential for other development contexts, likely in fitness, requires more isoform diversity. **(C)** A comparison of the fitness-diversity correlations in *Dscam1* exon 4, 6, and 9 clusters showed a cluster-specific manner. **(D)** Reducing the Dscam1 level affected the fitness-diversity correlations. **(E)** Proposed evolutionary trajectories of Dscam1 in arthropod species. Exon 9 duplication originated before the divergence of Myriapoda and Pancrustacea, while variable exon 4 and 6 duplications emerged in the Pancrustacea ancestor. ~2,000 Dscam1 isoforms are sufficient to maintain neuronal self/non-self discrimination. During early pancrustacean evolution, it is likely that neuronal self/non-self discrimination might drive the duplication of exons 4, 6, and 9 of ancestral Dscam1 to attain 2,000 isoforms. When Dscam1 isoforms exceeded 2,000, neuronal self/non-self discrimination was no longer the main driver for Dscam1 isoform expansion. Instead, our data indicate that productivity-related traits and immunity are possibly the main drivers for the modern evolution of isoform diversity.

Genetic studies have revealed that Dscam1 isoform diversity is differentially required in a variety of developmental contexts. The sensitivity to genetic manipulations of Dscam1 isoform diversity varies remarkably across traits. By comparing the correlations between Dscam1 isoform diversity and phenotype defects, this study found that productivity-related traits were most sensitive to reductions in Dscam1 isoform diversity and tended to be associated with specific exon clusters or isoforms (Fig 8A–8C). By contrast, our previous study indicated that neuronal self/non-self discrimination was insensitive to Dscam1 diversity when it exceeded 2,000 isoforms and was independent of exon clusters or isoforms, suggesting that 2,000 isoforms are sufficient to sustain normal neuronal self/non-self discrimination [23]. Furthermore, reducing the Dscam1 level can restore the fitness traits due to reduced diversity, similar to neural phenotypes (Fig 8D) [23,32].

Based on the phylogenetic analysis of Arthropoda *Dscam* genes from 178 species (S1 Table), the evolutionary trajectories of Dscam1 diversity are proposed and graphically illustrated with five representative insect clades (Fig 8E). The internal tandem duplication in exon 9 originated in early arthropod evolution, whereas tandem duplication in exons 4 and 6 emerged after the divergence of pancrustacean and Myriapoda [47]. This extraordinary genomic architecture resulting from pancrustacean Dscam1 is reminiscent of that of the immunoglobulin and T-cell receptor genetic loci in vertebrates. During early pancrustacean evolution, neuronal self/non-self discrimination may have driven duplications of exons 4, 6, and 9 of ancestral Dscam to attain 2,000 isoforms. When Dscam isoforms exceed 2,000, neuronal self/non-self discrimination is no longer the main driver for Dscam isoform expansion. Instead, our data suggest that productivity-related traits may be the main drivers for the evolution of the isoform diversity. The bursting expansion in ectodomains of the housefly Dscam1 may be compatible with its powerful fecundity. Conversely, the Antarctic midge encodes only about 3,000 ectodomains [31], which may be compatible with its low fecundity. Alternatively, this bursting expansion of splice isoforms in houseflies may be driven by selection acting on immune function, which enables continuous and rapid adaptation to a pathogen-rich environment. In contrast, the striking compression of the Dscam1 splice repertoire in midges may be compatible with life in a pathogen-poor environment. Based on these correlation analyses with the mutational data presented here and previous studies [42], we suggest that taxon-specific Dscam1 isoforms enable organisms to adapt to changing conditions and stresses.

### Dscam1 isoforms have shared and specific roles in immune defense

Our present study demonstrates that the effect of Dscam1 isoforms shows some correlation between *M. robertsii* and *B. bassiana* (Fig 6F and 6G)*.* On the other hand, Dscam1 isoforms differentially affected the fly resistance to *M. robertsii* and *B. bassiana* in a variable exon-specific manner (Fig 6). These results imply that fly Dscam1 isoforms have specific resistance to different pathogens*.* This notion is also supported by previous studies on insect and crustacean Dscams [42]. For example, the mosquito (*Anopheles gambiae*) Dscam responds to infection by producing pathogen challenge-specific isoform [45]. The rEsDscam isoform (exons 4.24, 6.19) from the Chinese mitten crab (*Eriocheir sinensis*) may bind specifically to *Staphylococcus aureus* and enhance its phagocytosis by approximately 100%, whereas the rEsDscam isoform (exons 4.12, 6.20) may bind specifically to *V. parahemolyticus* and enhance its phagocytosis by approximately 70% [42]. After the challenge with pathogens, the *Litopenaeus vannamei* Dscam (LvDscam) induced the expression of certain Ig2 + Ig3 combinations [44]. Collectively, our present data, together with previous studies, strongly suggest that Dscam1 isoforms have shared and specific defense roles against various pathogens*.*

It remains unclear how Dscam1 isoforms differentially affect immune function against different pathogens. Several possible mechanisms were considered. The first mechanism possibly involves ligand–receptor heterophilic interactions. Analogous to antigen-specific receptors in the adaptive immune system of higher vertebrates, the Dscam receptors are proposed to act as ‘self’ molecules that interact with ‘non-self’ molecules (such as pathogens) in the invertebrate immune system. In the nervous system, Dscam receptors may recognize distinct ligands, such as netrin-1 [48–50] and Slit-N [51,52]. Protein-interaction studies have shown that the ectodomain of Dscam binds to a disintegrin and metalloprotease domain 10 (ADAM10), which is involved in the immune defense against bacterial infection [53]. This possibility is also supported by our observation that Dscam1 isoforms act in a variable exon-specific manner. As the reduction in Dscam1 diversity disproportionately leads to defects, Dscam1 isoforms may mediate immune defense against pathogens through ligand–receptor Dscam signaling. It is interesting to assess the reduced antifungal immunity in Dscam1 mutants through detecting the expression of downstream antifungal peptide genes, such as *Drosomycin* [54]. Second, it possibly involves Dscam-mediated hematopoiesis in innate immunity and phagocytosis of pathogens. Dscam1 regulates fly hematopoiesis in a cell-autonomous manner [55]. Third, our studies indicate that Dscam1 isoform diversity is linked to neuronal wiring, fly fecundity, lifespan, and immune responses. It is reasonable to speculate that Dscam1-mediated neuronal defects might be detrimental enough to affect the issues of fitness-related traits and immunity in flies. Finally, since Dscam1 plays a dual role in both neuronal and immune functions, neuro-immunological feedback may be crucial in linking its roles in these systems. Recent studies have indicated that Dscam1 has non-autonomous effects on hemocyte survival in *D. melanogaster* through its expression in sensory neurons [55]. It will be interesting to explore the potential neuro-immunological feedback mechanisms involving Dscam1 in the future, which may shed light on its immune functions.

### Functional link between Dscam1 expression levels and isoform diversity

This study showed that reducing the Dscam1 expression level in most *Dscam*^Δ4/6/9^ flies remarkably decreased the fitness-related traits and survival upon pathogen infection defects caused by reduced Dscam1 diversity. Mechanistically, *Dscam*^Δ4/6/9^ mutants expressed a significantly less diverse set of isoforms, leading to excessive homophilic binding strength. This over-strong signal in these mutants would be weakened by reducing the overall Dscam1 expression level. The second possible mechanism involves other potential functions of single isoforms. Several studies have indicated that Dscam receptors recognize distinct and specific ligands [48–50]. This possibility is partially supported by our observation that reducing overall Dscam1 expression levels modulated fitness-related traits and survival upon pathogen infection defects in a variant-related manner. As the Dscam1 protein could initiate signaling underlying multiple functions via homophilic and heterophilic interactions [7,51,52,56], Dscam1 expression levels are functionally linked to isoform diversity via combinatorial mechanisms.

The resulting over-strong Dscam1 signal likely leads to developmental defects, as reported in previous studies [32,57,58]. By contrast, reducing Dscam1 levels prevents excessive Dscam1 signaling, thus rescuing fitness-related traits and survival upon pathogen infection defects. Excessive signaling is detrimental to fitness-related traits, which is relieved by the weakened signaling induced by reducing Dscam1 levels and/or increasing diversity. In this sense, Dscam1 isoform diversity produced by alternative splicing buffers Dscam1 signaling to ensure normal fitness. These data suggest a functional and mechanistic link between Dscam1 levels and isoform diversity independent of isoform specificity. It will be interesting to determine whether this expression level-diversity link exists in other genes.

### Dscam Diversification: An evolutionary driver in arthropod speciation?

It is well acknowledged that reproductive capacity and immunity are key factors influencing the long-term survival of a species. Our data indicate that reductions in Dscam1 diversity significantly reduce fecundity, viability, lifespan, and immune defense. This functional diversity landscape provides a wider range of trait sensitivity, which is often closely associated with fly adaptation. This suggests that, on a larger evolutionary scale, the evolution of Dscam1 isoform diversity can contribute to lineage divergence and speciation in arthropods. From a mechanistic standpoint, the number of Dscam1 variable exons can be expanded and condensed by staggered homologous recombination [30]. Once environmental conditions change, isoform change enables organisms to adapt to changing conditions. These adaptation events harboring Dscam1 occur via different pathways. One is that Dscam1 isoforms affect reproductive capacity. Our data in this study show that reducing the Dscam1 diversity leads to reduced fly fecundity, survival rate, and shorter lifespan. It would be interesting to explore the molecular mechanism by which Dscam1 isoforms mediate fly fecundity and growth in the future. The other pathway is Dscam1 isoform-mediated immune defense. Our findings suggest that Dscam1 diversity may act as a driver for lineage divergence and speciation in arthropods.

In summary, all fitness-related traits generally improved as the potential number of isoforms increased; however, the magnitude of the changes varied remarkably in a variable cluster-specific manner. Correlation analysis revealed that fitness-related traits were much more sensitive to reductions in Dscam1 diversity compared to canonical neuronal self/non-self discrimination. These data indicate that the role of Dscam1 isoforms is essential for certain developmental contexts and resistances, likely in fitness and immunity, requires more isoform diversity. Thus, we show that fitness and immunity properties, rather than canonical neuronal functions, are the dominant drivers during the modern diversification of the Dscam1 isoform.

## Materials and methods

### Materials

The *{nos-Cas9}attP2* fly was used to introduce Dscam1 mutations via the CRISPR/Cas9 technology. *W*^*1118*^ served as the wild type control, while *if/Cyo* or *if/Cyo.GFP* acted as a balancer for the mutant screen. The **Dsca*m*^*21*^ line was generously provided by the laboratory of Dr. Jianhua Huang, Zhejiang University, China. *Dscam*^∆4.3-4.6^ and *Dscam*^∆4.4-4.12^ as gifts from previous studies [33]. In addition, we received *M. robertsii* and *B. bassiana* as gifts from the laboratory of Dr. Weiguo Fang, also at Zhejiang University, China.

### Annotation of the *Dscam1* gene

The genomic sequences of the *Dscam1* gene in pancrustacean species were obtained by comparative genome analysis from the National Center for Biotechnology Information (NCBI) (https://blast.ncbi.nlm.nih.gov/Blast.cgi) (S1 Table). The number of variable exons was confirmed by homology alignment. The evolutionary classification of each species and its current measured genome size were checked in NCBI.

### Generation of Dscam1 mutant flies

The CRISPR/Cas9 system, in conjunction with non-homologous end joining, was used to generate variable exon deletions in Dscam1 [59]. Two sgRNAs were co-injected into *{nos-Cas9}attP2* embryos by UniHuaii CO., China. The mosaic flies were crossed with the balancer. Female offspring were collected and analyzed by PCR. Male offspring from the mutation-positive tubes were then crossed with the balancer stock once more. Finally, genomic DNA was extracted from each male fly and the mutations were verified by PCR to establish the final mutant lines. The mutation sequences for all Dscam1 variable exon deletion mutants are listed in S2 Table, and the primers used for sgRNA and mutant screening are provided in S3 Table.

### RT-PCR detection

The total RNA of head tissues of Dscam1 mutants and wild type was obtained by Trizol (Invitrogen). The complementary DNA (cDNA) was obtained via performing reverse transcription by the SuperScript III system (Invitrogen) using the specific primers on *Dscam1* constitutive exon 10. The cDNA was amplified by PrimeSTAR DNA Polymerase (TaKaRa) using the specific primers on both sides of the variable exon 4, 6, or 9 clusters, respectively. The amplified products were detected via agarose gel electrophoresis.

### Quantitative real-time PCR

The total RNA from the ovary tissues of Dscam1 mutants and wild-type flies was extracted using Trizol (Invitrogen) according to the manufacturer’s protocol. The cDNA was synthesized through reverse transcription by HiScript II 1st Strand cDNA Synthesis Kit (Vazyme Biotech Co., China). The cDNA was diluted 10 times and then used for quantitative PCR with the HiScript III RT SuperMix for qPCR (Vazyme Biotech Co., China). The reactions were performed using the Bioer 9600 FQD-96A System. Beta-actin was used as an internal control. Specific primers used in this study were summarized in S3 Table.

### RNA extraction, library construction, and sequencing

RNA from total samples was isolated and purified using Trizol (ThermoFisher, 15596018) according to the manufacturer’s protocol. mRNA containing PolyA (poly-adenylated) sequences was specifically captured through two rounds of purification using oligo(dT) magnetic beads (Cat. 25-61005, ThermoFisher, USA). Next, cDNA was synthesized from the fragmented RNA using the Invitrogen SuperScript II Reverse Transcriptase. For the second strand synthesis, *Escherichia coli* DNA polymerase I (NEB, cat. M0209, USA) and RNase H (NEB, cat. M0297, USA) were used, resulting in the conversion of RNA-DNA hybrid duplexes into double-stranded DNA. During this process, dUTP Solution (ThermoFisher, cat. R0133, CA, USA) was incorporated into the double-stranded DNA, and the ends were filled in to form blunt ends. An ‘A’ base was added to both ends to enable ligation with adapters containing a ‘T’ base. The fragments were then selected and purified based on size using magnetic beads. After the size selection and purification, PCR amplification was performed to create a library with fragment sizes of approximately 300 bp ± 50 bp. Finally, paired-end sequencing was carried out using the Illumina NovaSeq (LC Bio Technology CO., Hangzhou, China) according to standard operating procedures. The raw reads were filtered using Cutadapt (version: 1.9), and the quality of the clean reads, as well as the Q20, Q30, and GC content, was assessed using FastQC (version: 0.11.9). The expression levels of all transcripts were estimated using StringTie (version: 2.1.6) and ballgown. Differential gene expression between the two groups was analyzed using DESeq2. Subsequently, GO term enrichment analysis was performed on the differentially expressed genes. The raw RNA-seq data generated in this study have been submitted to the Sequence Read Archive (SRA) database and are available under accession numbers PRJNA1246336.

### Western blot analysis

The head tissues of Dscam1 mutants and wild-type flies were obtained. A strong RIPA lysis buffer (CW-BIO, Jiangsu, China) and the PMSF (Phenylmethylsulfonyl fluoride) protease inhibitor (Beyotime, Shanghai, China) were used to deal with the samples. Standard western blot protocol (Abcam, Cambridge, UK) was performed using the polyacrylamide gel electrophoresis (PAGE). The primary antibodies to Dscam1 (ab43847, diluted 1:5000), β-actin (ab8227, diluted 1:10,000), and secondary antibodies (Goat Anti-Rabbit IgG, 1:10,000, CW-BIO) were used. The immunoreactive bands of Dscam1 and β-actin were detected using the eECL Western Blot Kit (CW-BIO). A chemiluminescence imager (Tanon 5200) was used in this process.

### Fecundity assay

Fly fecundity was measured in a similar manner to the previous study [60]. Carefully collect approximately 40 unmated female and male fruit flies, respectively. Individual males and females were then placed into separate tubes of fresh food. After 24 h, transfer the flies to new food tubes and carefully count the number of embryos in the original tubes. This process was repeated daily until day 6, and the average daily egg production per female from days 2 to 6 was calculated.

### Ovarian and testicular morphology detection

Three- to six-day-old female or male flies were dissected in phosphate-buffered saline (PBS) to obtain ovaries and testes. The ovarian and testicular morphology was photographed under the microscope (Nikon SMZ18), and the size of the ovaries and testicular was counted using software (Nikon NIS-Elements D 4.60.00).

### Survival rate detection

Survival rates refer to the percentage of embryos that successfully develop into adult flies. This developmental process comprises four stages. Firstly, the hatching rate is determined, measuring the number of hatched embryos out of approximately 200 newly laid eggs after 48 h at 25 °C. Next, the pupation rate is assessed, involving 90 larvae collected in food tubes and counted the pupae after 3–4 days. Finally, the eclosion rate is evaluated, gauging the number of adult flies emerging from pupae after 4–5 days in the food tubes. The overall survival rate is calculated by combining the hatching rate, pupation rate, and eclosion rate. To ensure reliability, three biological replicates were conducted.

### Lifespan analysis

Lifespan testing for each genotype was performed similarly to the previous study [61]. Collect 150 male and female adult flies that have eclosed within one day and mix them together for 2 days of adaptation. After acclimatization, male and female flies were separated and distributed into 5 food tubes, 30 in each tube. The number of dead flies was counted every day, and food tubes were replaced every day until all flies were dead. The total number of days from the eclosed of 30 fruit flies in each tube to their death was divided by 30 to obtain the average survival days for that group. The average survival days for each of the five groups were calculated separately, and the average of these five groups represented their average lifespan. Finally, the lifespan of each genotype was calculated. All flies were maintained on standard cornmeal medium in a constant temperature incubator at 25 °C.

### Fly pathogen infection

As in the previous study [62], this paper uses *M. robertsii* and *B. bassiana* to infect flies, respectively. Approximately 120 newly eclosed adult flies within 2 days were collected and raised at 25 °C for 1 day to allow them to mate freely and wait for infection. Add 1 mL of 0.01% Triton to a 2 mL centrifuge tube, scrape the spores of *M. robertsii* or *B. bassiana* that have been cultured for 14–20 days, and place them in the centrifuge tube. Filter the mycelium with a filter element to obtain the spore stock solution. The spore stock solution was diluted 40 times with 0.01% Triton, the spore concentration was calculated with a hemocytometer, and the spore suspension was then diluted to the required concentration. Pick female flies, each genotype is divided into a control group and a treatment group. Considering that the rapid mortality of the mutants in our study obscured the differences among a large number of Dscam1 mutants with varying isoform numbers, we used the lower concentrations of the conidial suspensions and lower humidity conditions (~70%) than those in previous studies [63,64]. The flies in the control group were treated with 15 mL of 0.01% Triton solution, vortexed for 15 s, and the flies in the treatment group were treated with 15 mL of 1 × 10^5^ *M. robertsii* or 1 × 10^6^ *B. bassiana* spore suspension at the same strength. After the fruit flies woke up and their wings were fully opened, the control group and the treatment group of each fruit fly were divided into three food tubes and placed in a 25 °C incubator for culture. Counting was started after 48 h, the food tube was changed every 24 h, and the number of dead fruit flies was counted until all the flies died.

### Statistical analysis

All raw data of each genotype used to generate the graphs are in S1 Data. The correlation analysis between the fitness traits and Dscam1 ectodomain diversity was conducted using GraphPad Prism version 8.0.2 for Windows. Two-tailed Student *t* tests were used to compare the two groups. One-way ANOVA with Dunnett’s test was used to compare multiple experimental groups and the control group. In addition, one-way ANOVA with Tukey’s test was used for pairwise comparison between multiple groups. *P* > 0.05 considered not significant (ns), *P < 0.05, **P < 0.01, and ***P < 0.001.

## Supporting information

S1 Fig Dscam1 isoform diversity in Dipteran species, Related to Fig 1. A phylogenetic tree of Dipteran species is shown on the left. The number of exons in variable exon 4, 6, and 9 cluster, and ectodomains diversity are shown in a different color on the right. Species with extreme Dscam1 isoform numbers are shown in red font. The data underlying this figure can be found in S1 Data.
(TIF)

S2 Fig Design and characterization of *Dscam1* mutant flies, Related to Fig 2. **(A)** Schematic diagram of the construction of Dscam1 variable exon deletion mutants. **(B)** RT-PCR diagram from the head tissues of wild-type and *Dscam1* mutants. No obvious variable exon skipping or abnormal splicing was detected in *Dscam1* mutants. **(C)** The protein levels in the head tissues of the *Dscam1* mutants were similar to the wild-type controls. **(D)** Survival rates of the *Dscam1* mutants and the wild-type control are shown. ns, not significant; *P < 0.05; **P < 0.01; ***P < 0.001 (one-way ANOVA with Dunnett’s test). The data underlying this figure can be found in S1 Data.
(TIF)

S3 Fig The effect of female or male Dscam1 mutants on fecundity, Related to Fig 2. **(A–C)** Deletion of the Dscam1 isoform significantly reduced the fecundity of the mutant compared with the wild type. To explore the contribution of different sexes of Dscam1 mutant flies to reduced fecundity, female and male Dscam1 mutant flies were separately crossed with wild-type controls, and the fertility of the cross combinations was assessed. ns, not significant; *P < 0.05; **P < 0.01; ***P < 0.001 (one-way ANOVA with Dunnett’s test). The data underlying this figure can be found in S1 Data.
(TIF)

S4 Fig Reducing Dscam1 diversity affects the ovary development in female flies, Related to Fig 2. **(A)** Representative pictures of ovaries from Dscam1 mutants and the wild type. Dscam1 mutants showed small ovaries compared with the wild-type control. Scale bars, 300 µm. **(B)** Quantitative analysis of the ovary lengths of Dscam1 mutants compared with wild type. The fecundity was positively correlated with the ovary length. **(C)** Quantitative analysis of the ovary area of Dscam1 mutants compared with the wild type. The fecundity was positively correlated with the ovary area. Numbers in parentheses refer to the number of ovaries of each genotype studied. ns, not significant; *P < 0.05; **P < 0.01; ***P < 0.001 (one-way ANOVA with Dunnett’s test). The data underlying this figure can be found in S1 Data.
(TIF)

S5 Fig Reducing Dscam1 diversity affects the testis development in male flies, Related to Fig 2. **(A)** Representative pictures of testis from Dscam1 mutants and the wild type. Scale bars, 100 µm. **(B)** Quantitative analysis of the diameter of the testis cross section of Dscam1 mutants compared with wild type. Numbers in parentheses refer to the number of testes of each genotype studied. ns, not significant; *P < 0.05; **P < 0.01 (one-way ANOVA with Dunnett’s test). **(C)** RNA-seq of ovarian and testicular tissues of *Dscam*^Single4.3^ mutants and wild type. GO enrichment analysis of differentially expressed genes revealed two reproduction-related terms: sexual reproduction and multicellular organism reproduction. **(D)** RT-qPCR validation of differentially expressed genes in these two terms. *P < 0.05; **P < 0.01 (Student *t* test, two-tailed). The data underlying this figure can be found in S1 Data.
(TIF)

S6 Fig Reducing Dscam1 diversity affects adult lifespan, Related to Fig 3. **(A–C)** Mean female and male adult lifespan of wild-type and Dscam1 mutants with deletion of variable exon 4 (A), exon 6 (B), and exon 9 (C) were calculated, respectively. The number of remaining variable exons of each mutant is shown at the bottom. *P < 0.05; **P < 0.01; ***P < 0.001; ns, not significant (one-way ANOVA with Dunnett’s test). The data underlying this figure can be found in S1 Data.
(TIF)

S7 Fig Reducing Dscam1 diversity decreases adult lifespan in an exon-specific manner, Related to Fig 3. **(A)** Correlation analysis between the mean male lifespan and the mean female lifespan in exon 4, exon 6, and exon 9 genotypes, respectively. **(B)** Correlation analysis between the mean male lifespan and the mean female lifespan in Dscam1 mutants. Different variable clusters of mutants are shown as dots of different colors. **(C)** The comparison of the mean male lifespan between the wild-type and *Dscam*^Single4.x,^ *Dscam*^Single6.y^, and *Dscam*^Single9.z^ mutants. ns, not significant; ***P < 0.001 (one-way ANOVA with Dunnett’s test). **(D, E)** Pairwise comparisons (one-way ANOVA with Tukey’s test) were performed on the lifespan of a single variable exon mutants (D) or the two variable exon mutants (E), respectively. (F) Multiple sequence alignment of the 12 variable exon 4s showed that exons 4.4, 4.6, and 4.7 share a conserved amino acid region. The circles on the right represent the proportional changes in the lifespan of the corresponding *Dscam*^Single4.x^ mutants. Due to abnormal splicing in *Dscam*^Single4.5^ and *Dscam*^Single4.8^ mutants [32], the symbol “*” indicates the absence of lifespan data for these two mutants. The data underlying this figure can be found in S1 Data.
(TIF)

S8 Fig Reducing the Dscam1 diversity affects adult survival upon *Metarhizium robertsii* infection, Related to Fig 5. **(A)** The survival curves of exon 4, exon 6, and exon 9 mutant flies infected by Triton and *M. robertsii* are shown, respectively. The remaining variable exon numbers of the mutants are shown on the right. **(B)** The log2-transformed survival curves of *M. robertsii* infection group/blank group for Dscam1 exon 4, 6, and 9 mutants are shown, respectively. The remaining variable exon numbers of the mutants are shown on the right. **(C–E)** The survival curves and the log2-transformed survival curves upon *M. robertsii* infection for wild-type and exon 4 (*Dscam*^Single4.4/single4.9^) (C), exon 6 (*Dscam*^∆6.10-6.20^) (D), and exon 9 (*Dscam*^∆9.14-9.24^) (E) mutants are shown, the log2 fold change of the survival rate in these mutants was comparable with the WT control. The data underlying this figure can be found in S1 Data.
(TIF)

S9 Fig Reducing the Dscam1 diversity affects adult survival upon *Metarhizium robertsii* infection, Related to Fig 5. **(A)** The comparison of the mean survival days in the first 25 days of the infection group between the wild type and the Dscam1 mutants. **(B)** The mean survival days in the first 10 days of the infection group and the blank group were calculated, and the log2 value was obtained. The comparison of the mean log2 value between the wild type and the Dscam1 mutants. *P < 0.05; **P < 0.01; ***P < 0.001; ns, not significant (one-way ANOVA with Dunnett’s test). The data underlying this figure can be found in S1 Data.
(TIF)

S10 Fig Reducing the Dscam1 diversity affects adult survival upon *Beauveria bassiana* infection, Related to Fig 6. **(A)** The survival curves of exon 4, exon 6, and exon 9 mutant flies infected by Triton and *B. bassiana* are shown, respectively. The remaining variable exon numbers of the mutants are shown on the right. **(B)** The log2-transformed survival curves of *B. bassiana* infection group/blank group for Dscam1 exon 4, 6, and 9 mutants are shown, respectively. The remaining variable exon numbers of the mutants are shown on the right. **(C)** The comparison of the mean survival days in the first 25 days of the infection group between the wild type and the exon 4 mutants. Pairwise comparisons (one-way ANOVA with Tukey’s test) were performed on the survival days of the remaining single variable exon 4 or the remaining two variable exon 4 mutants, respectively. **(D)** The comparison of the mean log2 value in the first 10 days between the wild type and the exon 4 mutants. Pairwise comparisons (one-way ANOVA with Tukey’s test) were performed on the mean log2 value of a single variable exon 4 or the two variable exon 4 mutants, respectively. ns, not significant; *P < 0.05; **P < 0.01; ***P < 0.001. The data underlying this figure can be found in S1 Data.
(TIF)

S11 Fig Reducing the Dscam1 diversity affects adult survival upon *Beauveria bassiana* infection, Related to Fig 6. (A) The comparison of the mean survival days in the first 25 days of the infection group between the wild type and the Dscam1 mutants. **(B)** The mean survival days in the first 10 days of the infection group and the blank group were calculated, and the log2 value was obtained. The comparison of the mean log2 value between the wild type and the Dscam1 mutants. *P < 0.05; **P < 0.01; ***P < 0.001; ns, not significant (one-way ANOVA with Dunnett’s test). The data underlying this figure can be found in S1 Data.
(TIF)

S12 Fig Reducing the Dscam1 level rescues the phenotype defect caused by reducing Dscam1 diversity, Related to Fig 7. **(A)** The mean survival rates of adult flies of Dscam1 mutants with one copy or two copies of the mutant allele are shown. Reducing the Dscam1 expression level partially rescued the fly viability in homozygous mutants. **(B)** The mean lifespan of adult flies of Dscam1 mutants with one copy or two copies of the mutant allele is shown. **(C)** The log2-transformed survival rates and the mean survival days in the first 25 days of the *Metarhizium robertsii* infection of Dscam1 mutants with one copy or two copies of the mutant allele are shown. Reducing the Dscam1 expression level partially rescued the fly survival upon *M. robertsii* pathogen infection. **(D)** The log2-transformed survival rates and the mean survival days in the first 25 days of the *Beauveria bassiana* infection of Dscam1 mutants with one copy or two copies of the mutant allele are shown. *P < 0.05; **P < 0.01; ***P < 0.001; ns, not significant (Student *t* test, two-tailed). The data underlying this figure can be found in S1 Data.
(TIF)

S1 Table Summary of Pancrustacea species used in this study. (PDF)

S2 Table Summary of the mutation sequences of *Dscam1* mutants. (PDF)

S3 Table Summary of specific primers used for sgRNA, mutant screening, and RT-PCR. (PDF)

S1 Raw Images Uncropped blots and minimally adjusted images for S2B and S2C Fig. (PDF)

S1 Data Data that underlie Figs 1B–1E, 2C–2I, 3A–3H, 4B–4D, 5A–5G, 6A–6G, 7A, 7B, S1, S2D, S3A–S3C, S4B, S4C, S5B–S5D, S6A–S6C, S7A–S7E, S8A–S8E, S9A, S9B, S10A–S10D, S11A, S11B, and S12A–S12D. (XLSX)

## Abbreviations

cDNA: complementary DNA
*Dscam1*: Down Syndrome cell adhesion molecule 1
GO: Gene Ontology
NCBI: National Center for Biotechnology Information
RT-PCR: reverse transcription PCR
