# Supplementary figures and images for: The modern expansion of Dscam1 isoform diversity in Drosophila is linked to fitness and immunity
Source: PLoS Biol. 2025 Sep 12;23(9):e3003383. doi: 10.1371/journal.pbio.3003383 (PMC12431208; doi:10.1371/journal.pbio.3003383)

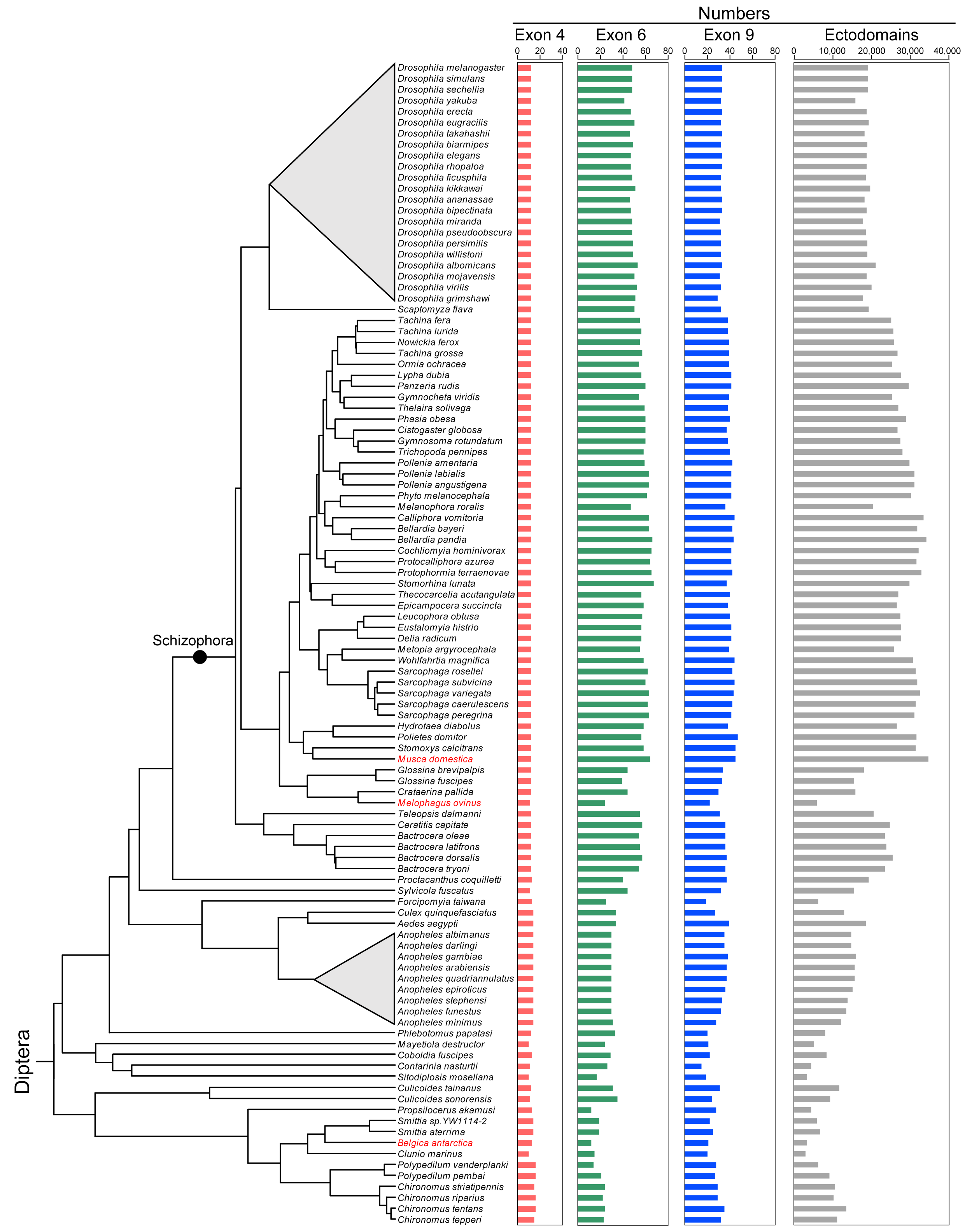

Supplement: S1 Fig — A phylogenetic tree of Dipteran species is shown on the left. The number of exons in variable exon 4, 6, and 9 cluster, and ectodomains diversity are shown in a different color on the right. Species with extreme Dscam1 isoform numbers are shown in red font. The data underlying this figure can be found in S1 Data. (TIF) [file pbio.3003383.s001.tif]

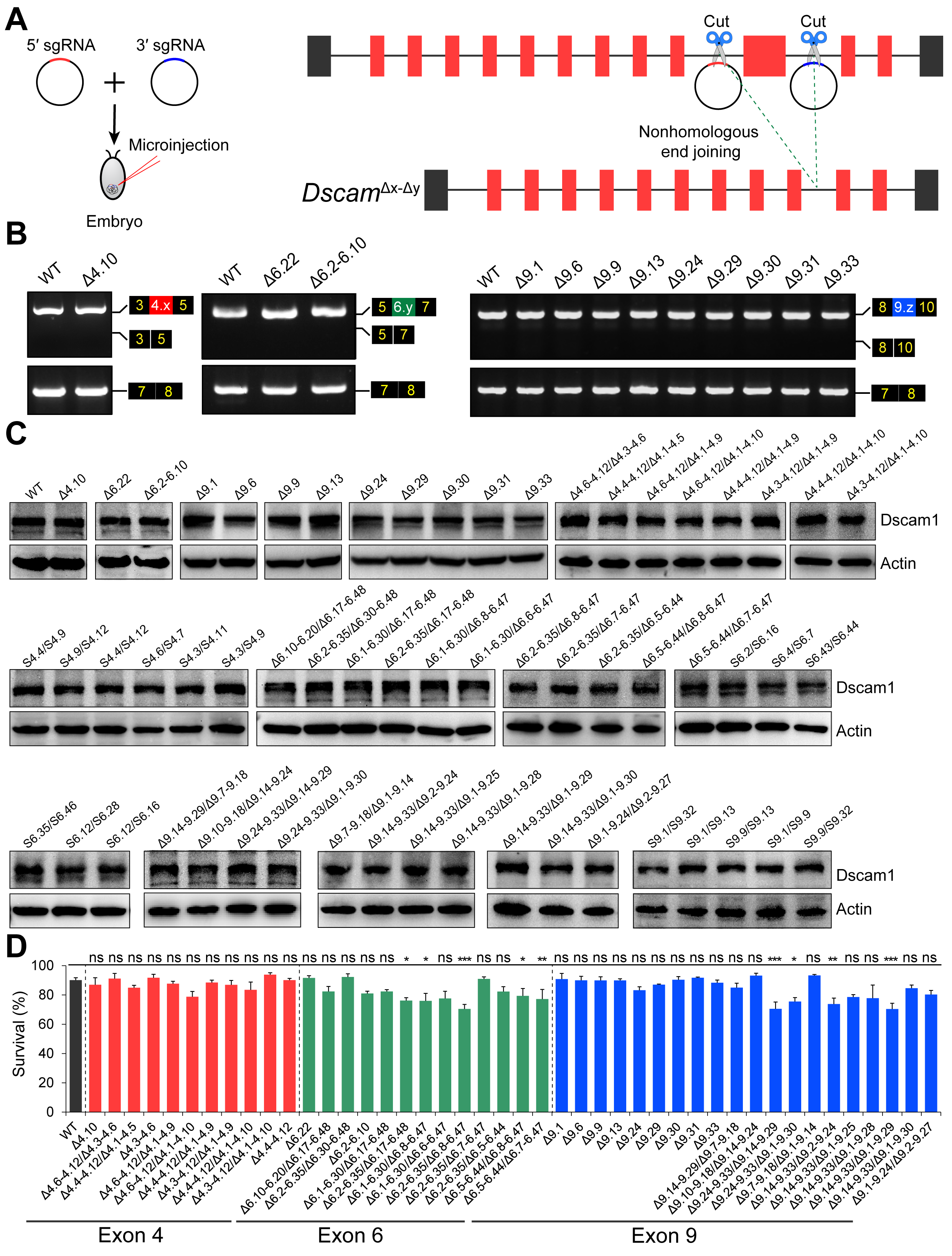

Supplement: S2 Fig — (A) Schematic diagram of the construction of Dscam1 variable exon deletion mutants. (B) RT-PCR diagram from the head tissues of wild-type and Dscam1 mutants. No obvious variable exon skipping or abnormal splicing was detected in Dscam1 mutants. (C) The protein levels in the head tissues of the Dscam1 mutants were similar to the wild-type controls. (D) Survival rates of the Dscam1 mutants and the wild-type control are shown. ns, not significant; *P < 0.05; **P < 0.01; ***P < 0.001 (one-way ANOVA with Dunnett’s test). The data underlying this figure can be found in S1 Data. (TIF) [file pbio.3003383.s002.tif]

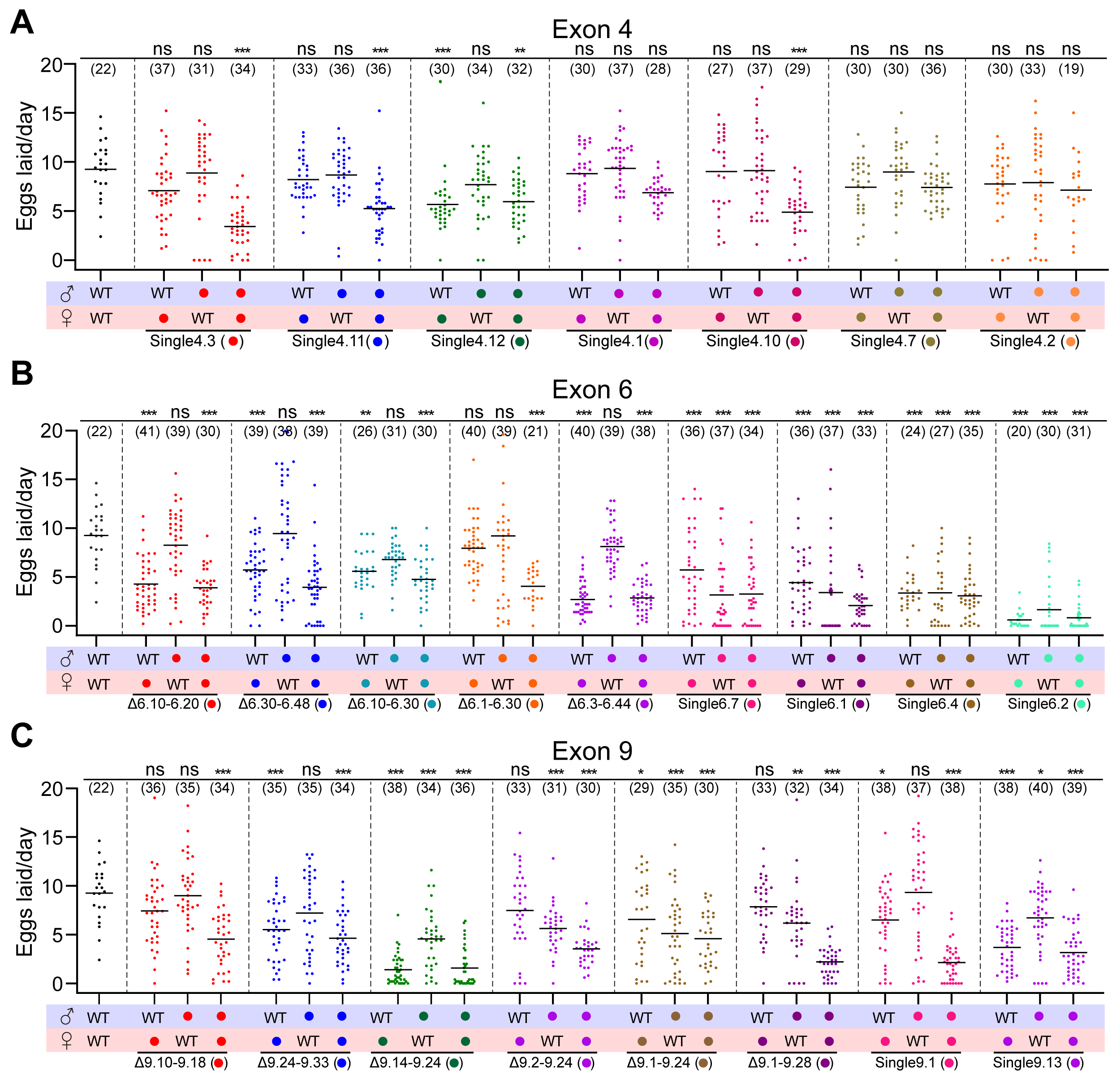

Supplement: S3 Fig — (A–C) Deletion of the Dscam1 isoform significantly reduced the fecundity of the mutant compared with the wild type. To explore the contribution of different sexes of Dscam1 mutant flies to reduced fecundity, female and male Dscam1 mutant flies were separately crossed with wild-type controls, and the fertility of the cross combinations was assessed. ns, not significant; *P < 0.05; **P < 0.01; ***P < 0.001 (one-way ANOVA with Dunnett’s test). The data underlying this figure can be found in S1 Data. (TIF) [file pbio.3003383.s003.tif]

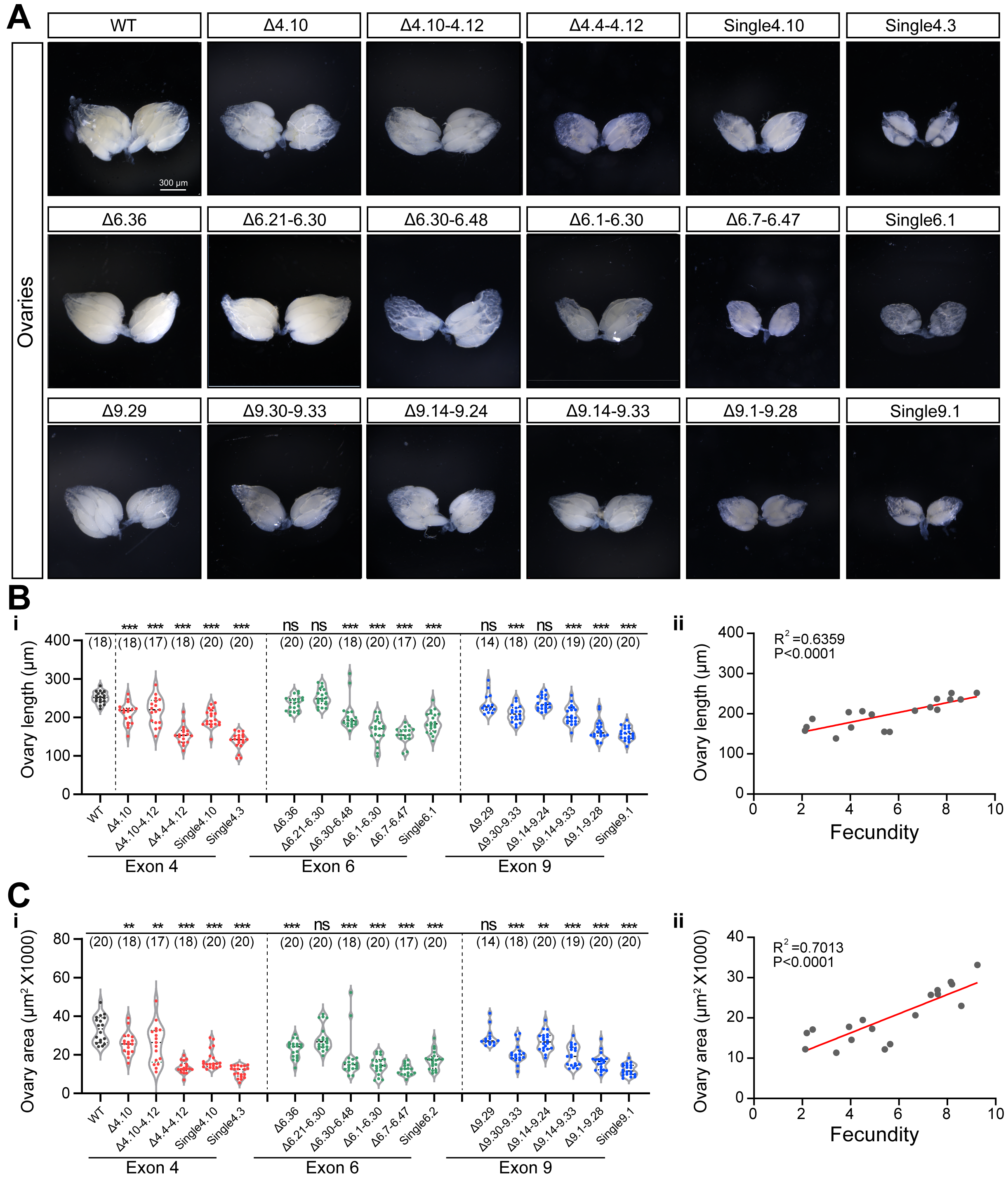

Supplement: S4 Fig — (A) Representative pictures of ovaries from Dscam1 mutants and the wild type. Dscam1 mutants showed small ovaries compared with the wild-type control. Scale bars, 300 µm. (B) Quantitative analysis of the ovary lengths of Dscam1 mutants compared with wild type. The fecundity was positively correlated with the ovary length. (C) Quantitative analysis of the ovary area of Dscam1 mutants compared with the wild type. The fecundity was positively correlated with the ovary area. Numbers in parentheses refer to the number of ovaries of each genotype studied. ns, not significant; *P < 0.05; **P < 0.01; ***P < 0.001 (one-way ANOVA with Dunnett’s test). The data underlying this figure can be found in S1 Data. (TIF) [file pbio.3003383.s004.tif]

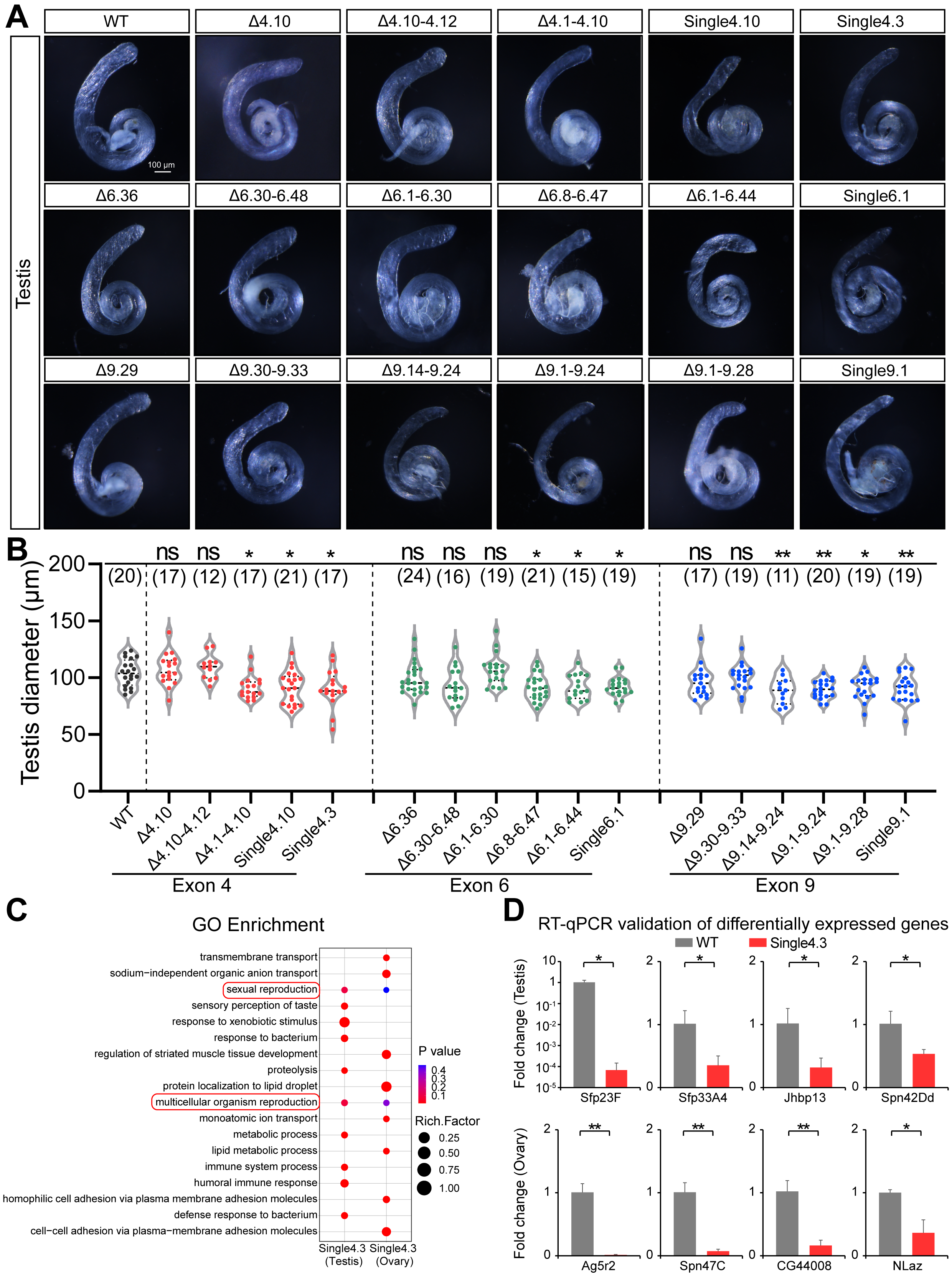

Supplement: S5 Fig — (A) Representative pictures of testis from Dscam1 mutants and the wild type. Scale bars, 100 µm. (B) Quantitative analysis of the diameter of the testis cross section of Dscam1 mutants compared with wild type. Numbers in parentheses refer to the number of testes of each genotype studied. ns, not significant; *P < 0.05; **P < 0.01 (one-way ANOVA with Dunnett’s test). (C) RNA-seq of ovarian and testicular tissues of DscamSingle4.3 mutants and wild type. GO enrichment analysis of differentially expressed genes revealed two reproduction-related terms: sexual reproduction and multicellular organism reproduction. (D) RT-qPCR validation of differentially expressed genes in these two terms. *P < 0.05; **P < 0.01 (Student t test, two-tailed). The data underlying this figure can be found in S1 Data. (TIF) [file pbio.3003383.s005.tif]

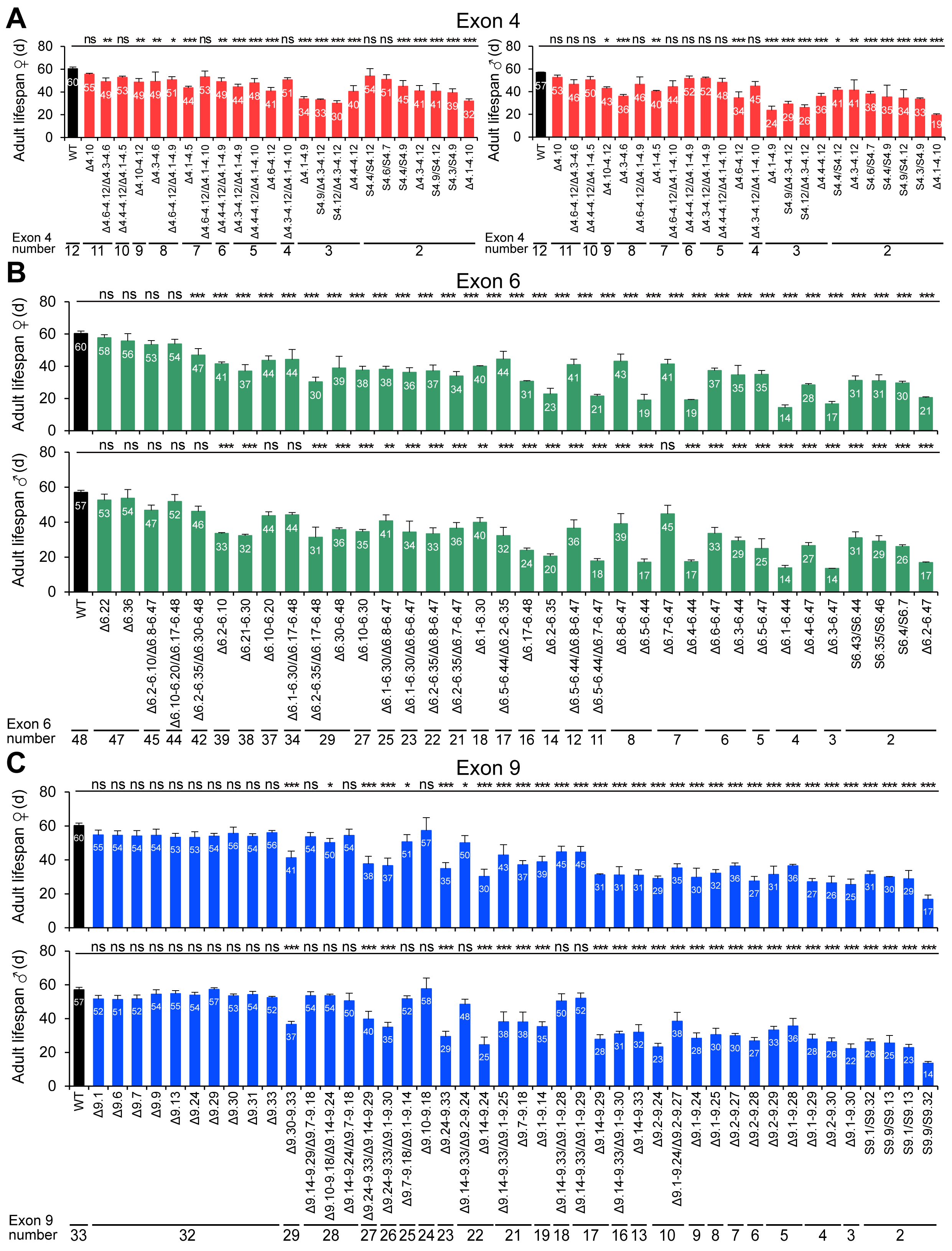

Supplement: S6 Fig — (A–C) Mean female and male adult lifespan of wild-type and Dscam1 mutants with deletion of variable exon 4 (A), exon 6 (B), and exon 9 (C) were calculated, respectively. The number of remaining variable exons of each mutant is shown at the bottom. *P < 0.05; **P < 0.01; ***P < 0.001; ns, not significant (one-way ANOVA with Dunnett’s test). The data underlying this figure can be found in S1 Data. (TIF) [file pbio.3003383.s006.tif]

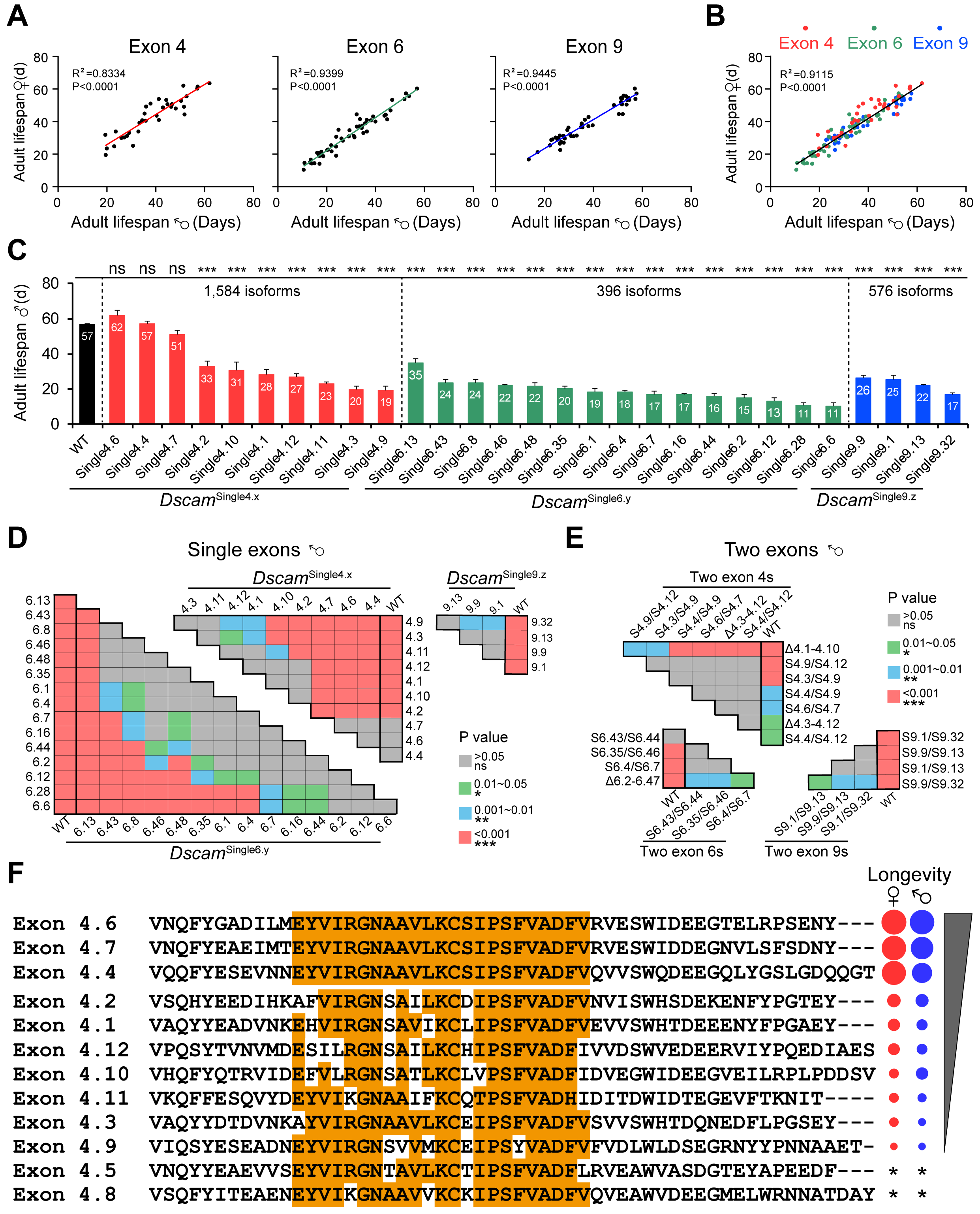

Supplement: S7 Fig — (A) Correlation analysis between the mean male lifespan and the mean female lifespan in exon 4, exon 6, and exon 9 genotypes, respectively. (B) Correlation analysis between the mean male lifespan and the mean female lifespan in Dscam1 mutants. Different variable clusters of mutants are shown as dots of different colors. (C) The comparison of the mean male lifespan between the wild-type and DscamSingle4.x, DscamSingle6.y, and DscamSingle9.z mutants. ns, not significant; ***P < 0.001 (one-way ANOVA with Dunnett’s test). (D, E) Pairwise comparisons (one-way ANOVA with Tukey’s test) were performed on the lifespan of a single variable exon mutants (D) or the two variable exon mutants (E), respectively. (F) Multiple sequence alignment of the 12 variable exon 4s showed that exons 4.4, 4.6, and 4.7 share a conserved amino acid region. The circles on the right represent the proportional changes in the lifespan of the corresponding DscamSingle4.x mutants. Due to abnormal splicing in DscamSingle4.5 and DscamSingle4.8 mutants [32], the symbol “*” indicates the absence of lifespan data for these two mutants. The data underlying this figure can be found in S1 Data. (TIF) [file pbio.3003383.s007.tif]

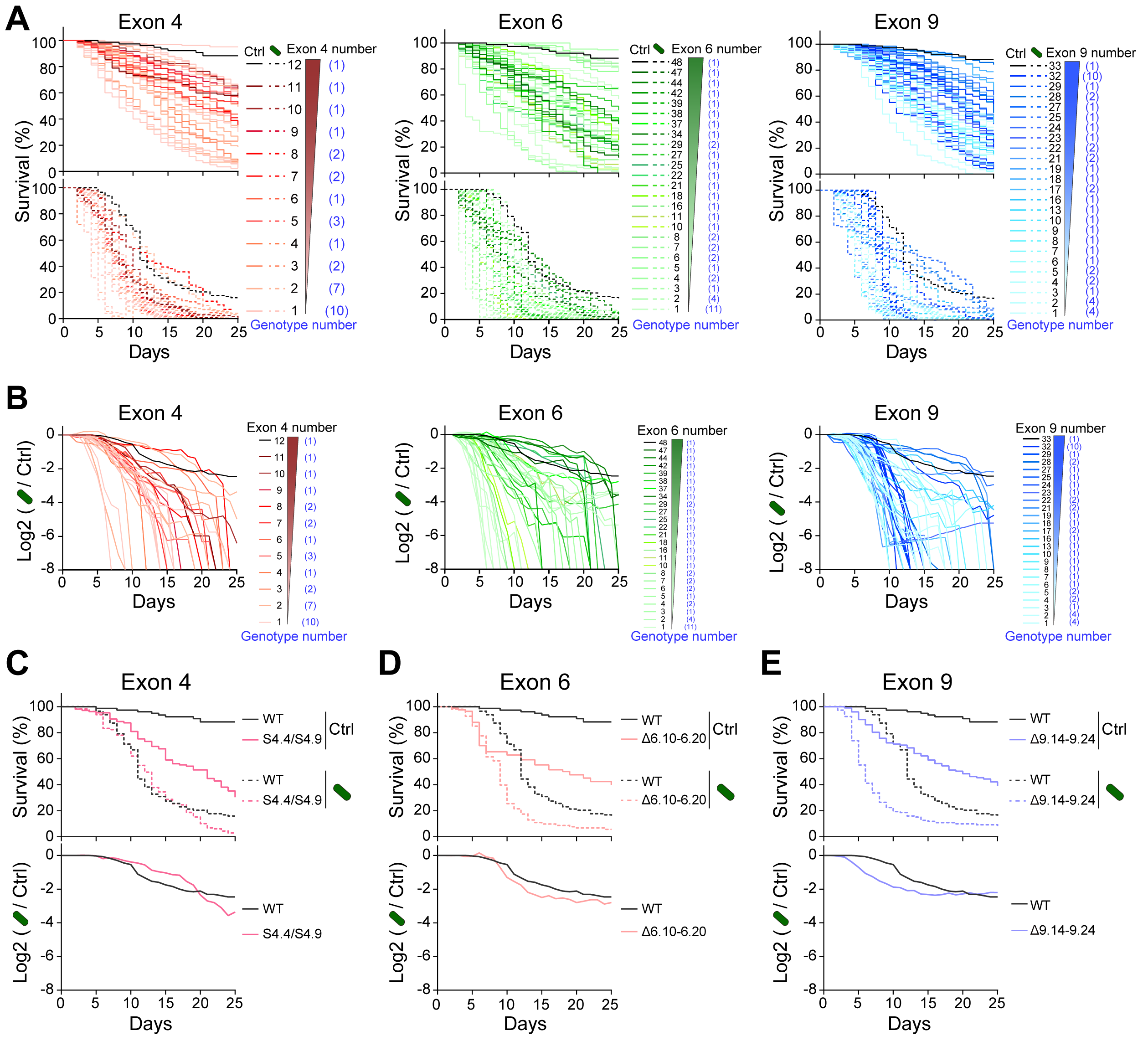

Supplement: S8 Fig — (A) The survival curves of exon 4, exon 6, and exon 9 mutant flies infected by Triton and M. robertsii are shown, respectively. The remaining variable exon numbers of the mutants are shown on the right. (B) The log2-transformed survival curves of M. robertsii infection group/blank group for Dscam1 exon 4, 6, and 9 mutants are shown, respectively. The remaining variable exon numbers of the mutants are shown on the right. (C–E) The survival curves and the log2-transformed survival curves upon M. robertsii infection for wild-type and exon 4 (DscamSingle4.4/single4.9) (C), exon 6 (Dscam∆6.10-6.20) (D), and exon 9 (Dscam∆9.14-9.24) (E) mutants are shown, the log2 fold change of the survival rate in these mutants was comparable with the WT control. The data underlying this figure can be found in S1 Data. (TIF) [file pbio.3003383.s008.tif]

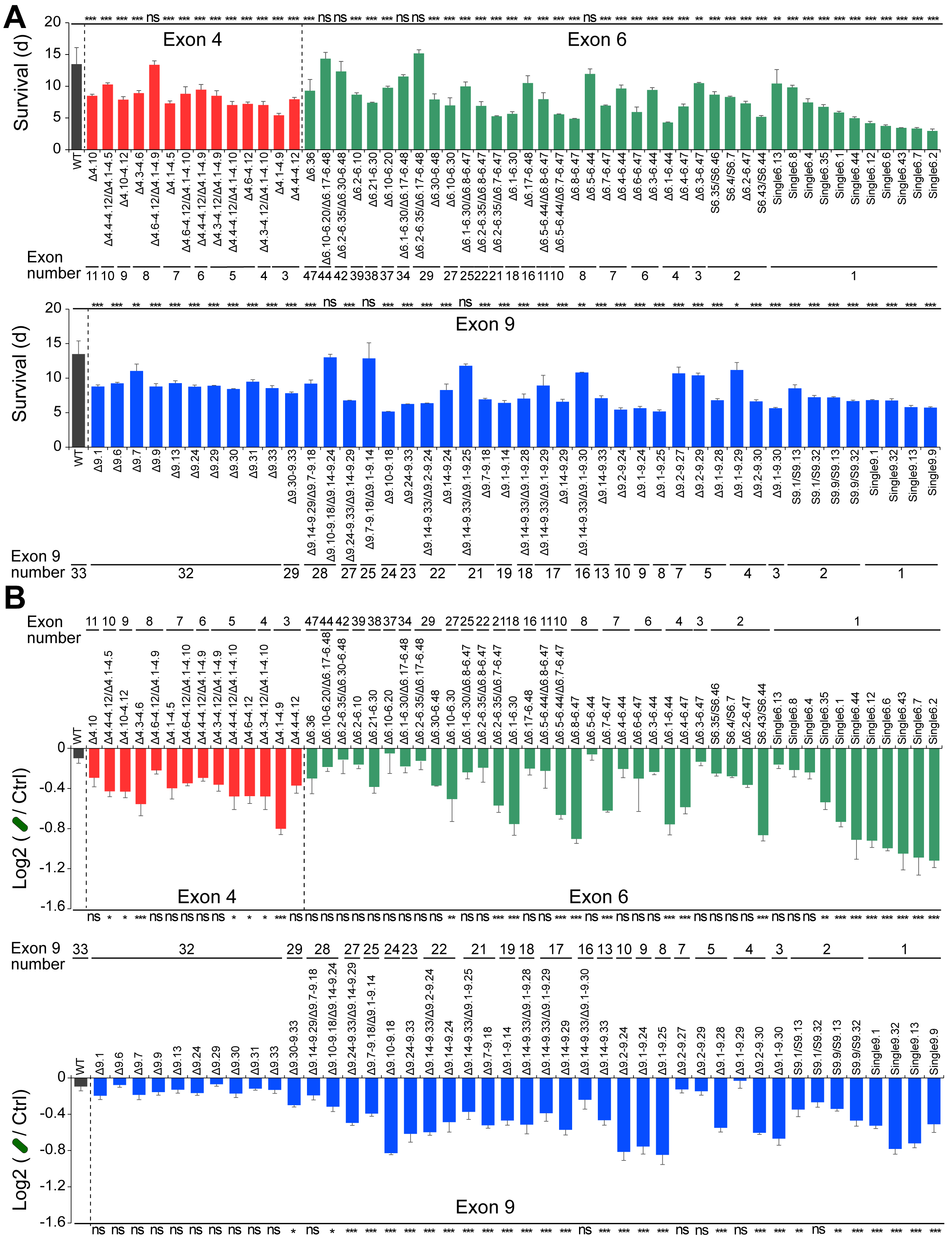

Supplement: S9 Fig — (A) The comparison of the mean survival days in the first 25 days of the infection group between the wild type and the Dscam1 mutants. (B) The mean survival days in the first 10 days of the infection group and the blank group were calculated, and the log2 value was obtained. The comparison of the mean log2 value between the wild type and the Dscam1 mutants. *P < 0.05; **P < 0.01; ***P < 0.001; ns, not significant (one-way ANOVA with Dunnett’s test). The data underlying this figure can be found in S1 Data. (TIF) [file pbio.3003383.s009.tif]

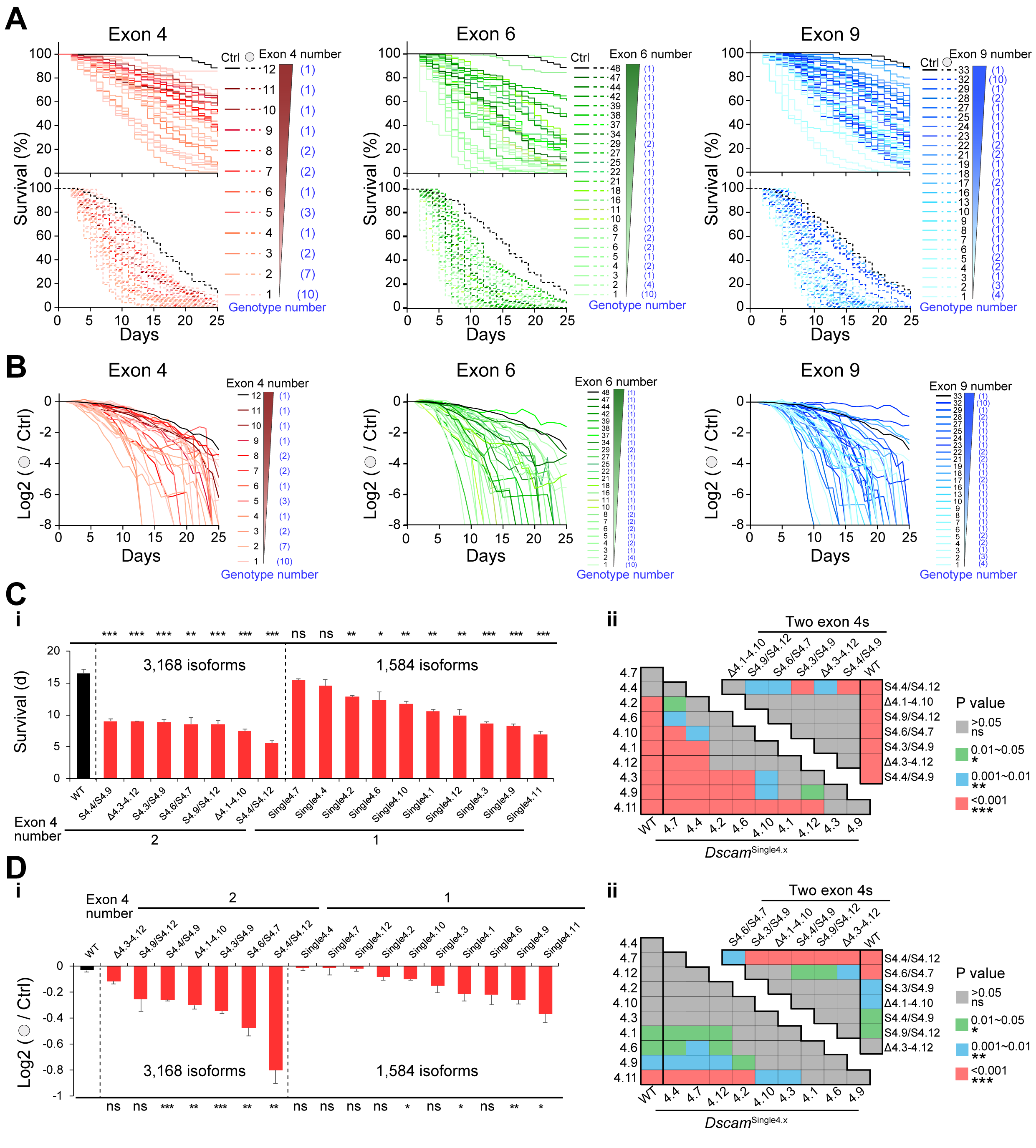

Supplement: S10 Fig — (A) The survival curves of exon 4, exon 6, and exon 9 mutant flies infected by Triton and B. bassiana are shown, respectively. The remaining variable exon numbers of the mutants are shown on the right. (B) The log2-transformed survival curves of B. bassiana infection group/blank group for Dscam1 exon 4, 6, and 9 mutants are shown, respectively. The remaining variable exon numbers of the mutants are shown on the right. (C) The comparison of the mean survival days in the first 25 days of the infection group between the wild type and the exon 4 mutants. Pairwise comparisons (one-way ANOVA with Tukey’s test) were performed on the survival days of the remaining single variable exon 4 or the remaining two variable exon 4 mutants, respectively. (D) The comparison of the mean log2 value in the first 10 days between the wild type and the exon 4 mutants. Pairwise comparisons (one-way ANOVA with Tukey’s test) were performed on the mean log2 value of a single variable exon 4 or the two variable exon 4 mutants, respectively. ns, not significant; *P < 0.05; **P < 0.01; ***P < 0.001. The data underlying this figure can be found in S1 Data. (TIF) [file pbio.3003383.s010.tif]

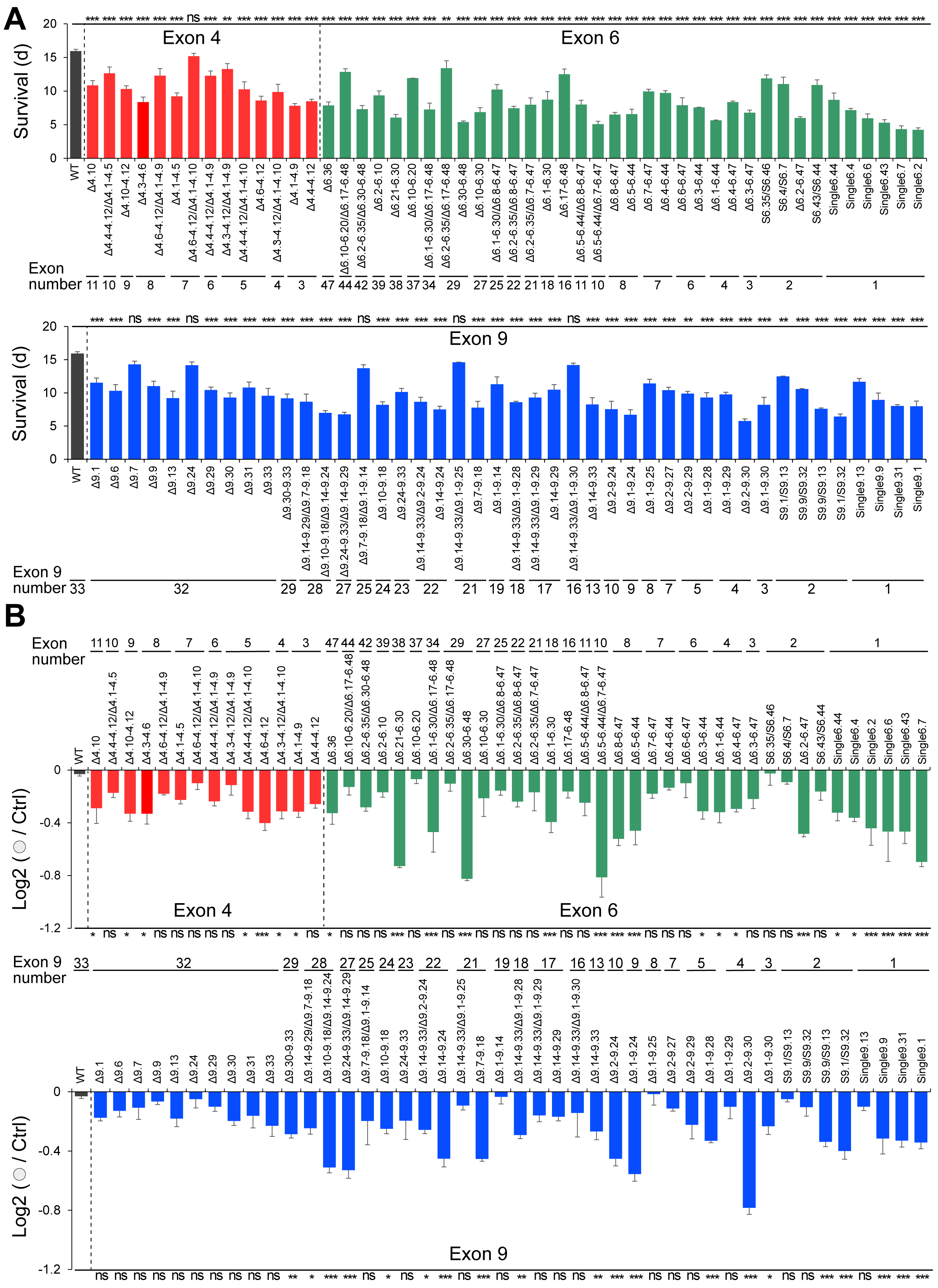

Supplement: S11 Fig — (A) The comparison of the mean survival days in the first 25 days of the infection group between the wild type and the Dscam1 mutants. (B) The mean survival days in the first 10 days of the infection group and the blank group were calculated, and the log2 value was obtained. The comparison of the mean log2 value between the wild type and the Dscam1 mutants. *P < 0.05; **P < 0.01; ***P < 0.001; ns, not significant (one-way ANOVA with Dunnett’s test). The data underlying this figure can be found in S1 Data. (TIF) [file pbio.3003383.s011.tif]

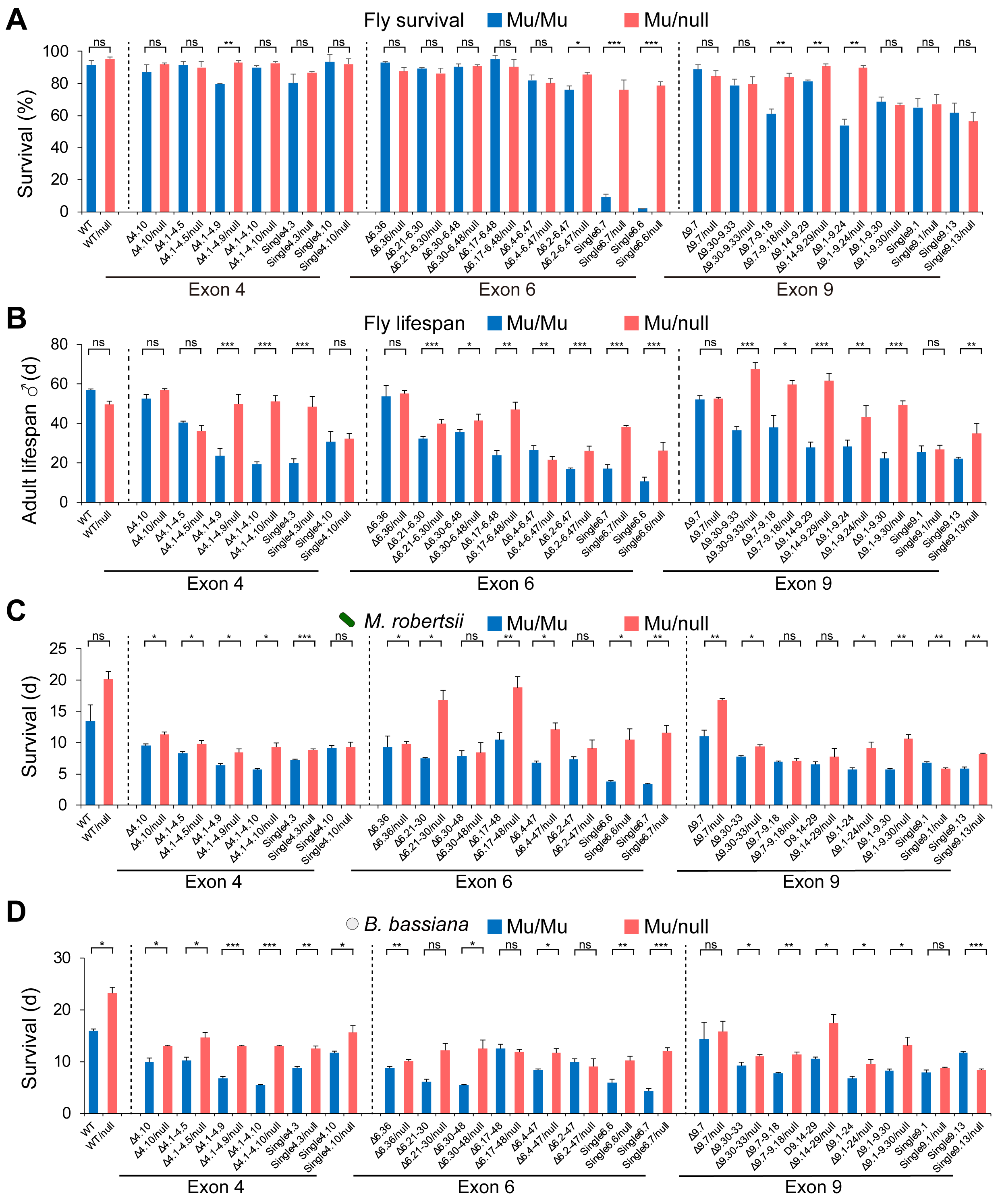

Supplement: S12 Fig — (A) The mean survival rates of adult flies of Dscam1 mutants with one copy or two copies of the mutant allele are shown. Reducing the Dscam1 expression level partially rescued the fly viability in homozygous mutants. (B) The mean lifespan of adult flies of Dscam1 mutants with one copy or two copies of the mutant allele is shown. (C) The log2-transformed survival rates and the mean survival days in the first 25 days of the Metarhizium robertsii infection of Dscam1 mutants with one copy or two copies of the mutant allele are shown. Reducing the Dscam1 expression level partially rescued the fly survival upon M. robertsii pathogen infection. (D) The log2-transformed survival rates and the mean survival days in the first 25 days of the Beauveria bassiana infection of Dscam1 mutants with one copy or two copies of the mutant allele are shown. *P < 0.05; **P < 0.01; ***P < 0.001; ns, not significant (Student t test, two-tailed). The data underlying this figure can be found in S1 Data. (TIF) [file pbio.3003383.s012.tif]

Raw images for Figure S2B and S2C

Fig S2B

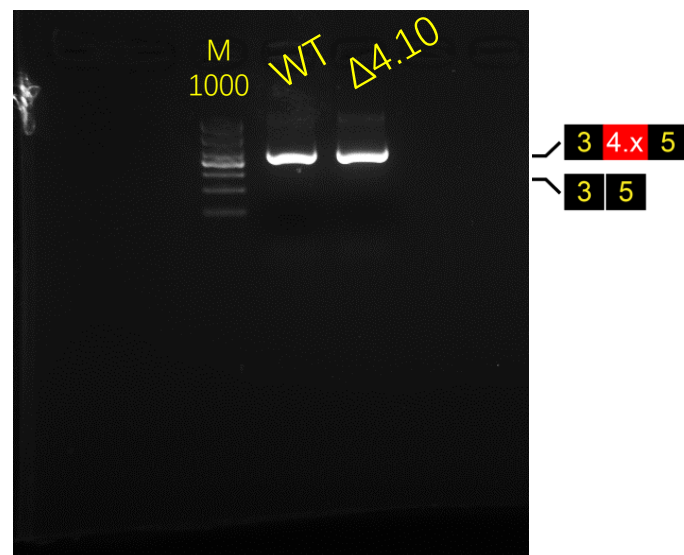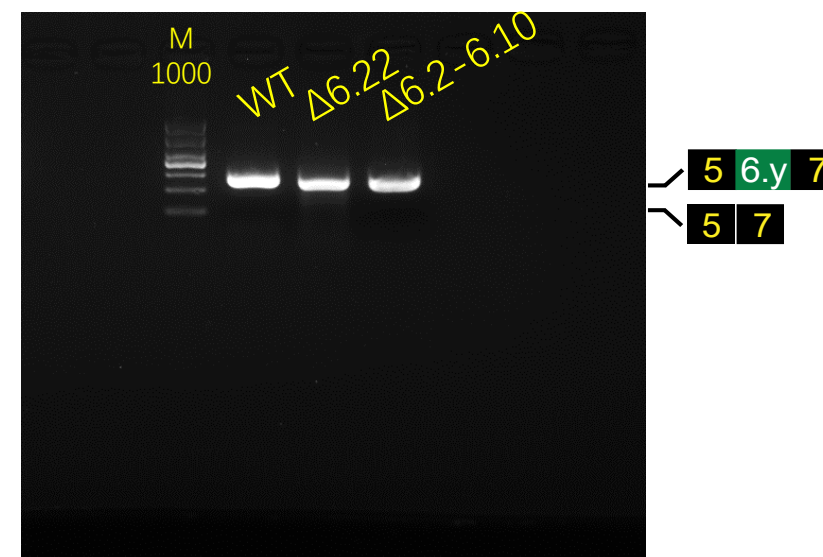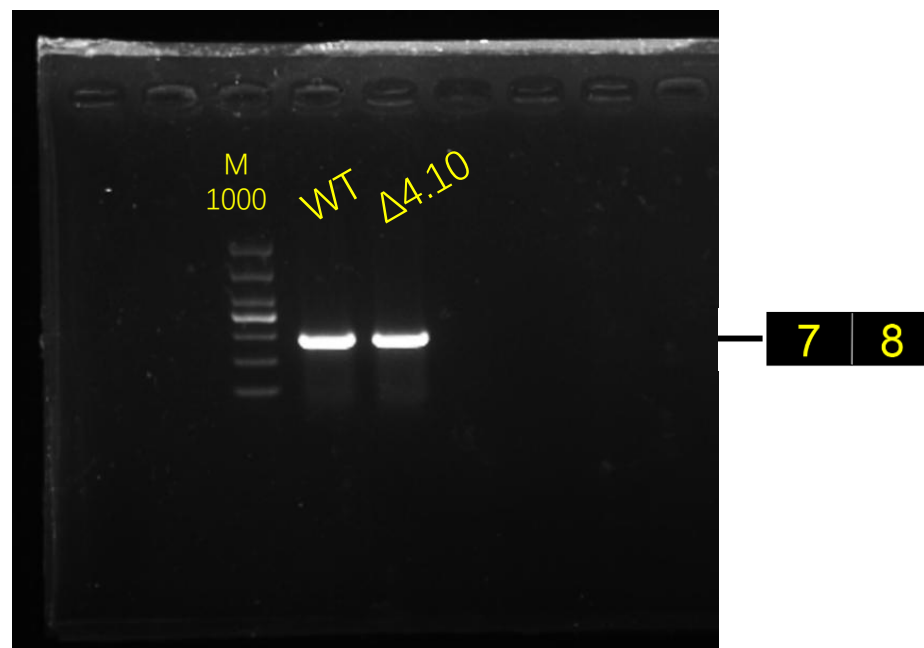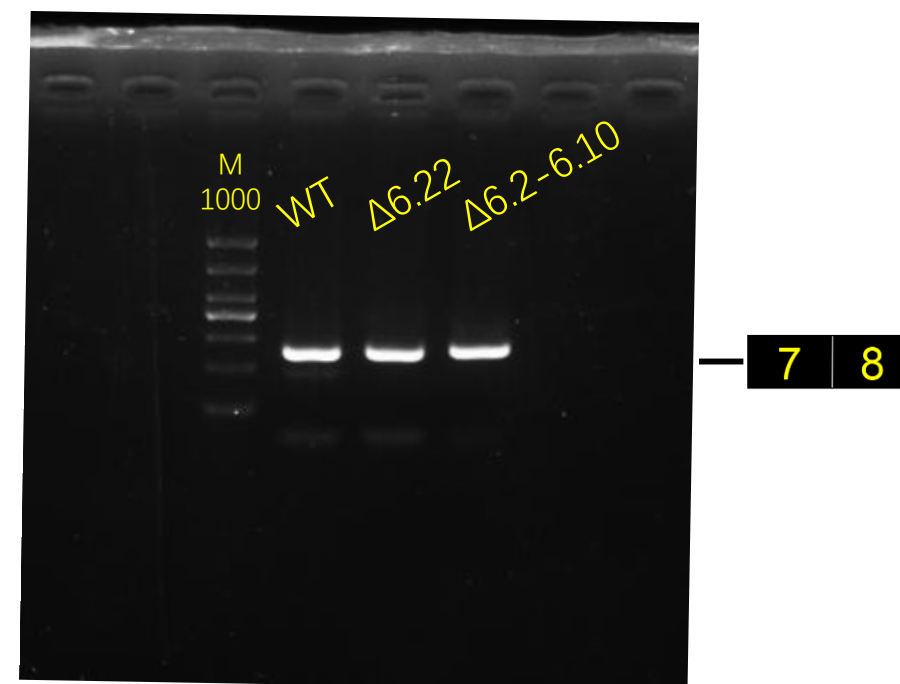

Fig S2B

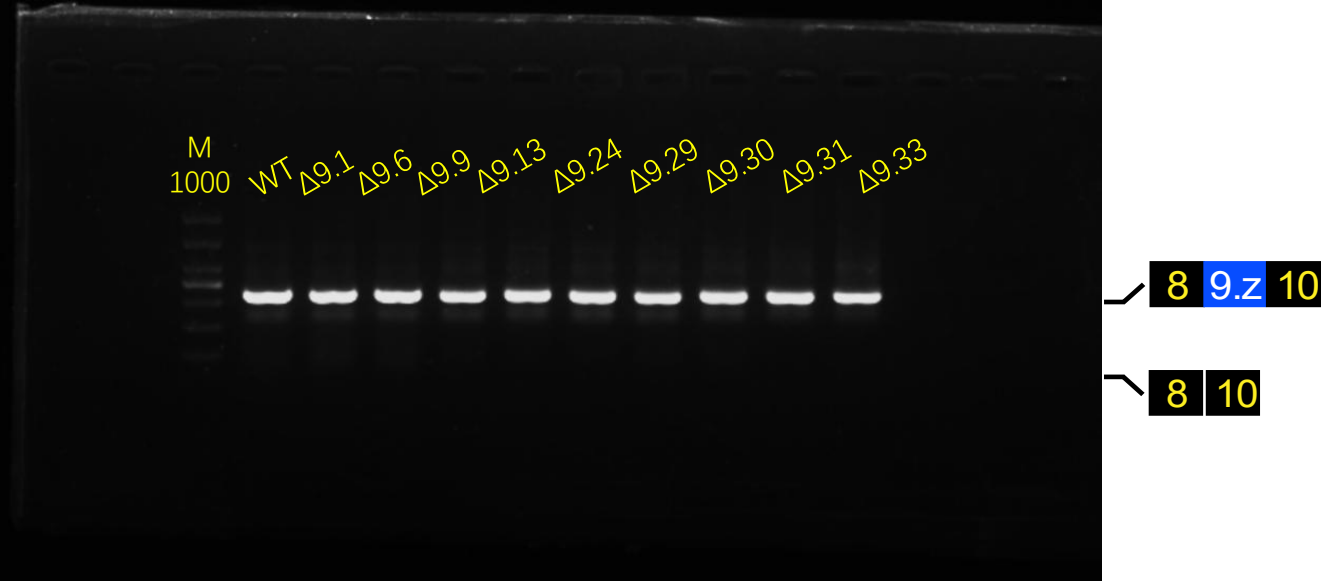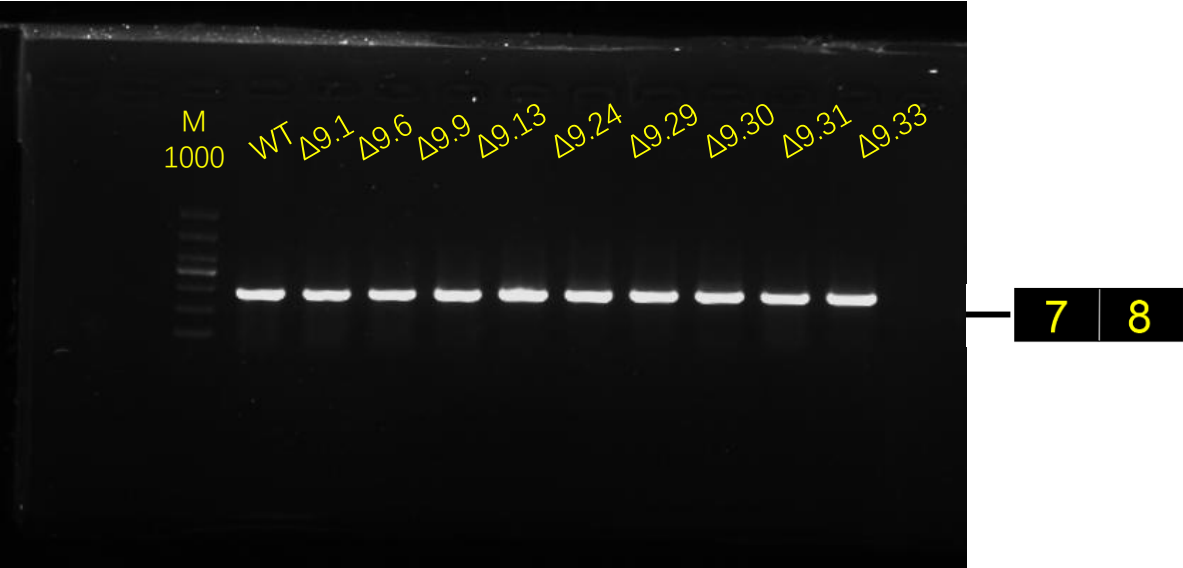

Fig S2C

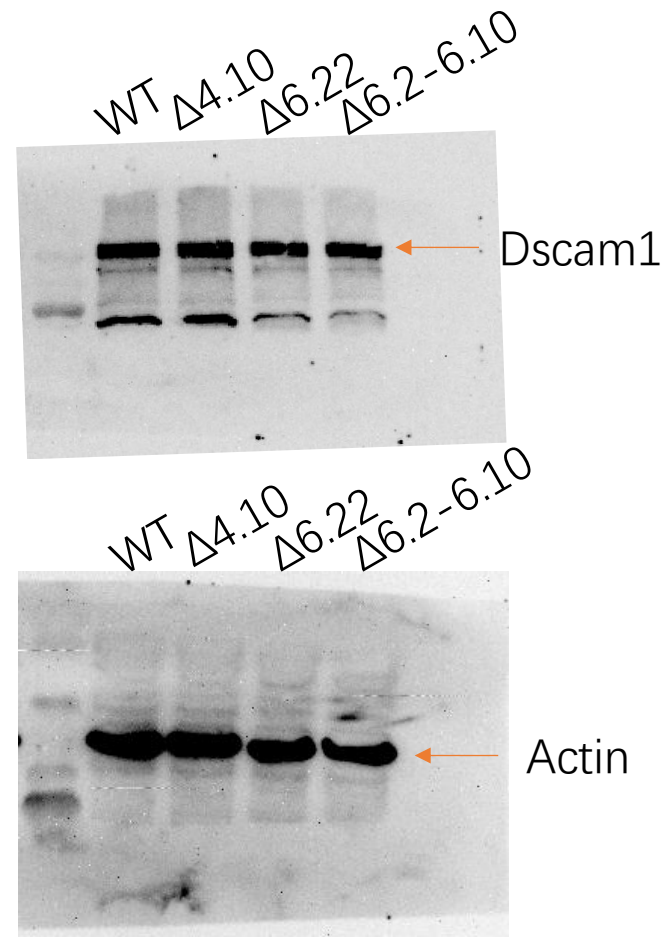

Fig S2C

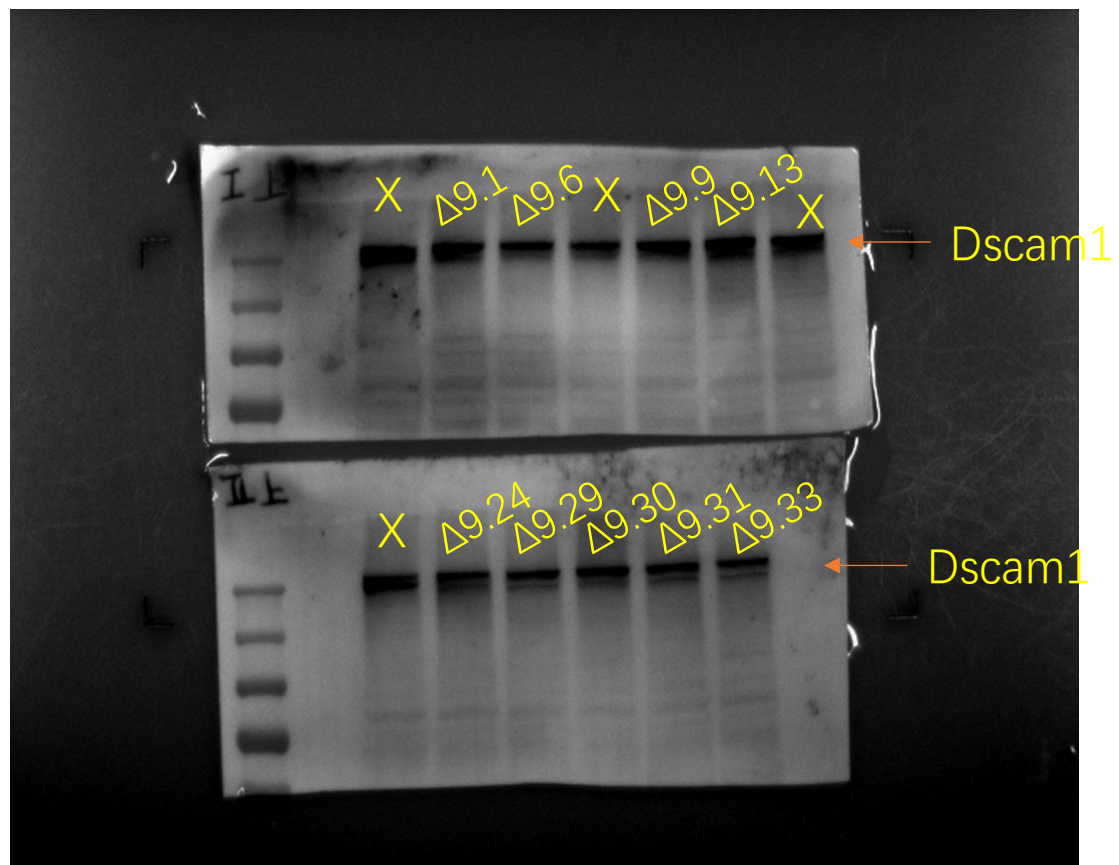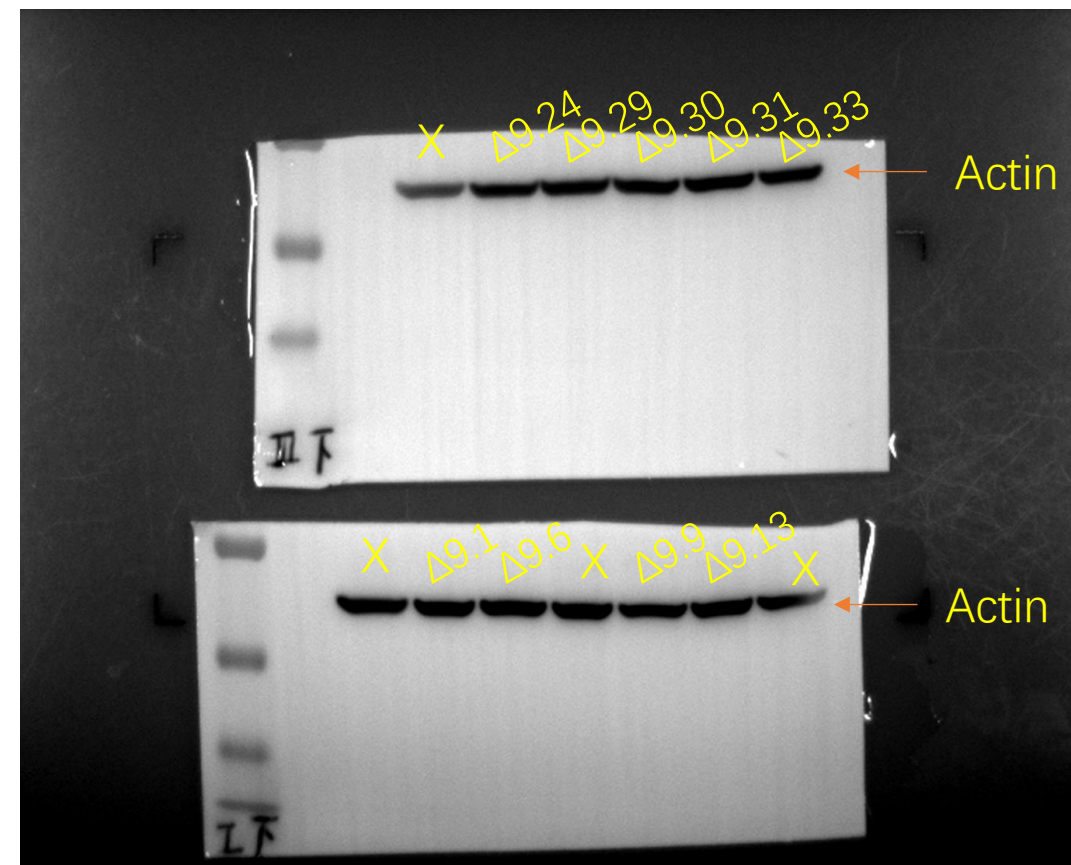

Fig S2C

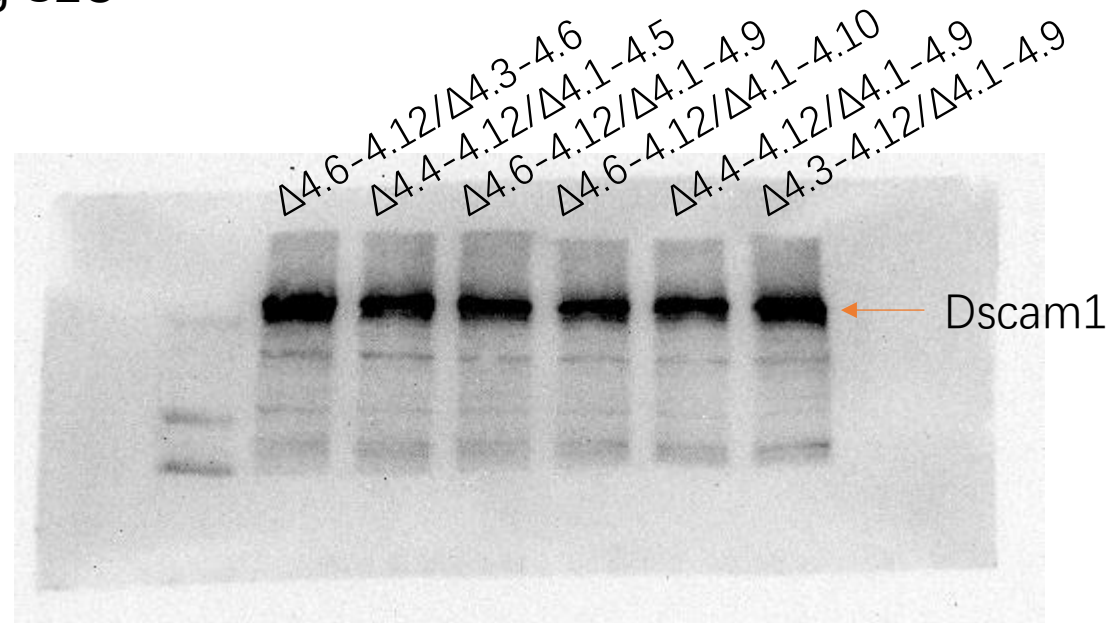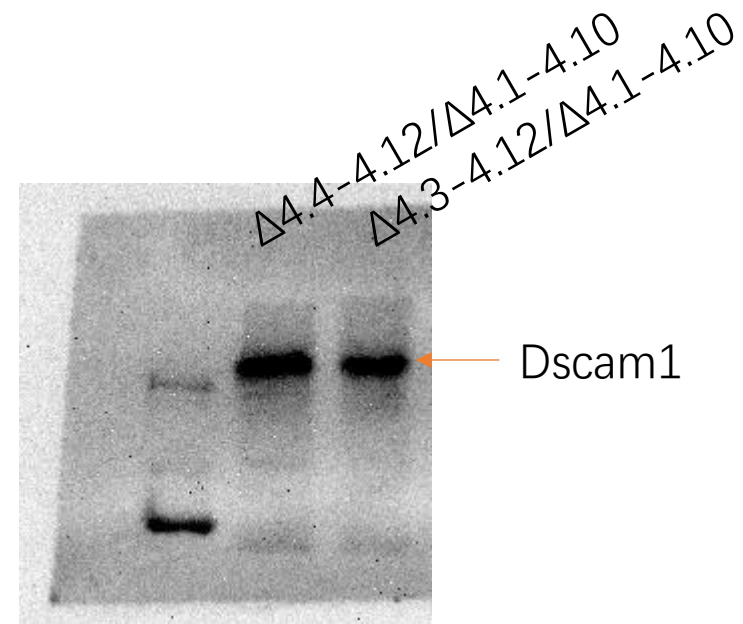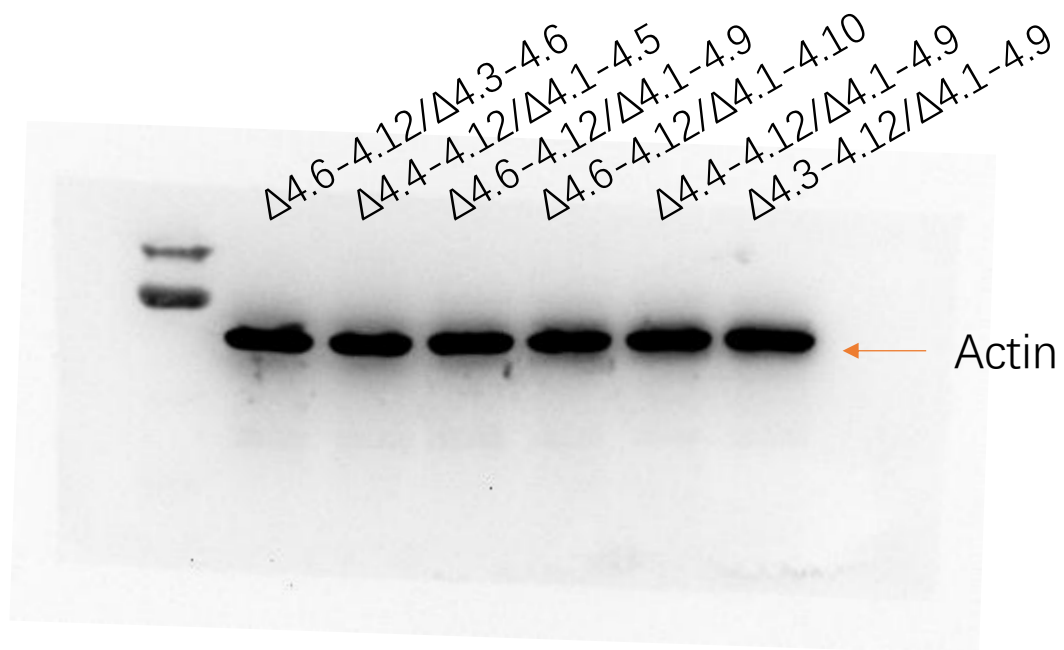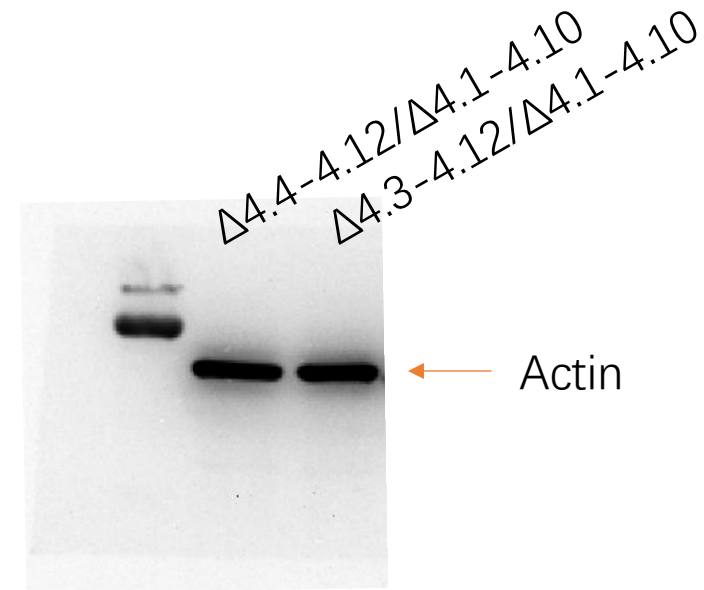

Fig S2C

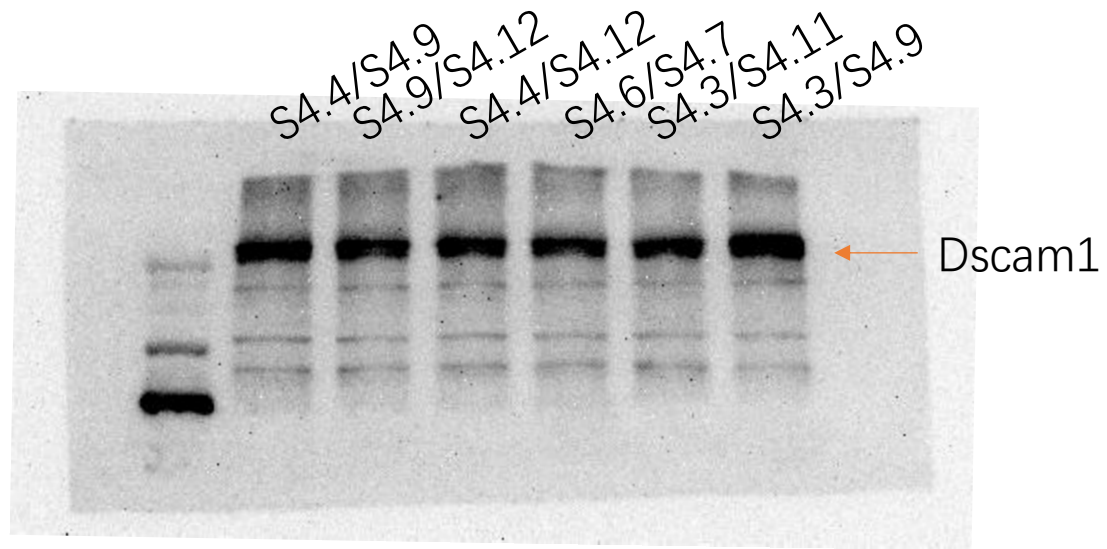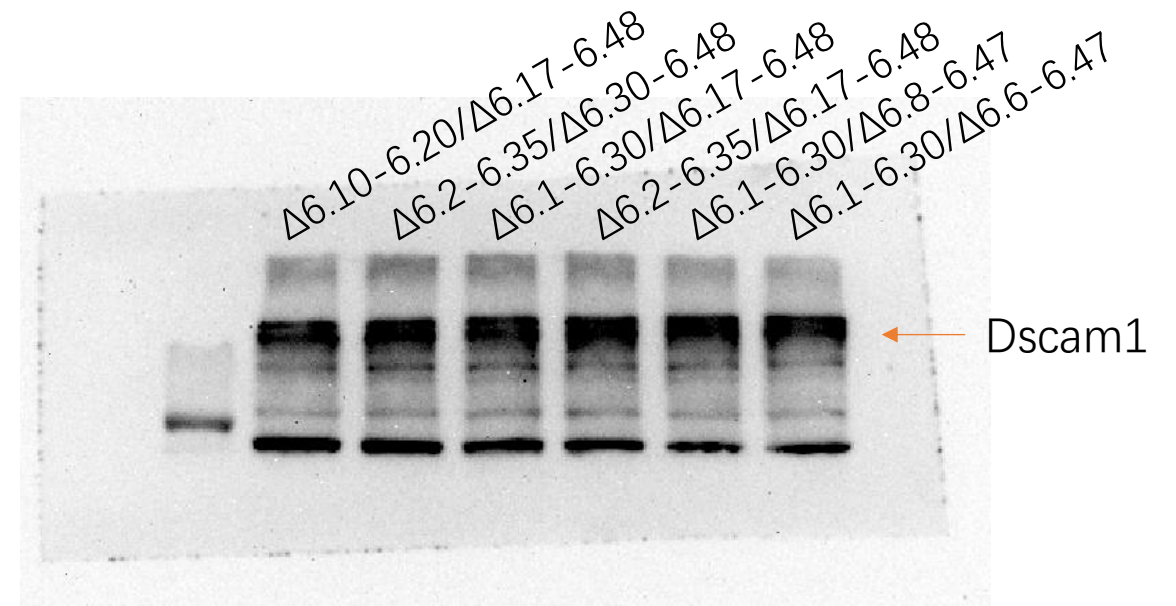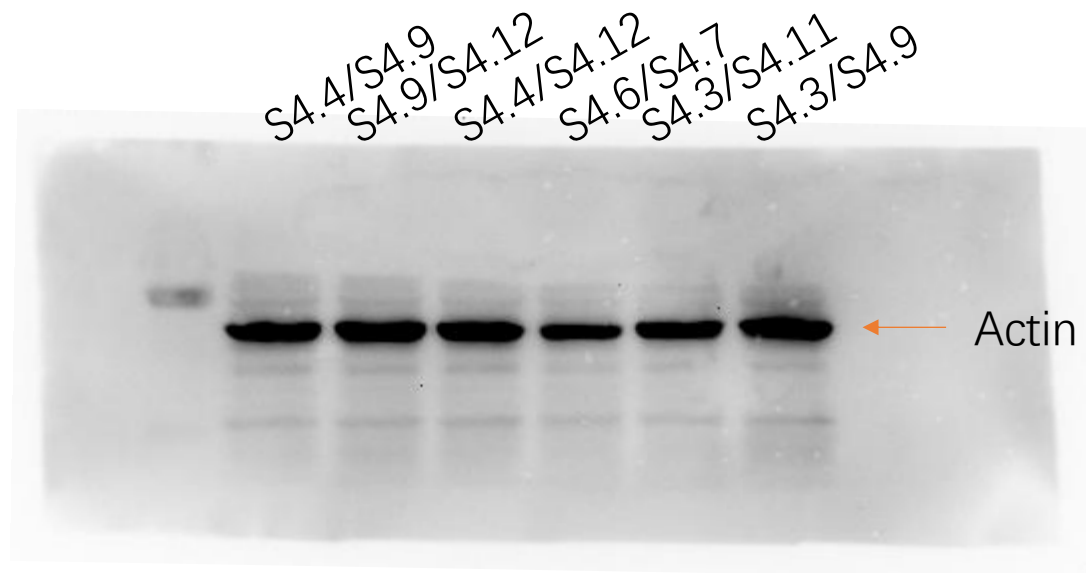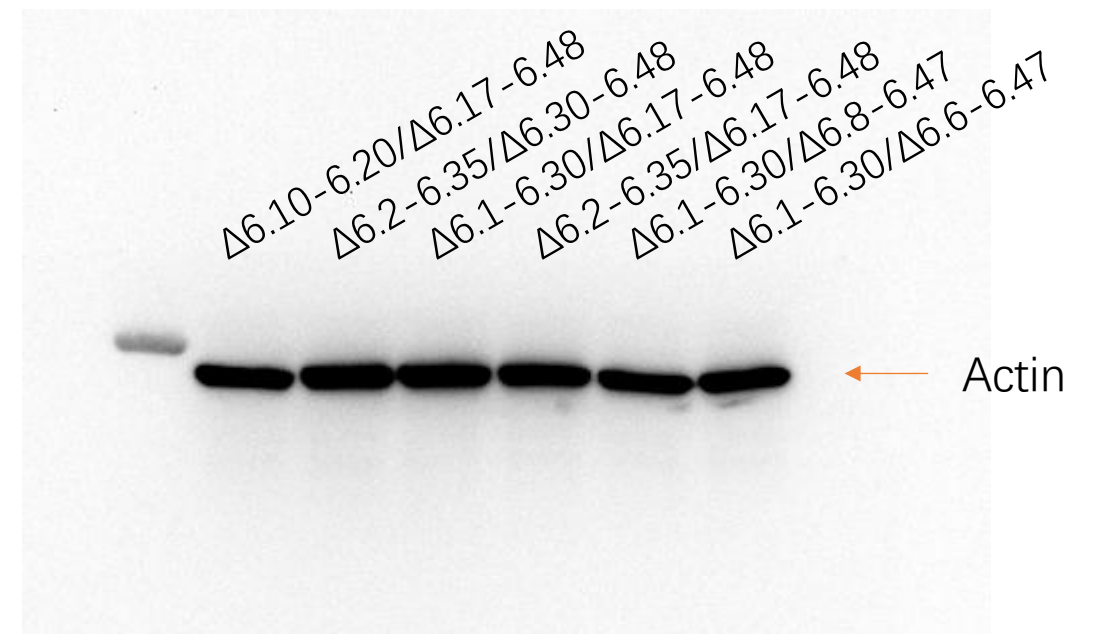

Fig S2C

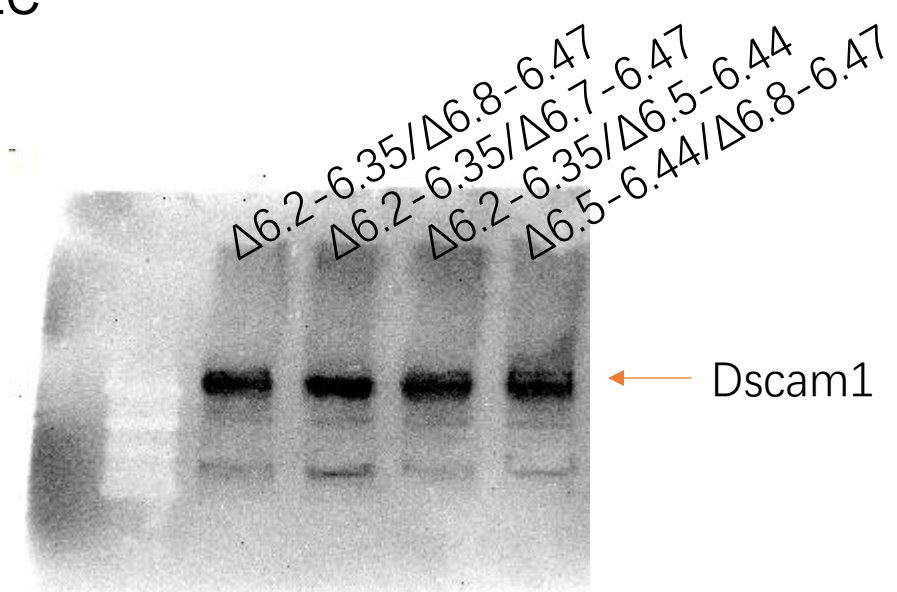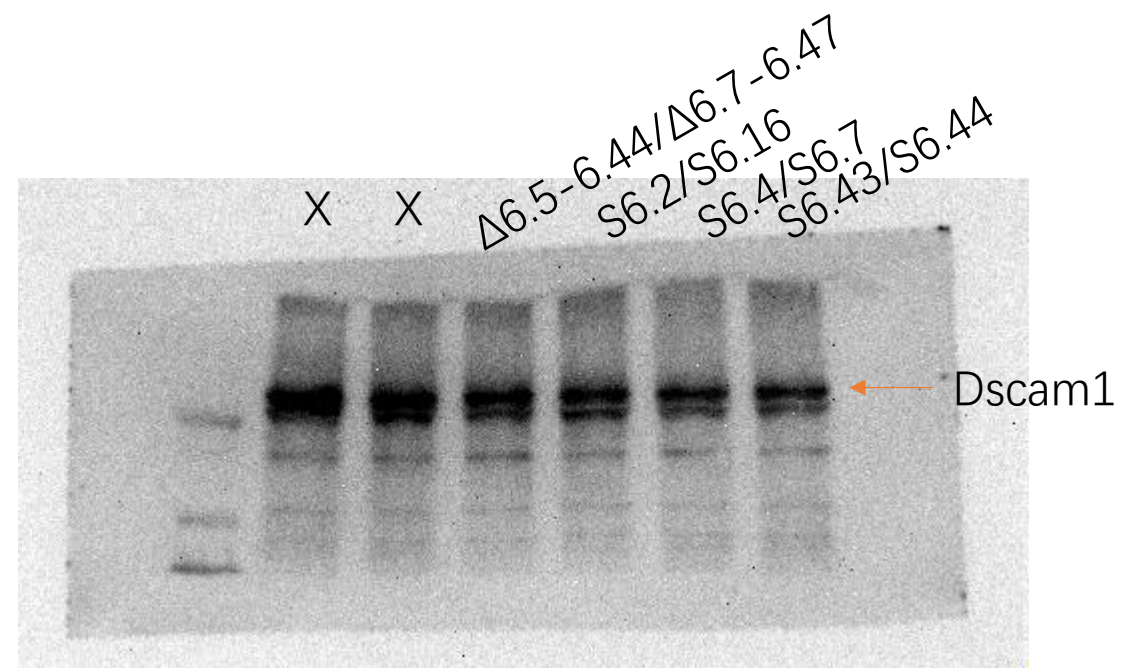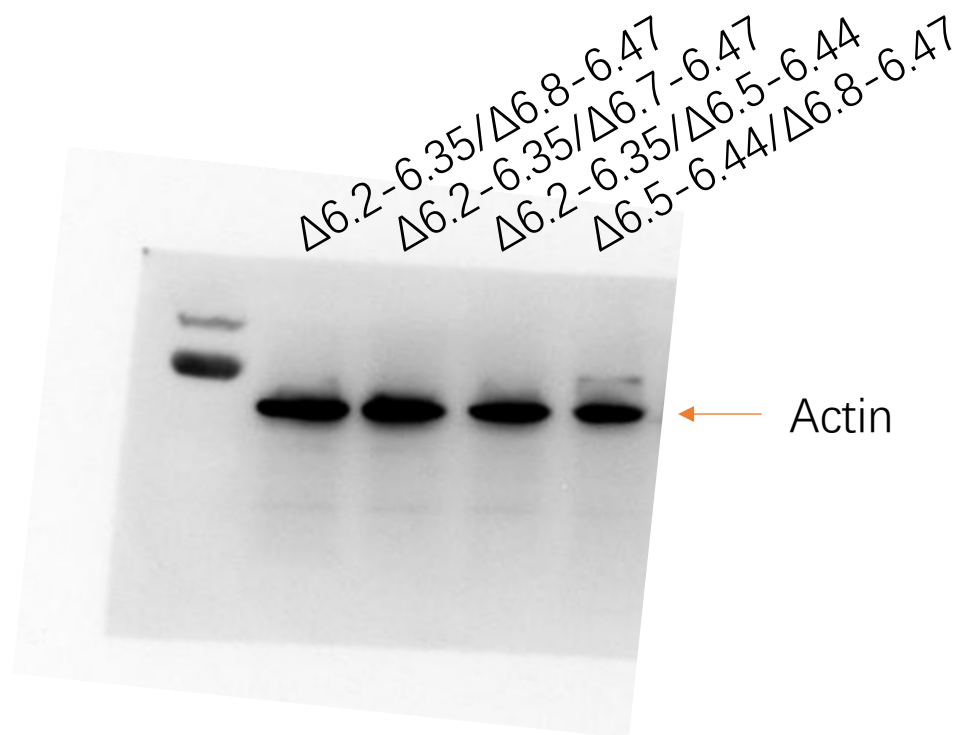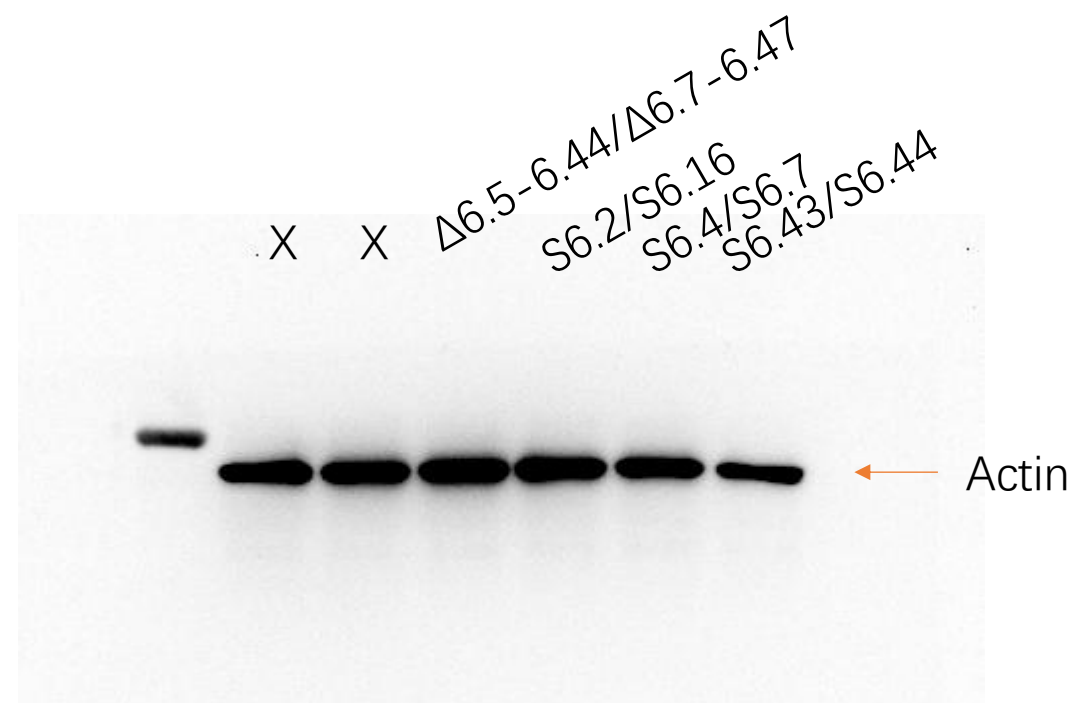

Fig S2C

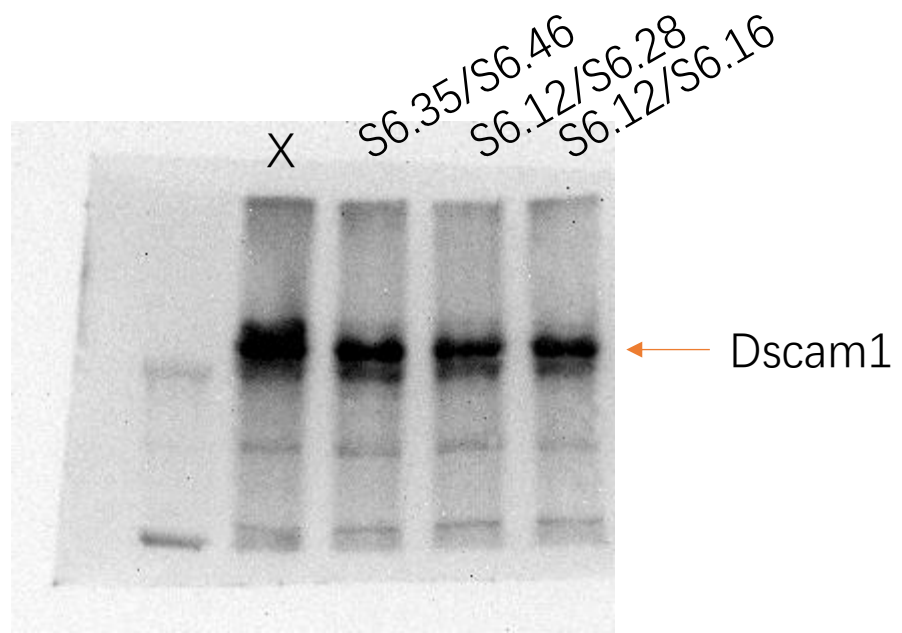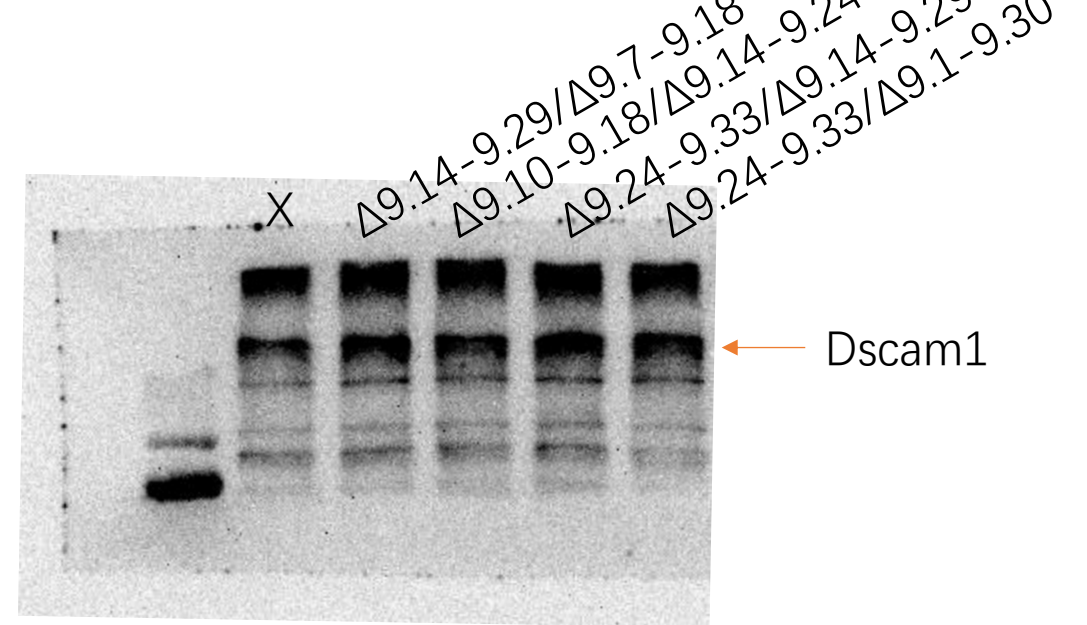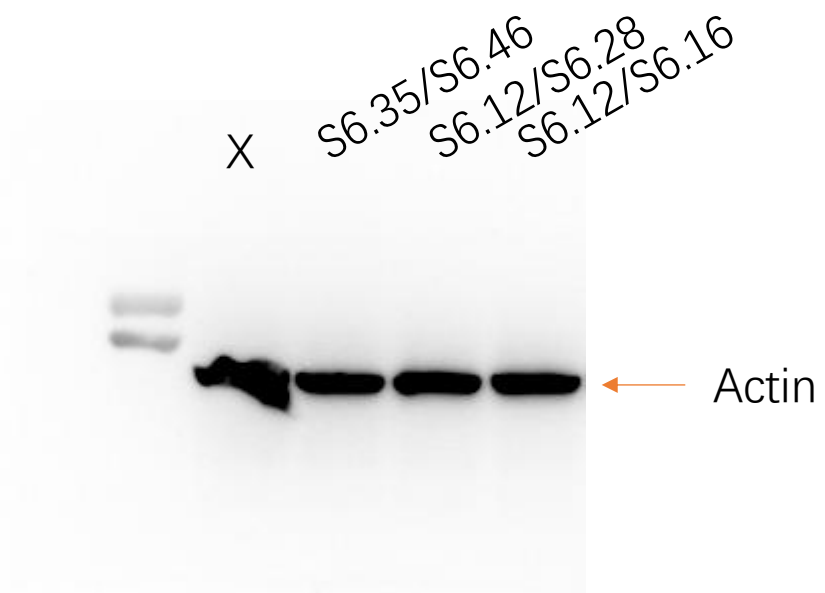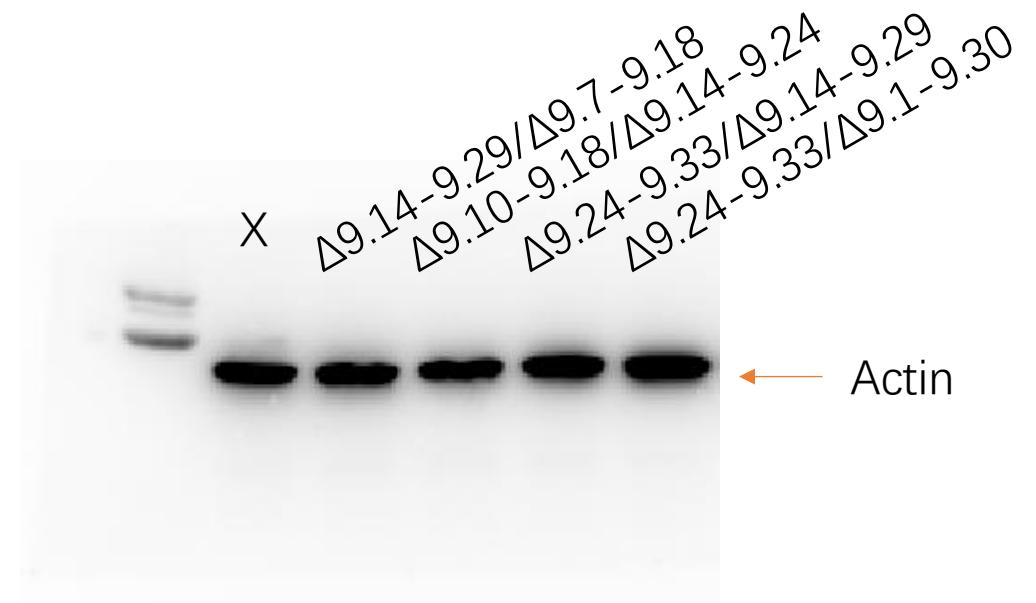

Fig S2C

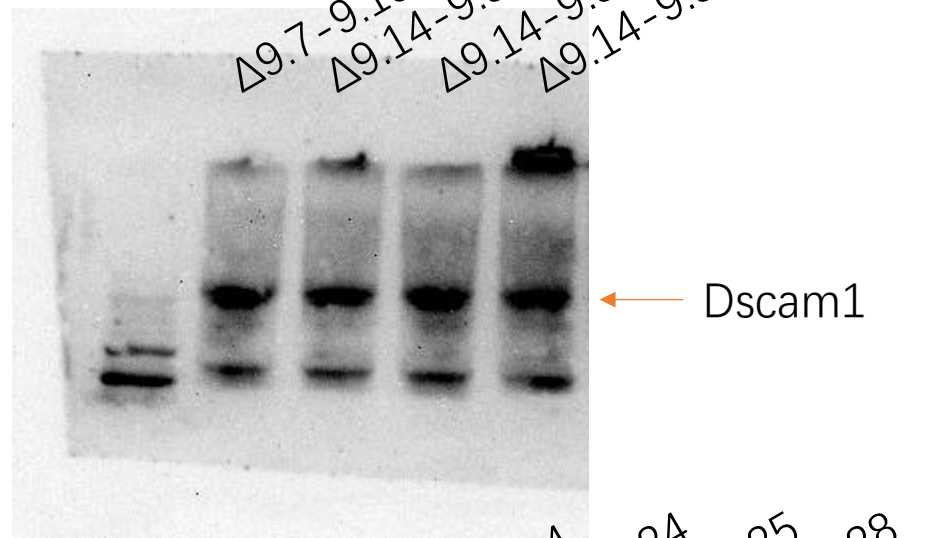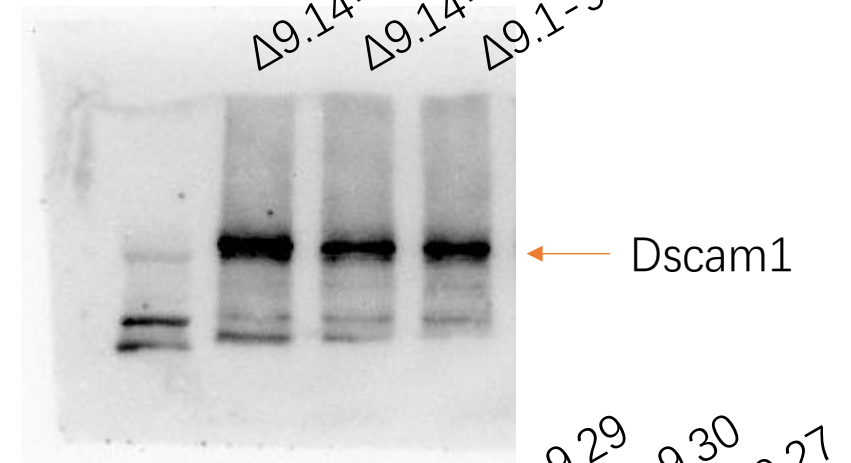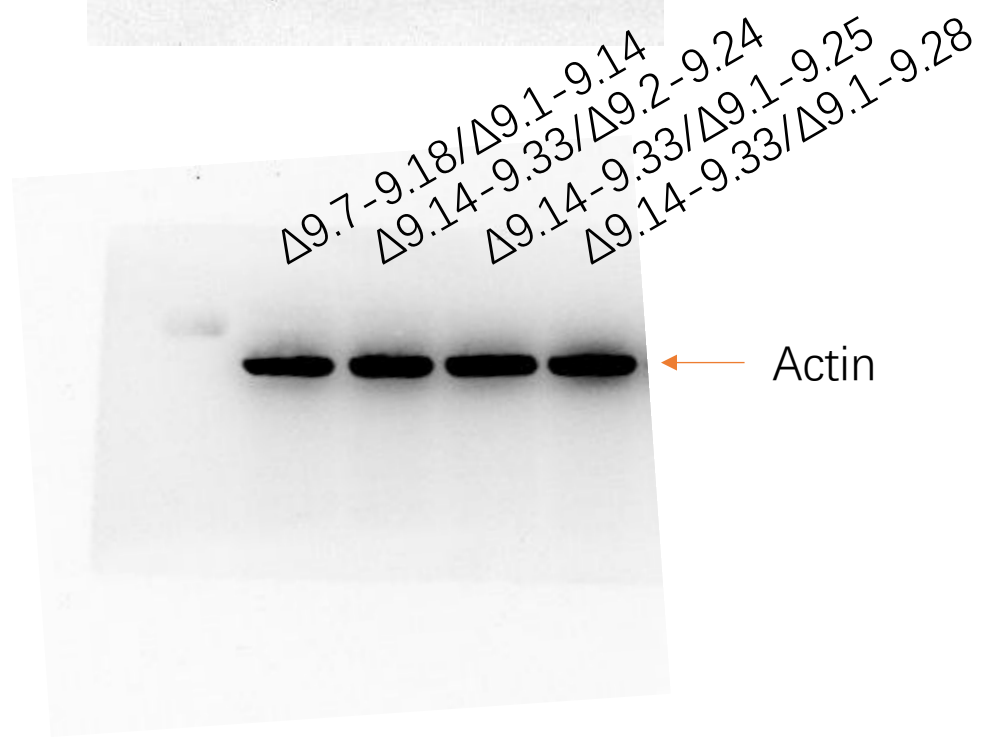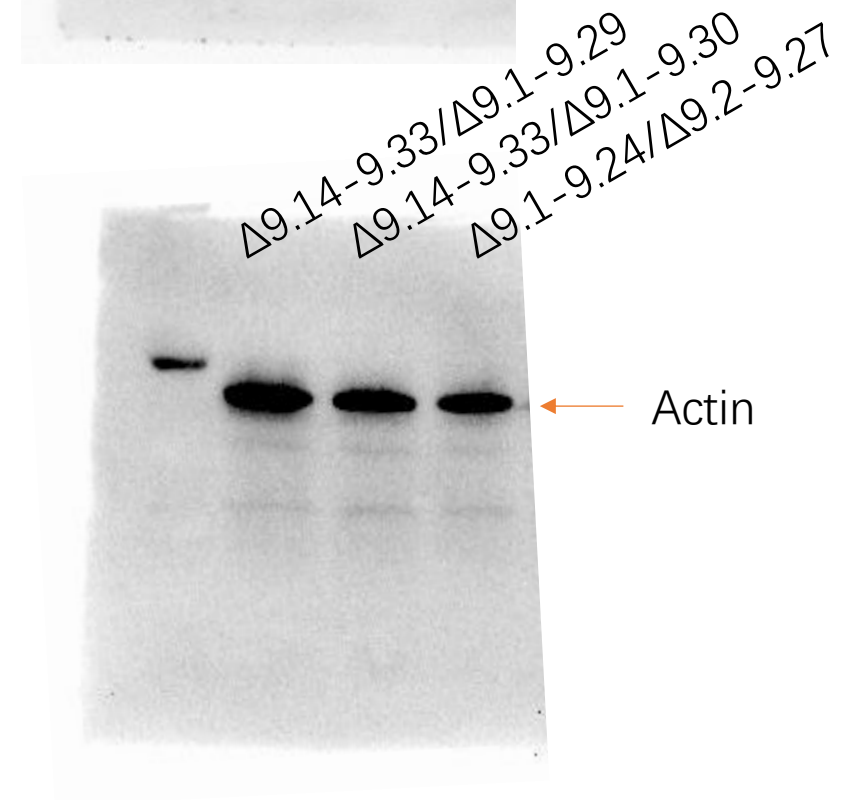

Fig S2C

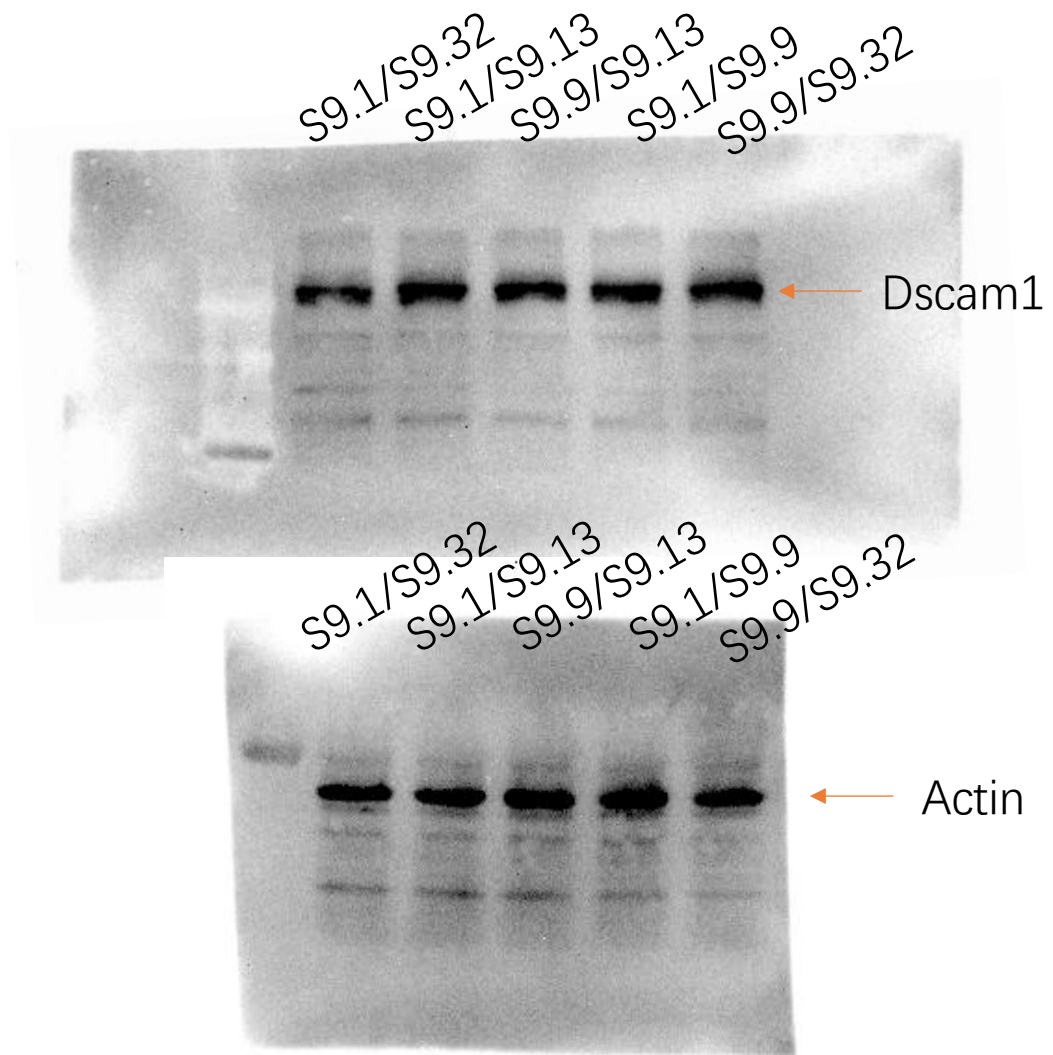

Supplement: S1 Raw Images — (PDF) [file pbio.3003383.s016.pdf]
