# Supplementary material for: The modern expansion of Dscam1 isoform diversity in Drosophila is linked to fitness and immunity
Source: PLoS Biol. 2025 Sep 12;23(9):e3003383. doi: 10.1371/journal.pbio.3003383 (PMC12431208; doi:10.1371/journal.pbio.3003383)
Supplement: S1 Table — (PDF) [file pbio.3003383.s013.pdf]

Table S1. Pancrustacea species used in this study. These species are listed according to class, order, family, genus and species. Dscam genomic obtained are indicated in the GenBank column. The number of variable exon 4, 6, 9, and potential ectodomain diversity in each species are shown on the right.

Class	Order	Family	Genus	Species	GenBank	E4 copy	E6 copy	E9 copy	Ectodomain
Insecta	Diptera	Drosophilidae	*Drosophila*	*D. melanogaster*	SIXD01000003.1	12	48	33	19008
Insecta	Diptera	Drosophilidae	*Drosophila*	*D. simulans*	JMCE01000003.1	12	48	33	19008
Insecta	Diptera	Drosophilidae	*Drosophila*	*D. sechellia*	NIFZ01000002.1	12	48	33	19008
Insecta	Diptera	Drosophilidae	*Drosophila*	*D. yakuba*	JAEIGT010000185.1	12	41	32	15744
Insecta	Diptera	Drosophilidae	*Drosophila*	*D. erecta*	AAPQ01007360.1	12	47	33	18612
Insecta	Diptera	Drosophilidae	*Drosophila*	*D. eugracilis*	AFPQ02006337.1	12	50	32	19200
Insecta	Diptera	Drosophilidae	*Drosophila*	*D. takahashii*	JAECXN010000110.1	12	46	33	18216
Insecta	Diptera	Drosophilidae	*Drosophila*	*D. biarmipes*	AFFD02007088.1	12	49	32	18816
Insecta	Diptera	Drosophilidae	*Drosophila*	*D. elegans*	AFFF02008285.1	12	47	33	18612
Insecta	Diptera	Drosophilidae	*Drosophila*	*D. rhopaloa*	JAECXI010000115.1	12	47	33	18612
Insecta	Diptera	Drosophilidae	*Drosophila*	*D. ficusphila*	AFFG02007939.1	12	48	32	18432
Insecta	Diptera	Drosophilidae	*Drosophila*	*D. kikkawai*	AFFH02007496.1	12	51	32	19584
Insecta	Diptera	Drosophilidae	*Drosophila*	*D. ananassae*	AAPP01018724.1	12	46	33	18216
Insecta	Diptera	Drosophilidae	*Drosophila*	*D. bipectinata*	AFFE02007328.1	12	47	33	18612
Insecta	Diptera	Drosophilidae	*Drosophila*	*D. miranda*	AJMI02001995.1	12	48	31	17856
Insecta	Diptera	Drosophilidae	*Drosophila*	*D. pseudoobscura*	AADE02000012.1	12	48	32	18432
Insecta	Diptera	Drosophilidae	*Drosophila*	*D. persimilis*	QMET02000002.1	12	49	32	18816
Insecta	Diptera	Drosophilidae	*Drosophila*	*D. willistoni*	AAQB01006282.1	12	49	32	18816
Insecta	Diptera	Drosophilidae	*Drosophila*	*D. albomicans*	BJEI01000001.1	12	53	33	20988
Insecta	Diptera	Drosophilidae	*Drosophila*	*D. mojavensis*	AAPU01010180.1	12	50	31	18600
Insecta	Diptera	Drosophilidae	*Drosophila*	*D. virilis*	AANI01014858.1	12	52	32	19968
Insecta	Diptera	Drosophilidae	*Drosophila*	*D. grimshawi*	AAPT01021484.1	12	51	29	17748
Insecta	Diptera	Drosophilidae	*Scaptomyza*	*S. flava*	RKRM01000564.1	12	50	32	19200
Insecta	Diptera	Tephritidae	*Bactrocera*	*B. tryoni*	JHQJ01000220.1	12	54	36	23328
Insecta	Diptera	Tephritidae	*Bactrocera*	*B. dorsalis*	JFBF01000093.1	12	57	37	25308
Insecta	Diptera	Tephritidae	*Bactrocera*	*B. latifrons*	MIMC01000186.1	12	55	36	23760
Insecta	Diptera	Tephritidae	*Bactrocera*	*B. oleae*	LGAM02035380.1	12	54	36	23328
Insecta	Diptera	Tephritidae	*Ceratitis*	*C. capitata*	CAJHJT010000056.1	12	57	36	24624
Insecta	Diptera	Diopsidae	*Teleopsis*	*T. dalmanni*	NLCU02018416.1	12	55	31	20460
Insecta	Diptera	Glossinidae	*Glossina*	*G. brevipalpis*	JFJS01000594.1	12	44	34	17952
Insecta	Diptera	Glossinidae	*Glossina*	*G. fuscipes*	JACGUE000000000.1	12	39	33	15444
Insecta	Diptera	Hippoboscidae	*Crataerina*	*C. pallida*	CATIXF000000000.1	12	44	30	15840
Insecta	Diptera	Tachinidae	*Thelaira*	*T.solivaga*	CANDYK000000000.1	12	59	38	26904
Insecta	Diptera	Tachinidae	*Thecocarcelia*	*T. acutangulata*	CAJZCA000000000.1	12	56	40	26880
Insecta	Diptera	Tachinidae	*Epicampocera*	*E. succincta*	CAKOAZ000000000.1	12	58	38	26448
Insecta	Diptera	Tachinidae	*Trichopoda*	*T. pennipes*	JASKUP000000000.1	12	58	40	27840

Insecta	Diptera	Tachinidae	*Phasia*	*P. obesa*	CATIVE000000000.1	12	60	40	28800
Insecta	Diptera	Tachinidae	*Cistogaster*	*C. globosa*	CALMVC000000000.1	12	60	37	26640
Insecta	Diptera	Tachinidae	*Gymnosoma*	*G. rotundatum*	CAKAJB000000000.2	12	60	38	27360
Insecta	Diptera	Tachinidae	*Ormia*	*O. ochracea*	CAUJRP000000000.1	12	54	39	25272
Insecta	Diptera	Tachinidae	*Panzeria*	*P. rudis*	CATQFL000000000.1	12	60	41	29520
Insecta	Diptera	Tachinidae	*Gymnocheta*	*G. viridis*	CATQFJ000000000.1	12	54	39	25272
Insecta	Diptera	Tachinidae	*Tachina*	*T. grossa*	CATLJX000000000.1	12	57	39	26676
Insecta	Diptera	Tachinidae	*Tachina*	*T. lurida*	CALYBX000000000.1	12	56	38	25536
Insecta	Diptera	Tachinidae	*Tachina*	*T. fera*	CAJMZS000000000.1	12	55	38	25080
Insecta	Diptera	Tachinidae	*Nowickia*	*N. ferox*	CAKZFG000000000.1	12	55	39	25740
Insecta	Diptera	Tachinidae	*Lypha*	*L. dubia*	CAMZJU000000000.1	12	56	41	27552
Insecta	Diptera	Sarcophagidae	*Wohlfahrtia*	*W. magnifica*	JAKWBJ000000000.1	12	58	44	30624
Insecta	Diptera	Sarcophagidae	*Metopia*	*M. argyrocephala*	CAUYWT000000000.1	12	55	39	25740
Insecta	Diptera	Sarcophagidae	*Sarcophaga*	*S. peregrina*	JABZEU000000000.1	12	63	41	30996
Insecta	Diptera	Sarcophagidae	*Sarcophaga*	*S. caerulescens*	CAKMJM000000000.1	12	62	42	31248
Insecta	Diptera	Sarcophagidae	*Sarcophaga*	*S. rosellei*	CAKNFA000000000.1	12	62	42	31248
Insecta	Diptera	Sarcophagidae	*Sarcophaga*	*S. variegata*	CAKNZP000000000.1	12	63	43	32508
Insecta	Diptera	Sarcophagidae	*Sarcophaga*	*S. subvicina*	CAKZFR000000000.2	12	60	44	31680
Insecta	Diptera	Rhinophoridae	*Melanophora*	*M. roralis*	CAUZWR000000000.1	12	47	36	20304
Insecta	Diptera	Rhinophoridae	*Phyto*	*P. melanocephala*	CALNXD000000000.1	12	61	41	30012
Insecta	Diptera	Rhiniidae	*Stomorhina*	*S. lunata*	CAKOFX000000000.1	12	67	37	29748
Insecta	Diptera	Polleniidae	*Pollenia*	*P. labialis*	CASGGT000000000.1	12	63	41	30996
Insecta	Diptera	Polleniidae	*Pollenia*	*P. angustigena*	CAKNFE000000000.1	12	63	41	30996
Insecta	Diptera	Polleniidae	*Pollenia*	*P. amentaria*	CALSEU000000000.1	12	59	42	29736
Insecta	Diptera	Calliphoridae	*Bellardia*	*B. bayeri*	CATNUC000000000.1	12	63	42	31752
Insecta	Diptera	Calliphoridae	*Bellardia*	*B. pandia*	CAJZHU000000000.2	12	66	43	34056
Insecta	Diptera	Calliphoridae	*Calliphora*	*C. vomitoria*	CALNXL000000000.2	12	63	44	33264
Insecta	Diptera	Calliphoridae	*Protophormia*	*P. terraenovae*	CATOAW000000000.1	12	65	42	32760
Insecta	Diptera	Calliphoridae	*Protocalliphora*	*P. azurea*	CAKNZY000000000.1	12	64	41	31488
Insecta	Diptera	Calliphoridae	*Cochliomyia*	*C. hominivorax*	PYHX00000000.2	12	65	41	31980
Insecta	Diptera	Anthomyiidae	*Leucophora*	*L. obtusa*	CATLKA000000000.1	12	57	40	27360
Insecta	Diptera	Anthomyiidae	*Eustalomyia*	*E. histrio*	CATIYI000000000.1	12	56	41	27552
Insecta	Diptera	Anthomyiidae	*Delia*	*D. radicum*	CAKOAZ000000000.1	12	56	41	27552
Insecta	Diptera	Muscidae	*Musca*	*M. domestica*	JACCFA010000371.1	12	64	45	34560
Insecta	Diptera	Muscidae	*Polietes*	*P. domitor*	CANDYM000000000.1	12	56	47	31584
Insecta	Diptera	Muscidae	*Stomoxys*	*S. calcitrans*	JARAKG000000000.1	12	58	45	31320
Insecta	Diptera	Muscidae	*Hydrotaea*	*H. diabolus*	CAUPSE000000000.1	12	58	38	26448
Insecta	Diptera	Asilidae	*Proctacanthus*	*P. coquilletti*	MNCL01000002.1	13	40	37	19240
Insecta	Diptera	Scatopsidae	*Coboldia*	*C. fuscipes*	JXOR01000085.1	13	29	22	8294
Insecta	Diptera	Anisopodidae	*Sylvicola*	*S. fuscatus*	JANSTS000000000.1	11	44	32	15488
Insecta	Diptera	Psychodidae	*Phlebotomus*	*P. papatasi*	JADFTQ000000000.1	12	33	20	7920
Insecta	Diptera	Cecidomyiidae	*Mayetiola*	*M. destructor*	AEGA01001582.1	10	24	21	5040
Insecta	Diptera	Cecidomyiidae	*Contarinia*	*C. nasturtii*	VYII01000185.1	11	26	15	4290

Insecta	Diptera	Cecidomyiidae	*Sitodiplosis*	*S. mosellana*	VUAH01006190.1	10	17	19	3230
Insecta	Diptera	Ceratopogonidae	*Culicoides*	*C. sonorensis*	OGVF02000098.1	11	35	24	9240
Insecta	Diptera	Ceratopogonidae	*Culicoides*	*C. tainanus*	JAJATT000000000.1	12	31	31	11532
Insecta	Diptera	Ceratopogonidae	*Forcipomyia*	*F. taiwana*	CBTT000000000.1	13	25	19	6175
Insecta	Diptera	Chironomidae	*Polypedilum*	*P. pembai*	JACWBX000000000.1	16	21	27	9072
Insecta	Diptera	Chironomidae	*Polypedilum*	*P. vanderplanki*	JAFFZY000000000.1	16	14	28	6272
Insecta	Diptera	Chironomidae	*Chironomus*	*C. riparius*	CAKJTK000000000.4	16	22	29	10208
Insecta	Diptera	Chironomidae	*Chironomus*	*C. striatipennis*	JANIWZ000000000.1	15	24	29	10440
Insecta	Diptera	Chironomidae	*Chironomus*	*C. tentans*	JAKJNA000000000.1	16	24	35	13440
Insecta	Diptera	Chironomidae	*Chironomus*	*C. tepperi*	JADBJN000000000.1	15	23	32	11040
Insecta	Diptera	Chironomidae	*Propsilocerus*	*P. akamusi*	JPYR00000000.1	13	12	28	4368
Insecta	Diptera	Chironomidae	*Smittia*	*S. sp.YW1114-2*	GCA_033064975.1	14	19	22	5852
Insecta	Diptera	Chironomidae	*Smittia*	*S. aterrima*	GCA_033063855.1	14	19	25	6650
Insecta	Diptera	Chironomidae	*Belgica*	*B. antarctica*	JPYR01000022.1	13	12	21	3276
Insecta	Diptera	Chironomidae	*Clunio*	*C. marinus*	CVRI01000074.1	10	15	20	3000
Insecta	Diptera	Culicidae	*Culex*	*C. quinquefasciatus*	JADDTP010000002.1	14	34	27	12852
Insecta	Diptera	Culicidae	*Aedes*	*A. aegypti*	AAGE02030644.1	14	34	39	18564
Insecta	Diptera	Culicidae	*Anopheles*	*A. stephensi*	JABEOT010000002.1	14	34	33	15708
Insecta	Diptera	Culicidae	*Anopheles*	*A. funestus*	APCI01006061.1	14	30	32	13440
Insecta	Diptera	Culicidae	*Anopheles*	*A. minimus*	APHL01003440.1	14	31	28	12152
Insecta	Diptera	Culicidae	*Anopheles*	*A. epiroticus*	APCJ01000739.1	14	30	36	15120
Insecta	Diptera	Culicidae	*Anopheles*	*A. gambiae*	AAAB01008823.1	14	30	38	15960
Insecta	Diptera	Culicidae	*Anopheles*	*A.quadriannulatus*	APCH00000000.1	14	30	37	15540
Insecta	Diptera	Culicidae	*Anopheles*	*A. arabiensis*	APCN01003445.1	14	30	37	15540
Insecta	Diptera	Culicidae	*Anopheles*	*A. darlingi*	ADMH01001469.1	14	30	35	14700
Insecta	Diptera	Culicidae	*Anopheles*	*A. albimanus*	JADGIR010000004.1	14	30	35	14700
Insecta	Lepidoptera	Salticidae	*Danaus*	*D. plexippus*	DUEA00000000.1	15	28	19	7980
Insecta	Lepidoptera	Nymphalidae	*Melitaea*	*M. cinxia*	CAJNAH000000000.1	14	26	19	6916
Insecta	Lepidoptera	Nymphalidae	*Heliconius*	*H. melpomene*	CAEZ00000000.1	15	24	20	7200
Insecta	Lepidoptera	Sphingidae	*Manduca*	*M. sexta*	JACVES000000000.1	17	27	18	8262
Insecta	Lepidoptera	Bombycidae	*Bombyx*	*B. mori*	BHWX00000000.1	15	27	19	7695
Insecta	Lepidoptera	Crambidae	*Chilo*	*C. suppressalis*	CADHSE000000000.2	15	29	19	8265
Insecta	Lepidoptera	Noctuidae	*Helicoverpa*	*H. armigera*	JAHDYT000000000.1	15	28	18	7560
Insecta	Lepidoptera	Plutellidae	*Plutella*	*P. xylostella*	CAKNZZ000000000.1	15	32	17	8160
Insecta	Coleoptera	Coccinellidae	*Coccinella*	*C. septempunctata*	CAJRAZ000000000.1	9	24	41	8856
Insecta	Coleoptera	Coccinellidae	*Harmonia*	*H. axyridis*	CAJZBN000000000.1	9	25	46	10350
Insecta	Coleoptera	Silvanidae	*Oryzaephilus*	*O. surinamensis*	SSSI00000000.1	9	26	28	6552
Insecta	Coleoptera	Nitidulidae	*Aethina*	*A. tumida*	JALKMD000000000.1	9	36	41	13284
Insecta	Coleoptera	Chrysomelidae	*Leptinotarsa*	*L. decemlineata*	JANJPO000000000.1	8	30	28	5880
Insecta	Coleoptera	Cerambycidae	*Anoplophora*	*A. glabripennis*	AQHT00000000.2	9	31	37	10323
Insecta	Coleoptera	Tenebrionidae	*Tribolium*	*T. castaneum*	AAJJ00000000.2	9	28	31	7812
Insecta	Coleoptera	Curculionidae	*Dendroctonus*	*D. ponderosae*	JAFETG000000000.1	11	26	24	6864
Insecta	Coleoptera	Curculionidae	*Hypothenemus*	*H. hampei*	JABRWK000000000.1	11	21	26	6006

Insecta	Coleoptera	Curculionidae	*Sitophilus*	*S. oryzae*	PPTJ00000000.2	10	38	36	13680
Insecta	Coleoptera	Scarabaeidae	*Onthophagus*	*O. taurus*	JHOM00000000.2	9	36	38	12312
Insecta	Coleoptera	Silphidae	*Nicrophorus*	*N. vespilloides*	LJCH00000000.1	9	31	53	14787
Insecta	Coleoptera	Buprestidae	*Agrilus*	*A. planipennis*	JENH00000000.2	9	26	27	7020
Insecta	Hymenoptera	Athaliidae	*Athalia*	*A. rosae*	CAKJPL000000000.1	8	31	20	4960
Insecta	Hymenoptera	Pteromalidae	*Nasonia*	*N. vitripennis*	WELF00000000.1	7	30	41	8610
Insecta	Hymenoptera	Pteromalidae	*Nasonia*	*N. longicornis*	ADAP00000000.1	7	30	40	8400
Insecta	Hymenoptera	Pteromalidae	*Nasonia*	*N. giraulti*	QLYP00000000.1	7	30	40	8400
Insecta	Hymenoptera	Halictidae	*Nomia*	*N. melanderi*	REGV00000000.1	8	39	17	5304
Insecta	Hymenoptera	Halictidae	*Dufourea*	*D. novaeangliae*	LGHO00000000.1	8	41	18	5904
Insecta	Hymenoptera	Apidae	*Apis*	*A. mellifera*	QIUM00000000.2	8	45	17	6120
Insecta	Hymenoptera	Apidae	*Bombus*	*B. terrestris*	CAJUYY000000000.2	8	45	17	6120
Insecta	Hymenoptera	Apidae	*Bombus*	*B. impatiens*	AEQM00000000.2	8	45	17	6120
Insecta	Hymenoptera	Apidae	*Euglossa*	*E. dilemma*	NIJG00000000.1	8	41	17	5576
Insecta	Hymenoptera	Megachilidae	*Osmia*	*O. bicornis*	CAJRAL000000000.1	8	40	17	5440
Insecta	Hymenoptera	Megachilidae	*Megachile*	*M. rotundata*	JAJHPZ000000000.1	9	40	18	6480
Insecta	Hymenoptera	Formicidae	*Harpegnathos*	*H. saltator*	QANH00000000.1	8	42	17	5712
Insecta	Hymenoptera	Formicidae	*Linepithema*	*L. humile*	ADOQ00000000.1	7	47	17	5593
Insecta	Hymenoptera	Formicidae	*Camponotus*	*C. floridanus*	QANI00000000.1	8	44	17	5984
Insecta	Hymenoptera	Formicidae	*Pogonomyrmex*	*P. barbatus*	ADIH00000000.1	7	47	17	5593
Insecta	Hymenoptera	Formicidae	*Solenopsis*	*S. invicta*	JAEQMK000000000.1	8	47	17	6392
Insecta	Hymenoptera	Formicidae	*Atta*	*A. cephalotes*	ADTU00000000.1	8	31	17	4216
Insecta	Hymenoptera	Formicidae	*Acromyrmex*	*A. echinatior*	JANHNX000000000.1	8	47	17	6392
Insecta	Hemiptera	Aphididae	*Acyrthosiphon*	*A. pisum*	SDIB00000000.1	17	21	18	6426
Insecta	Hemiptera	Cicadellidae	*Homalodisca*	*H. vitripennis*	JAJKGG000000000.2	13	37	26	12506
Insecta	Hemiptera	Delphacidae	*Nilaparvata*	*N. lugens*	WOVF00000000.1	11	47	9	4653
Insecta	Hemiptera	Pentatomidae	*Halyomorpha*	*H. halys*	JMPT00000000.3	21	43	18	16254
Insecta	Hemiptera	Reduviidae	*Rhodnius*	*R. prolixus*	ACPB00000000.3	8	39	20	6240
Insecta	Hemiptera	Cimicidae	*Cimex*	*C. lectularius*	JHUN00000000.2	13	38	24	11856
Insecta	Phthiraptera	Pediculidae	*Pediculus*	*P. humanus*	JARDYY000000000.1	9	18	13	2106
Insecta	Phthiraptera	Philopteridae	*Columbicola*	*C. columbae*	JADMLL000000000.1	9	24	16	3456
Hexanauplia	Siphonostomatoida	Caligidae	*Lepeophtheirus*	*L. salmonis*	JADKYV000000000.2	16	26	18	7488
Hexanauplia	Harpacticoida	Harpacticidae	*Tigriopus*	*T. californicus*	VCGU00000000.1	15	19	13	3952
Hexanauplia	Harpacticoida	Tisbidae	*Tisbe*	*T. holothuriae*	CAACVA000000000.1	20	35	14	9800
Hexanauplia	Harpacticoida	Harpacticidae	*Tigriopus*	*T. kingsejongensis*	JABCAI000000000.1	15	22	13	4290
Hexanauplia	Cyclopoida	Cyclopettidae	*Paracyclopina*	*P. nana*	JAGUCH000000000.1	16	19	15	4560
Hexanauplia	Notostraca	Triopsidae	*Triops*	*T. cancriformis*	JAGQDJ000000000.1	9	24	25	5400
Hexanauplia	Notostraca	Triopsidae	*Lepidurus*	*L. apus*	RJJB00000000.2	9	27	23	5589
Malacostraca	Decapoda	Varunidae	*Eriocheir*	*E. sinensis*	JAHNEI000000000.1	26	58	21	31668
Malacostraca	Decapoda	Lithodidae	*Paralithodes*	*P. platypus*	JAVRFV000000000.1	30	102	20	61200
Malacostraca	Decapoda	Portunidae	*Portunus*	*P. trituberculatus*	JAGGDN000000000.1	27	64	21	36288
Malacostraca	Decapoda	Penaeidae	*Penaeus*	*P. vannamei*	QCYY00000000.1	23	87	28	58464
Malacostraca	Decapoda	Penaeidae	*Penaeus*	*P. monodon*	JACBPZ000000000.1	26	81	25	54756

Malacostraca	Decapoda	Penaeidae	*Penaeus*	*P. chinensis*	JABKCB000000000.1	25	94	29	68150
Malacostraca	Decapoda	Penaeidae	*Penaeus*	*P. indicus*	JAGYIC000000000.1	26	87	33	74646
Malacostraca	Decapoda	Penaeidae	*Penaeus*	*P. japonicus*	JAGTTE000000000.1	25	71	28	49700
Malacostraca	Decapoda	Cambaridae	*Procambarus*	*P. clarkii*	JAIWQB000000000.1	27	61	26	42822
Malacostraca	Decapoda	Nephropidae	*Homarus*	*H. americanus*	JAHLQT000000000.1	23	73	25	41975
Malacostraca	Isopoda	Cirolanidae	*Bathynomus*	*B. jamesi*	JAJOZX000000000.1	17	38	17	10982
Branchiopoda	Diplostraca	Daphniidae	*Daphnia*	*D. magna*	JAOYFB000000000.1	8	24	17	3264
Branchiopoda	Diplostraca	Daphniidae	*Daphnia*	*D. pulex*	JAHCQT000000000.1	8	26	16	3328
Branchiopoda	Diplostraca	Daphniidae	*Daphnia*	*D. sinensis*	WJBH00000000.2	8	27	17	3672
Branchiopoda	Diplostraca	Daphniidae	*Daphnia*	*D. pulicaria*	JAJAGA000000000.1	10	27	17	4590
Branchiopoda	Diplostraca	Daphniidae	*Daphnia*	*D. obtusa*	JAACYE000000000.1	8	27	17	3672
Branchiopoda	Diplostraca	Daphniidae	*Daphnia*	*D. galeata*	CAKKLH000000000.1	8	25	16	3200
Branchiopoda	Diplostraca	Limnadiidae	*Eulimnadia*	*E. texana*	NKDA00000000.1	7	20	16	2240
