# Supplementary material for: The modern expansion of Dscam1 isoform diversity in Drosophila is linked to fitness and immunity
Source: PLoS Biol. 2025 Sep 12;23(9):e3003383. doi: 10.1371/journal.pbio.3003383 (PMC12431208; doi:10.1371/journal.pbio.3003383)
Supplement: S2 Table — (PDF) [file pbio.3003383.s014.pdf]

## Table S2 Summary of the mutation sequences of *Dscam1* mutants

| Mutants | Mutation sequences* | |
|---|---|---|
| ***Dscam*$^{\Delta4.10}$** | CGCAGAGACGG4.9gtacttggccaggtttctttgccgtagctctctcaggct~~cctctatagctatccagaaacccat~~<br>~~tcccgttcacttcggttattatttcaccaagttcgctctgatttccttcagTTGTCCACCAGTTCTACCAGACA~~<br>~~CGGGTCATCGATGAGTTTGTGCTGCGTGGCAACTCCGCCACCTTGAAGTGCT~~<br>~~TGGTGCCCTCGTTTGTGGCAGACTTCATCGATGTCGAGGGTTGGATCGACGA~~<br>~~GGAGGGCGTGGAGATCCTGCGGCCCCTCCCGGACGACTCCGTTG~~4.10~~gtaatca~~<br>~~ctacgactttcggcattagcatagttagagtacgaaaccttcaacccaccgaccct~~tccctgtcccgatttttagtttctcacctcc<br>cgtcttgcagTGGTGAAGCAGTTTTTCGAGTCGCAAGTCTAC4.11 | In this paper |
| ***Dscam*$^{\Delta4.3\text{-}4.6}$** | CACTGAATATG4.2gtgtgtgactagaagaacagtctctgtttgtgagcttttagaaactatcactaccttaaccgcta<br>ctgtacaacactgcaaaaaaggcaaactcactgaaacacttgtgcacagtgcaagggcagttaacgggaaaacttgcaaca<br>ctaattacaaaattctgtaaaatatacaacacataacataacaag~~aataacgtattaaccaacactggctaggcagaaaccagt~~<br>~~cgagaatccttcgctccctcgtcgta~~……~~AGTCCTGGATAGATGAGGAAGGCACCGAACTG~~<br>~~CG~~TCCCTCTGAGAACTATG4.6gtattaccgcagagtcaaaaattcttgcaacggcaacactttgtggg | (Wang et al., 2004) |
| ***Dscam*$^{\Delta4.4\text{-}4.12}$** | GATCAGAACGAGGACTTTCTGCCAGGCAGCGAATACG4.3gtttgttggccaactttttgatt<br>acattttgggctagctatacatacatagggtttgtacagttgcgtgccacctaagaattatggataagtacctaaccattttccctc<br>ctgcatctattatgtttacttttatgcgtttatcttcgctatctcgggtgactgcgtgttgatgagccatgttatcaaaaccattattaa<br>cctcttgcccggagattttctcagggaagcgcggagctcgcgagctgattgcgcttagggtcccattaagtgcactccgcgat<br>attgtctgcgc~~tgttagaagacactctcgtttcaattagctagcttcacatttt~~……~~ttgttcgccgtactgtattgttaataagtta~~<br>~~agttagccgacaattcatcactaacatattctttattctctatctgcacatatcaaaatat~~cagATGGAAAGTACCTG5 | (Wang *et al.*, 2004) |
| ***Dscam*$^{\Delta6.22}$** | TCGTATCCAATCCCAGCTTTTAG6.21gtaaacacttaagatttaaattgtccagtacgtgaaacc~~attctg~~<br>~~gcagatttttgaatcaaaactaagataaaacctcccacagAACCCATTGGTAGTGTTGCTCCCCGAG~~<br>~~TAAATCGCAAGGATGAGTTCAATCACGACCGTATAAAGAGCTCGAAGACAA~~<br>~~TTTCCATACAATGTCCAGCTCAGGCCTTTCCGGTTCC~~GGTTTATAG6.22 | In this paper |
| ***Dscam*$^{\Delta6.2\text{-}6.10}$** | gctcgccttagttgttattagcataatccagcataattagcttcctctttcaagAACCCAT~~TGCGAGTGTGGGG~~<br>~~CCCAGACTTCTCTCTGGTAATGACATTAAGGTGCTTCAGTTCTCTGCGAGCC~~<br>~~AAGCCAGCACCCTCTTGTGTCCAGCTCAATCATATCCAGTGCCAGTCTTTAG~~<br>6.2GTGAATCCCAGGGAACCCAGGGAATCGGAATCGCTCTACTGTGTCCGGC<br>GCAGTCATATCCGATTCCAGCCCACAG6.10gttt | In this paper |
| ***Dscam*$^{\Delta9.1}$** | gtccatcgtgcttctctcaatcgaatacccaaacctctcgctcagTTCCAC~~CGCAGGTCGTACCCTTTGA~~<br>~~TTTCGGTGAGGAAACCATCAACATGAATGACATGGTCTCGGCCACGTGCAC~~<br>~~AGTGAACAAGGGCGACACTCCCCTGGAGCTGTACTGGACAACGGCTCCGG~~<br>~~ATCCCACGACGGGAGTGGGACGCCTGATGTCCAACGATGGCATTCTAATCAC~~<br>~~AAAGACGACGCAGCGCATCAGCATGCTGAGCATAGAGTCCGTGCATGCTCG~~<br>~~CCATCGGGCGAACTACACGTGTGTGGCCAGGAATGCGGCCGGGGTCATCTA~~<br>~~CCAC~~ACGGCAGAGCTGCGCGTTAACG9.1gttcgcttaggccttcttcgattcg | In this paper |
| ***Dscam*$^{\Delta9.6}$** | AACG9.5gta~~catggacccagggacctcactctaagctcctttattattatttgcttatcataattttatttactttaattttatttac~~<br>~~acctttcgaaatctttagTACCTCCCCATGTCCTGCCCTTCAGTTTCGGCAGCGAAGTCT~~<br>~~TTAATATGGGCGATGTTTTGAGCATCACTTGTGTTGTCCTCAAGGGGGACCT~~<br>~~GCCCTTGAGAATCCACTGGACCTTGAATGGTGAGCCTGTGGCAACAGGTGT~~<br>~~GAATGGATTCACAGTAATGCAGTTGAATCAACGTACCACCTACCTGAGTGTG~~<br>~~GATGCACTGGAGGCCAAGCATCGCGGCTCCTATAGCTGCGTGGCTCAGAATC~~<br>~~AGGCGGGTGAAGCGATTTACTCCGCTGACCTGCAAGTCAATG~~9.6~~gttggtgaaact~~<br>~~ccctttggatctctctttatatccgcctgta~~aagttgcttcctactaacacgatcctggtttatttggtggctaagagctttgtat | In this paper |
| ***Dscam*$^{\Delta9.9}$** | ttaatttaccacttatttcct~~gtccatttaagTGCCGCCCCAGGT~~TTTGCCCTTTAGTTTCGGCGA<br>ATCCGCCGCCGATGTCGGCGATATTGCCAGTGCCAACTGTGTGGTGCCCAAG<br>GGAGATCTGCCCCTGGAGATTCGCTGGTCCCTC9.9 | In this paper |
| ***Dscam*$^{\Delta9.13}$** | agTTTTGCCCCAAATTGTGCCCTTCGCC~~TACGAGGATCTGATCAATATGGGCG~~<br>~~ACTCGATAGATTTGTTTTGCCAAATCCAAAAAGGCGACCGTCCCATCAAGGT~~<br>~~GCACTGGAGTTTCGAGCGGAGCGCTGGAGACTACGGCTTTGATCAGGTGCA~~<br>~~GCCCCAGATGCGCACGAATCGCATTAGCGAGAAGACGAGCATGATCTCCATT~~<br>~~CCCAGTGCCAGTCCTGCCCACACCGGCCGGATACCTGTATAGCCAGTAATAA~~<br>~~GGCTGGAACTACAACATATAGCGTTGACCTGACAGTGAACG~~9.13~~gtactgaaattga~~<br>~~gtgtgtcatgatttagttttagtggttgcggttactaaaggtcttgctagttgttgctactaatcaaacccatgcccatttctctaactt~~ | In this paper |
| ***Dscam*$^{\Delta9.24}$** | gcactcaattcacacaaaagCCCCACCGAAACTTGCTCCTTTTGACTTTGGTGACTCCC<br>C~~TGCTAACTTCGAGGATTCCGTTTCGGTTAGCTGCCTCGTCTCTTCTGGTGAT~~<br>~~TTGCCCATTGACATCGAGTGGCTGTTCAACGGGGAATCCATAAGCTACGCCT~~<br>~~CGGGAATCGCTGTTTTGCGAGGTGGCAAAAGGACTAGTGTACTCACGATAG~~<br>~~ATTCCGTTCATGCGGGACATGCGGGGAACTACAGCTGCAAGGCCAAGAACA~~<br>~~AAGCGGCCAGCAGTG~~GAGTATACTCGGTGACTTTGGTGACTTTGGAATACTC<br>GGCGGCCCTTATTGTCAATG9.24gtcattagattatgcttataaaaatccgtcgctgtt | In this paper |

| | | |
|---|---|---|
| ***Dscam*$^{\Delta 9.29}$** | gttcgttgagttgactgcttttgttttgtgtatcccacaagTCTTGCCACAAATCACGCCCTTT~~GCCACCGGTTCGCAGCCCACCCATCTGGGCCAGTATATAACGTACCAGTGCACCCTGACGGAGGGCGACTTGCCCCTCAACATCCGTTGGACGTTTAACAACCAACCGCTCTTCAATGACGATGACCAGGACATCCTCATAGCGAAGATGGGTCGTCGAAGTAGTGTTCTGACCATCGAAAGTGTGGCAGATCGTCACGCGGGCAACTACAGTTGCCACGGGGAAAACGCCGCCGGTCGTGCCACCTACAGCACCCAGCTGCGTGTGATAG~~9.29~~gttctcatcccagcgtcttagtatt~~aagtgaagctttcgttgatgcggttatgttttaattttatgcacc | In this paper |
| ***Dscam*$^{\Delta 9.30}$** | gttctcatcccagcg~~tcttagtattaagagaagctttcgttgatgcggttatgttttaattttatgcaccttttgttttaccatcacatcactttaccgaaacgcacatccagTTCTTCCGCGCATCATACCCTTCGCCTTCGAGGAGGGTCCTGCCCAGGTGGGTCAGTACCTGACACTGCACTGCTCCGTTCCCGGCGGAGATCTGCCCCTGAACATCGACTGGACGCTGGATGGTCAGGCAATCTCGGAGGATCTGGGAATAACCACGTCCCGCGTGGGTCGTCGTGGAAGTGTGCTAACCATCGAGGCCGTGGAGGCCTCACATGCGGGCAACTTCACCTGCCACGCCCGCAATTTGGCGGGCCATCAGCAGTTCACCACGCCGCTGAACGTCTACG~~9.30~~gttaggaattctccg~~TTCCCAGGGTCATCTggattgggtcgctcgagtagtttctttgtggccgattactaacctagcgt | In this paper |
| ***Dscam*$^{\Delta 9.31}$** | gttaggaattctccg~~ggattgggtcgctcgagtagtttctttgtggccgattactaacctagcgtcgctgcatgaccgtctgtctctgctgctgtcctgacttatttttccgtctttctgaccgacttagacccatcactcctctttggaatggcctgacgcacatacacactgaaagcttgcatgtatgatacccagactgcactgaactgttctgaccagaataggctacgtttcagagatacaaaaactaaaaacaaaaactaattttctctagttattgctatcgtataaggtataaagcagataatacctcgagttccaaactgattagaaatcttaaaagtttccctctcgaaaggaagttgtaaagacgaacttgagatgttaataagtgtccattataatggaaatgagataggggcccttcgtcaaccgaattatattttatgtttaacttttatttctcccttttttattggaaccatacaatgcagTGCCCCCCAAACTGGCTCCCTTGCCCGTAAATTCGCCACTGTACGTGGGCGACTACTACCAGTTGACGTGCGCGGTGGTGCACGGCGACGCACCCTTCAACATTACCTGGTACTACAACAACGAGCCCGCCGGAGACTTGGCCGGGGTCACCATCCTGATGCACGGCCGGCGCAGCAGCTCGCTGAACATCGAGAGCGTGGGGGGTGACCACGCGGGCAACTACACCTGCAAGGGCGCCAACCGGGCGGGCGAAACCACGGCTGAGACCCACCTGAGTGTCAAGG~~9.31~~gtttgtgcgaattactcctaaggaatcgctagctgggggtggcgtcttagtcgtagattggaatcctagaatagga~~ACAATtcaaggcatctgtaaatccccactctctgtctgtgcctccttgtctggtgttggtactgcataagcacacgaacctgt | In this paper |
| ***Dscam*$^{\Delta 9.33}$** | acaggaatgtcttcttaatgattaaatcatatcaaattcgtatcactaataaattaagcatcttcagtttgaaaaaagggttttccatc~~gacgcagactgaaccgactgcactcactcaactttccatgaatgacttactttatgacctgaaattttaataatggcacccccttcctgttccctcaaaaaaaaaaatatcccaagAACCACCTATGCTCTCGCCACTGCTGCTTTCCGCTTCCGTTTGCGATGTCGGCGACTACGTGCAACTCACCTGCATTGCCAGCCGGGGGTCCACGCCCATTATGTTCGAATGGTGGCTAGATGGGCGTCAGGTGCACGATGATGGCAACGATGACGTCACTCAGGCCACAGTTGGCGAACACACCAGCCTACTGCTGATCAACAGGGCGGGGGAGGGGCATGCGGGGAACTACAGTTGTCGGGCGATCAACCAGGTGGGCGTGGCAGAACGCCGGACCAGGCTAACTG~~TGAATG9.33gtaaatgggcggttatcagattatcagatatgccaattccaaattacttttgactcctcttgc | In this paper |
| | **Mutants** | **Reference** |
| **Exon 4 cluster** | *Dscam*$^{\Delta 4.10\text{-}4.12}$, *Dscam*$^{\Delta 4.1\text{-}4.5}$, *Dscam*$^{\Delta 4.6\text{-}4.12}$, *Dscam*$^{\Delta 4.1\text{-}4.9}$, *Dscam*$^{\Delta 4.1\text{-}4.10}$, *Dscam*$^{\Delta 4.3\text{-}4.12}$ | (Dong et al., 2023) |
| | *Dscam*$^{Single4.1}$, *Dscam*$^{Single4.2}$, *Dscam*$^{Single4.3}$, *Dscam*$^{Single4.4}$, *Dscam*$^{Single4.6}$, *Dscam*$^{Single4.7}$, *Dscam*$^{Single4.9}$, *Dscam*$^{Single4.10}$, *Dscam*$^{Single4.11}$, *Dscam*$^{Single4.12}$ | (Dong et al., 2022) |
| **Exon 6 cluster** | *Dscam*$^{\Delta 6.36}$, *Dscam*$^{\Delta 6.21\text{-}6.30}$, *Dscam*$^{\Delta 6.10\text{-}6.20}$, *Dscam*$^{\Delta 6.30\text{-}6.48}$, *Dscam*$^{\Delta 6.10\text{-}6.30}$, *Dscam*$^{\Delta 6.1\text{-}6.30}$, *Dscam*$^{\Delta 6.17\text{-}6.48}$, *Dscam*$^{\Delta 6.2\text{-}6.35}$, *Dscam*$^{\Delta 6.8\text{-}6.47}$, *Dscam*$^{\Delta 6.5\text{-}6.44}$, *Dscam*$^{\Delta 6.7\text{-}6.47}$, *Dscam*$^{\Delta 6.4\text{-}6.44}$, *Dscam*$^{\Delta 6.6\text{-}6.47}$, *Dscam*$^{\Delta 6.3\text{-}6.44}$, *Dscam*$^{\Delta 6.5\text{-}6.47}$, *Dscam*$^{\Delta 6.1\text{-}6.44}$, *Dscam*$^{\Delta 6.4\text{-}6.47}$, *Dscam*$^{\Delta 6.3\text{-}6.47}$, *Dscam*$^{\Delta 6.2\text{-}6.47}$ | (Dong *et al.*, 2023) |
| | *Dscam*$^{Single6.1}$, *Dscam*$^{Single6.2}$, *Dscam*$^{Single6.4}$, *Dscam*$^{Single6.6}$, *Dscam*$^{Single6.7}$, *Dscam*$^{Single6.8}$, *Dscam*$^{Single6.12}$, *Dscam*$^{Single6.13}$, *Dscam*$^{Single6.16}$, *Dscam*$^{Single6.28}$, *Dscam*$^{Single6.35}$, *Dscam*$^{Single6.43}$, *Dscam*$^{Single6.44}$, *Dscam*$^{Single6.46}$, *Dscam*$^{Single6.48}$ | (Dong *et al.*, 2022) |
| **Exon 9 cluster** | *Dscam*$^{\Delta 9.7}$, *Dscam*$^{\Delta 9.30\text{-}9.33}$, *Dscam*$^{\Delta 9.10\text{-}9.18}$, *Dscam*$^{\Delta 9.7\text{-}9.18}$, *Dscam*$^{\Delta 9.24\text{-}9.33}$, *Dscam*$^{\Delta 9.14\text{-}9.24}$, *Dscam*$^{\Delta 9.1\text{-}9.14}$, *Dscam*$^{\Delta 9.14\text{-}9.29}$, *Dscam*$^{\Delta 9.14\text{-}9.33}$, *Dscam*$^{\Delta 9.2\text{-}9.24}$, *Dscam*$^{\Delta 9.1\text{-}9.24}$, *Dscam*$^{\Delta 9.1\text{-}9.25}$, *Dscam*$^{\Delta 9.2\text{-}9.27}$, *Dscam*$^{\Delta 9.2\text{-}9.28}$, *Dscam*$^{\Delta 9.2\text{-}9.29}$, *Dscam*$^{\Delta 9.1\text{-}9.28}$, *Dscam*$^{\Delta 9.1\text{-}9.29}$, *Dscam*$^{\Delta 9.2\text{-}9.30}$, *Dscam*$^{\Delta 9.1\text{-}9.30}$ | (Dong *et al.*, 2023) |
| | *Dscam*$^{Single9.1}$, *Dscam*$^{Single9.9}$, *Dscam*$^{Single9.13}$, *Dscam*$^{Single9.32}$ | (Dong *et al.*, 2022) |

*The variable exon sequences are in capital letters and highlighted in green. Intron sequences are lowercase letters. The sequence shown in red font and strikethrough represents the deleted sequence. Sequences highlighted in blue indicate inserted sequences.
