# Supplementary material for: The modern expansion of Dscam1 isoform diversity in Drosophila is linked to fitness and immunity
Source: PLoS Biol. 2025 Sep 12;23(9):e3003383. doi: 10.1371/journal.pbio.3003383 (PMC12431208; doi:10.1371/journal.pbio.3003383)
Supplement: S3 Table — (PDF) [file pbio.3003383.s015.pdf]

Table S3 Specific primers used for sgRNA and mutant screening		
**Mutants**	**Screening primers**	**sgRNA Primers**
Dscam$^{\Delta 4.10}$	Ds-4.10-F: CCGAACAATGCCGCAGAGAC<br>Ds-4.10-R: GCCGCATTGCCCTTGATCAC	Ds-4.10-sg-F1: TAGCTCTCTCAGGCTCCTCT<br>Ds-4.10-sg-F2: ACCGACCCTTCCCTGTCCCG
Dscam$^{\Delta 6.22}$	Ds-6.22-F: TGGCAGTGTGGGACCCAAGG<br>Ds-6.22-R: GTGCCGGACATCGAAGGGCC	Ds-6.22-sg-F1: TCCAGTACGTGAAACCATTC<br>Ds-6.22-sg-F2: AGCTCAGGCCTTTCCGGTTC
Dscam$^{\Delta 6.2\text{-}6.10}$	Ds-6.2-F: CTCGCCTTAGTTGTTATTAG<br>Ds-6.10-R: CGAATACCGCCCTGTAAATT	Ds-6.10-sg-F1: ATTGCGAGTGTGGGGCCCAG<br>Ds-6.10-sg-F2: AGGGAATCGGAATCGCTCTA
Dscam$^{\Delta 9.1}$	Ds-9.1-F: TTCCCCAGAATCTGGGAATGCT<br>Ds-9.1-R: AAGGGCACGATCTGGGGCAGAA	Ds-9.1-sg-F1: TCAAAGGGTACGACCTGCGG<br>Ds-9.1-sg-F2: GGCCGGGGTCATCTACCACA
Dscam$^{\Delta 9.6}$	Ds-9.6-F: GAATTACCATACTGAAACTGTC<br>Ds-9.6-R: TTGACACTAGCCACTTCTC	Ds-9.6-sg-F1: AGCTGAAAGTCAACGGTACA<br>Ds-9.6-sg-F2: GTAGGAAGCAACTTTACAGG
Dscam$^{\Delta 9.9}$	Ds-9.9-F: TCATTATGCAAATCATACTC<br>Ds-9.9-R: ATAGACAGTAAGCGTGCTC	Ds-9.9-sg-F1: CTAAAGGGCAAAACCTGGGG<br>Ds-9.9-sg-F2: TGCATAGCCACAAATCCGGC
Dscam$^{\Delta 9.13}$	Ds-9.13-F: AGCAACAATGCGGGGAAC<br>Ds-9.13-R: TAGTTCAGAGGCTCTTC	Ds-9.13-sg-F1: AATTGTGCCCTTCGCCTACG<br>Ds-9.13-sg-F2: TCTATCTAACTAAGCCTGCG
Dscam$^{\Delta 9.24}$	Ds-9.24-F: TCGAGCGAGAAATCATGCGTCT<br>Ds-9.24-R: TTCGTCGGAGCGTCACCGAAGT	Ds-9.24-sg-F1: TGACTCCCCTGCTAACTTCG<br>Ds-9.24-sg-F2: GGCCGCCGAGTATTCACTGC
Dscam$^{\Delta 9.29}$	Ds-9.29-F: ATCGGCAAGAAGGTCAATGTG<br>Ds-9.29-R: TCCGCCGGGAACGGAGCAGTG	Ds-9.29-sg-F1:GGGCTGCGAACCGGTGGCAA<br>Ds-9.29-sg-F2: AGCGTCTTAGTATTAAGAGA
Dscam$^{\Delta 9.30}$	Ds-9.30-F: GTCGTGCCACCTACAGCAC<br>Ds-9.30-R: GTCAGGACAGCAGCAGAGAC	Ds-9.30-sg-F1: TCTCTTAATACTAAGACGCT<br>Ds-9.30-sg-F2: GGTTAGGAATTCTCCGGGAT
Dscam$^{\Delta 9.31}$	Ds-9.31-F: AATAACCACGTCCCGCGTGGGT<br>Ds-9.31-R: GAATGAACACACTGTACAGCG	Ds-9.31-sg-F1: GGTTAGGAATTCTCCGGGAT<br>Ds-9.31-sg-F2: GGAATCCTAGAATAGGATCA
Dscam$^{\Delta 9.33}$	Ds-9.33-F: CACTTCCCTTACAGGAATGTCTT<br>Ds-9.33-R: CTTGGTCTTTGGCCTACGGATGT	Ds-9.33-sg-F1: GTCGGTTCAGTCTGCGTCGA<br>Ds-9.33-sg-F2: GACCAGGCTAACTGTGAATG

Specific primers used for RT-PCR		
**Primers**	**5'-3' sequence**	**Assay**
Ds-3-F	TGGATCAGGAGCGACGGTAC	RT-PCR
Ds-5-R	CTCCAGAGGGCAATACCAGG	RT-PCR
Ds-5-F	GCTACCAGTGCCGAACCAAACATC	RT-PCR
Ds-7-R	AGTCTCAACGCTTTCGCCTCCCAC	RT-PCR
Ds-7-F	AACATAACCTCGGTCCACGC	RT-PCR
Ds-8-R	GTCGCTTGGTCTGAGTTCCG	RT-PCR
Ds-8-F	ACTTGCGTTGCCAAGAATCAGGAAG	RT-PCR
Ds-10-R	GCCTTATCGGTGGGCTCGAGGATCC	RT-PCR
Ds-10-RT	GGTTTGGGGAAGCCATCAGCCTT	RT

Specific primers used for RT-qPCR		
**Primers**	**5'-3' sequence**	**Assay**
Actin-F	GCGTCGGTCAATTCAATCTT	RT-qPCR
Actin-R	AAGCTGCAACCTCTTCGTCA	RT-qPCR
Sfp23F-F	TTTACTTCTTCTCGGCAACC	RT-qPCR
Sfp23F-R	GCCAAACAACTCAACAGCAC	RT-qPCR
Sfp33A4-F	TTCCTATTGCGTTTTGCTTG	RT-qPCR
Sfp33A4-R	GTTTATCGCATCTACTTTCC	RT-qPCR
Jhbp13-F	CAATGGCGACAAGGTGAAAC	RT-qPCR
Jhbp13-R	GAGGCGGAAAAACCGATGAC	RT-qPCR
Spn42Dd-F	CGCCCTGGAAGAGAAGATCG	RT-qPCR
Spn42Dd-R	TCACCTCCAGAAACGCCTTG	RT-qPCR
Ag5r2-F	ACAACACCGACGCCTTCAAG	RT-qPCR
Ag5r2-R	TCCTCTCCGACATCATCACG	RT-qPCR
Spn47C-F	ATGAAGGAAGTGGATGTAAC	RT-qPCR
Spn47C-R	TGCTTCTGGTGCTACTTCGC	RT-qPCR
CG44008-F	TACGGAAAAGGTGGTGAGGC	RT-qPCR
CG44008-R	CTTCTTTGGCTTGTGTGTCG	RT-qPCR
Nlaz-F	CCTGTTGCTGCTTATCTCCG	RT-qPCR
Nlaz-R	TGTAGTTGGCGTAGATGCAC	RT-qPCR
